# Automated Glycan Assembly of Lipopolysaccharide Epitopes for Vaccine Design

**DOI:** 10.1021/jacs.5c08663

**Published:** 2025-07-10

**Authors:** Sabrina Omoregbee-Leichnitz, Emelie E. Reuber, Fabienne Weber, Kim N. Stolte, José Danglad-Flores, Henrik Dommisch, Peter H. Seeberger

**Affiliations:** † 9166 Freie Universität Berlin, Institute of Chemistry and Biochemistry, Berlin 14195, Germany; ‡ Max Planck Institute of Colloids and Interfaces, Potsdam 14476, Germany; § Department of Periodontology, Oral Medicine and Oral Surgery, Institute for Dental and Craniofacial Sciences, 14903 Charité - Universitätsmedizin Berlin, Corporate Member of Freie Universität Berlin, Humboldt-Universität zu Berlin, and Berlin Institute of Health, Berlin 14197, Germany

## Abstract

() is the major cause of chronic periodontitis and is associated with systemic diseases, such as rheumatoid arthritis. A better understanding of the interplay of the human immune system and lipopolysaccharide (LPS) may result in novel treatment and prevention strategies. Twelve LPS fragments of were synthesized using automated glycan assembly, with the help of remote benzoyl ester participation to introduce 1,2-*cis*-glycosidic linkages. Saliva and sera derived from periodontitis patients, treated periodontitis patients, and healthy patients were screened for IgG and IgA antibodies using glycan microarrays. A tetrasaccharide antigen, α-d-Galp-(1→6)-α-d-Glcp-(1→4)-α-l-Rhap-(1→3)-2-β-d-GalNAc was identified as a glycoconjugate vaccine lead against and elicited strong glycan-specific IgG responses in mice, showing binding to W50 and suggesting potential for both targeted protection and broader prevention of systemic diseases linked to periodontitis.

## Graphical abstract

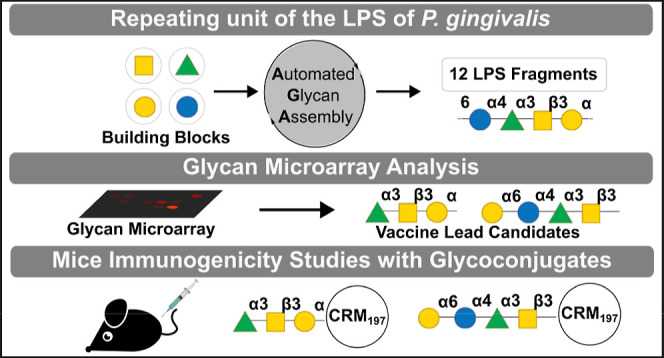

## Introduction

 is considered as one of the keystone pathogens in the etiopathology of periodontitis^1–7^ and is associated with systemic diseases such as atherosclerosis, Alzheimer’s disease, and rheumatoid arthritis.^2,8–15^ strains vary in virulence, with some classified as virulent, e.g., strains W50, ATCC 49417, and A7A1, and others classified as avirulent, e.g., strains 381, 33277, and 23A4. W50 is the most common type of worldwide.^16^

Efforts have focused on identifying effective strategies to control , not only to improve periodontal health but also to mitigate its impact on systemic diseases. Approaches range from mechanical plaque removal for disrupting the disease-triggering dysbiotic biofilm to novel immunization strategies and targeted pharmacological interventions.^14,17–19^ Advances in mucosal vaccination strategies might provide periodontal disease control by reducing the ability of the bacteria to translocate to remote tissues.^20–25^ Sublingual immunization with a recombinant vaccine that contains heat shock protein 60 or nasal immunization with outer membrane vesicles prior to injection significantly reduced arteriosclerotic lesions in mice.^26,27^ The nasal immunization with outer membrane vesicles in mice resulted in a significant increase in -specific IgA in the nasal lavage fluid and saliva of mice, as well as serum IgG and IgA.^23^ The LPS of is essential for its virulence and immunogenicity and is a significant antigen in periodontitis patients.^28–30^ Glycoconjugate vaccines targeting LPS are promising options for disease prevention. Several licensed glycoconjugate vaccines have been shown to be highly efficient in preventing infectious diseases, such as pneumococci or type b.^31^ LPS is the major component of the outer bacterial membrane. This complex glycolipid is composed of three covalently linked domains (Figure 1): The lipid A or endotoxin, the central oligosaccharide core, and the terminal O-antigen.^32^ The glycan portion of the O-antigen (O-LPS) of W50 is composed of the tetrasaccharide repeating sequence[→6)-α-d-Glc*p*-(1→4)-α-l-Rha*p*-(1→3)-β-d-GalNAc-(1→3)-α-d-Gal*p*-(1→] and bears nonstoichiometric amounts of monophosphoethanolamine residue at position C2 of the α-rhamnose (Figure 1).^33^

**Figure 1:**
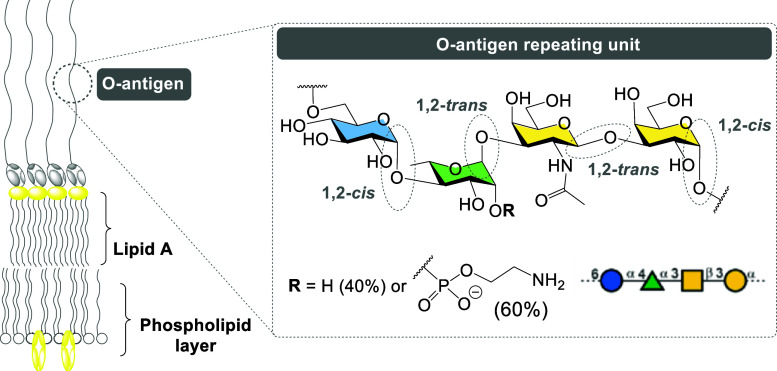
Schematic representation of the outer membrane and chemical structure of the O-antigen repeating unit of LPS (W50 strain).

The synthesis of LPS fragments of is important for the systematic investigation of its biological and immunological roles. The importance of the order of glycans in the LPS repeating unit and antibody binding to specificity has not yet been investigated. Therefore, synthetic LPS glycan fragments are essential for screening antibody binding by using glycan microarrays.

Automated glycan assembly (AGA) provides fast and reliable access to conjugation-ready oligosaccharides. An efficient synthesis of the tetrasaccharide repeating unit of the LPS of requires stereocontrol as two of the four units are connected via 1,2-*cis*-glycosidic linkages.^34,35^ The stereoselective AGA of LPS fragments is the basis for identifying a potential vaccine candidate by screening the saliva and serum of periodontitis patients, treated periodontitis patients, and healthy controls for IgG and IgA antibodies that bind LPS fragments.

## Results and Discussion

### Synthetic Strategy

Retrosynthetic analyses of conjugation-ready tetrasaccharide combinations of the repeating unit of the LPS identified building blocks **2–5** and Merrifield resin **1** equipped with a photocleavable aminopentanol linker (Scheme 1). Fmoc was chosen as a temporary protecting group for chain elongation due to its efficient removal in AGA. The trichloroacetyl (TCA) group at the C2-amine in GalNAc building block **2** and the benzoyl ester at C2–OH in rhamnose building block **4** ensure the stereoselective formation of 1,2-*trans* linkages, taking advantage of neighboring group participation.

**Scheme 1:**
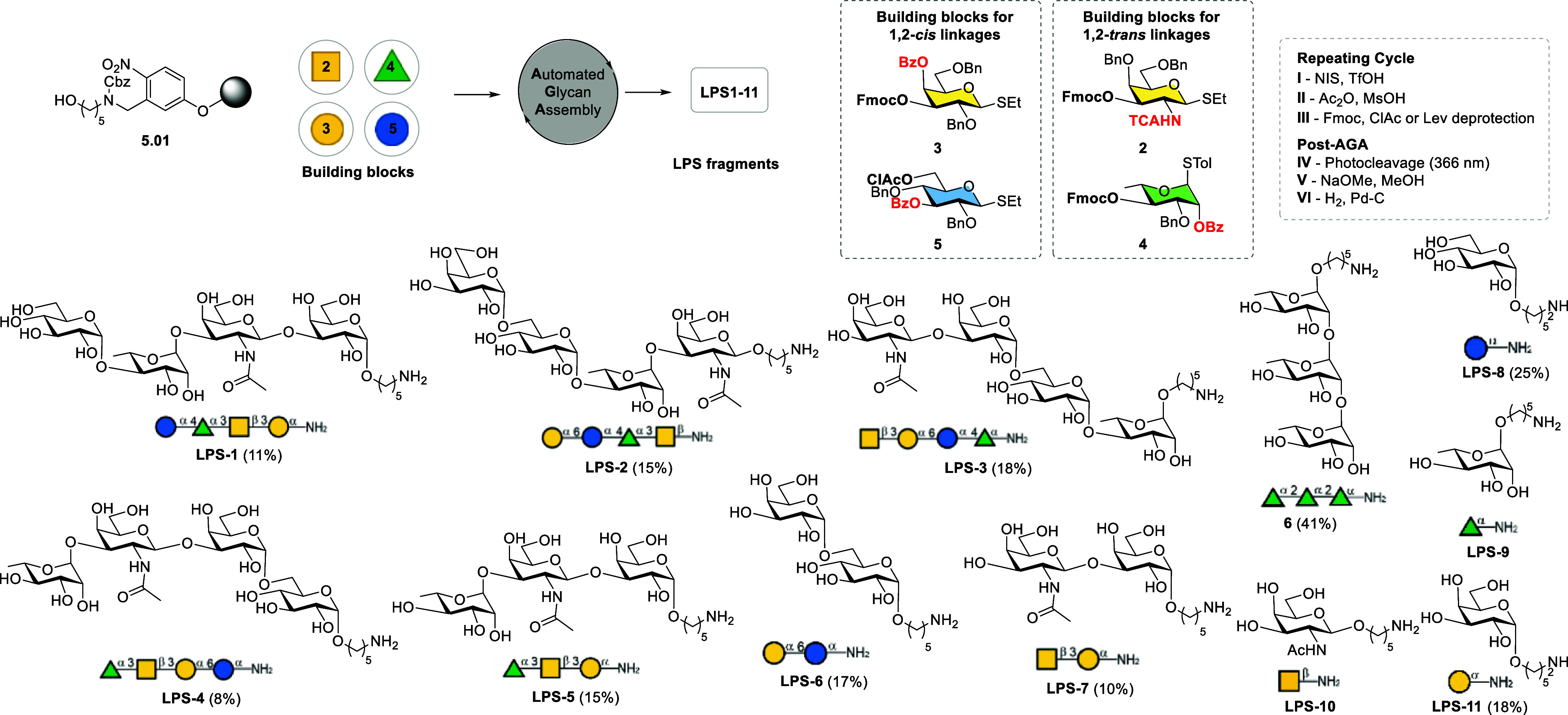
AGA of Conjugation-Ready Fragments of LPS **LPS-1** to **LPS-11** and Rhamnose Trisaccharide Control **6** Using Building Blocks **2-5** and Solid-Support **1** ^a^

a Participating groups for stereocontrol are highlighted in red. Glycosylation conditions during repeating cycle step (**I**) were optimized to the following conditions: *N-*acetylgalactosamine **2** was coupled at −40 °C for 30 min, followed by −20 °C for 30 min; galactose **3** was coupled at −40 °C for 10 min, followed by −10 °C for 50 min; rhamnose **4** was coupled at −20 °C for 10 min, followed by 0 °C for 30 min; and glucose **5** was coupled at −20 °C for 5 min, followed by 0 °C for 30 min. **LPS-9** (synthesized by Christopher E. Martin^42^) and **LPS-10** (synthesized by Claney L. Pereira^43^) were taken from the glycan database of the Max Planck Institute of Colloids and Interfaces. More detailed reaction conditions and the synthesis of building blocks **2-5** are shown in the Supporting Information.

Glucose building blocks carrying a benzoyl protecting group at C3–OH allow for the reliable construction of *cis*-glycosidic linkages.^34^ Cold-ion IR studies^36–38^ showed that high *cis*-selectivity can be achieved for galactose building blocks with an acyl group at C4–OH. Remote participation of the benzoyl group at C4–OH in galactose building block **3** and at C3–OH in glucose building block **5** was used to ensure the stereoselective formation of 1,2-*cis* linkages. Benzoyl esters were generally chosen for remote participation, as they are more readily removed during postsynthesis methanolysis, while migration is less likely compared to acetyl esters.

Using Merrifield resin equipped with photocleavable linker **1** and building blocks **2–5** in AGA, a collection of conjugation-ready fragments of the LPS (Scheme 1) were synthesized, including four tetrasaccharides **LPS-1** to **LPS-4**, as well as tri-, di-, and monosaccharides **LPS-5** to **LPS-11**.

### AGA Cycles and Post-AGA

As soon as the ideal coupling conditions of a specific building block are identified, a large number of combinatorial fragments of polysaccharides can be synthesized by using AGA.

Previous studies^39^ have shown that galactose and galactosamine building blocks, for example, exhibit very low activation temperatures and perform better at those temperatures. Galactose **3** and *N*-acetylgalactosamine **2** building blocks coupled well starting at −40 °C, while glucose **5** and rhamnose **4** required −20 °C with a longer reaction time at 0 °C. *N*-Acetylgalactosamine building block **2** proved to be very sensitive to factors such as temperature, humidity, and stability of the glycosidic bond. The change to a glycosyl phosphate did not increase the coupling efficiency, while double to triple glycosylation cycles enabled the reliable coupling of **2** up to the tetrasaccharide stage. Couplings of C3–OH at galactose and galactosamine are known to be challenging and sterically hindered by groups at the axial C4-position.^40^ Double deprotection cycles after the coupling of galactose **3** or galactoseamine **2** were required to achieve full deprotection of the Fmoc group, followed by double glycosylation cycles of the respective 1,3-coupled building block. Further deprotections (Lev at **4**, ClAc at **5**, and Fmoc at **4**), as well as capping steps, photocleavage from solid support, and global deprotection following published procedures, were performed.^41^ Employing the optimized glycosylation conditions, **LPS-1** to **LPS-11** were synthesized in common AGA-yields (8–25%).

### Stereoselectivity of α-Galactose Building Block 3 and α-Glucose Building Block 5

The TCA group at the C2-amine in GalNAc **2** and the benzoyl ester at C2–OH in rhamnose building block **4** ensured the stereoselective formation of 1,2-trans linkages, taking advantage of neighboring group participation for the stereocontrolled formation of Rha-α(1→3) and GalNAc-β(1→3) linkages. A building block^37,38^ similar to building block **3** but with a pivaloyl (Piv) group at the C4 position instead of benzoyl, was employed for the synthesis of the LPS fragments in AGA and gave excellent *cis*-stereoselectivity to soft nucleophilic glycosidic acceptors and high *cis*-stereoselectivity when coupled to the aminopentanol linker (α/β 9:1). However, post-AGA removal of the Piv group by methanolysis (NaOMe and MeOH in CH_2_Cl_2_) was sluggish. The three methyl groups at the Piv group sterically hinder the nucleophilic attack of the methoxide and cause a very slow cleavage of the Piv ester. In contrast, the benzoyl building block **3** showed excellent *cis*-stereoselectivity in glycosylations with soft glycosidic nucleophiles but lower *cis*-selectivity (α/β 4:1) when coupled to the linker. However, the efficient cleavage (2 h) of the benzoyl group by methanolysis rendered **3** the preferred choice as a building block. The stereoselective formation of 1,2-*cis* linkages in glucose was ensured by remote participation of the benzoyl ester^34^ at C3 in glucose building block **5**. Glucoside **5** gives excellent *cis*-stereoselectivity for soft nucleophilic glycosidic acceptors (α-only) as well as for the hard amino pentanol nucleophile (α/β 20:1). Generally, α-glucose **5**, as well as α-galactose building block **3**, provides excellent *cis*-stereoselectivity when used as a donor for soft glycosidic nucleophiles. Coupling building blocks **3** or **5** to the aminopentanol linker results in reduced overall yields because *cis*-stereoselectivity could not be achieved. However, the RP-HPLC retention times of the isomers directly coupled to the linker are significantly different and ensured the isolation of the α-isomer after the synthesis (Figure 2).

**Figure 2:**
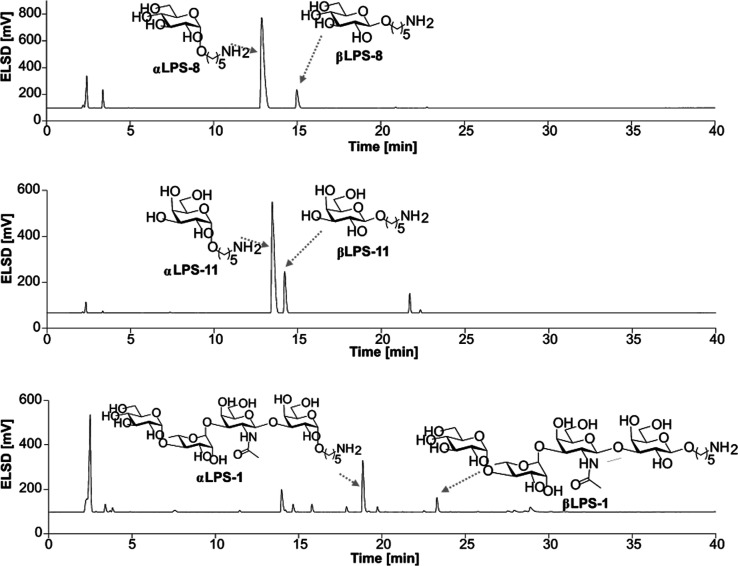
Crude RP-HPLC ELSD signals show the *cis*-stereoselectivity of building blocks **3** and **5** to the linker and sugar nucleophiles. The β-isomer, when coupled to the linker, results in significantly longer retention times, allowing for purification.

The correct stereochemistry at the anomeric center for the tetrasaccharides **LPS-1** to **LPS-4** was ascertained by coupled ^1^H,^13^C NMR spectroscopy that revealed the three α-linkages at rhamnose, glucose, and galactose (^1^J_CH_ 170 to 175) and the single β-linkage at galactosamine (^1^J_CH_ 161 to 165) (Figure 3).

**Figure 3:**
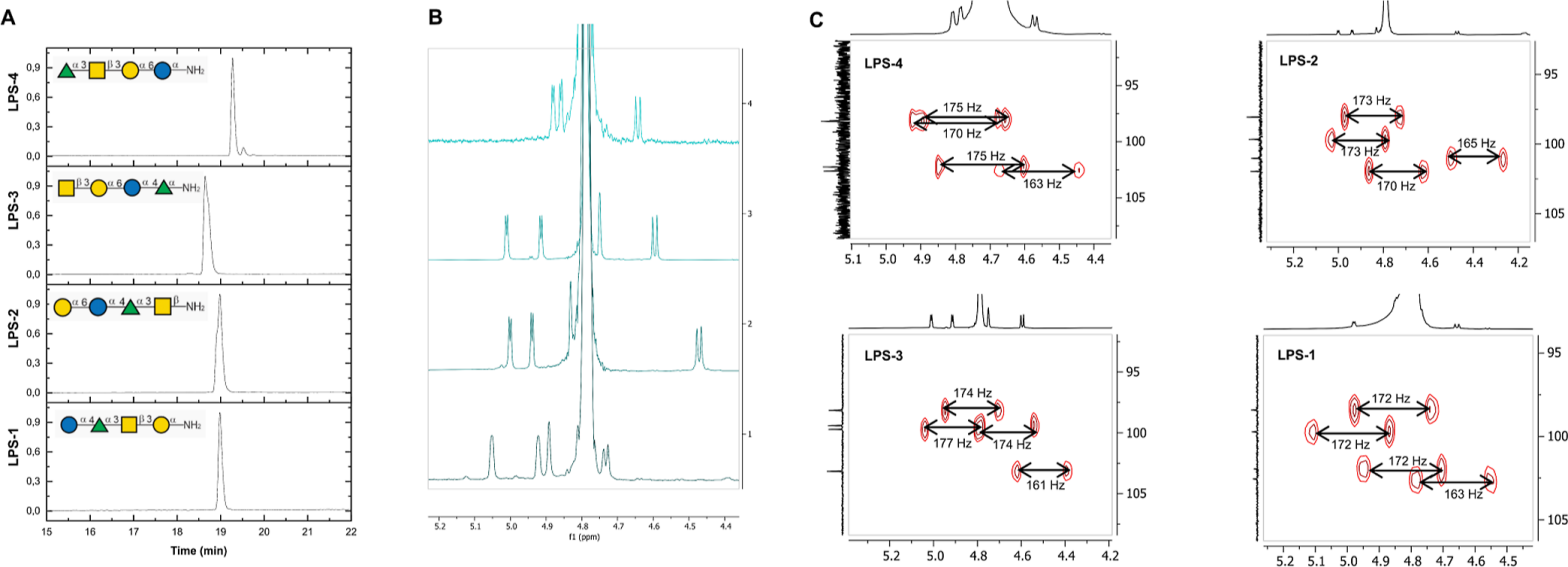
Extract from the normalized ELSD signals of the purified tetrasaccharides **LPS-1 to LPS-4** (A) and anomeric region of the ^1^H NMR spectra of the purified tetrasaccharides **LPS-1 to LPS-4** (B) showing the different retention times and chemical shifts of each glycan structure. (C) Extract from the couples ^1^H, ^13^C NMR spectra (axis in ppm) of the anomeric region of the synthesized tetrasaccharides **LPS-1-LPS-4** proving the desired stereochemistry.

### Glycan Microarray Studies

The rapid synthesis of a collection of LPS fragments by AGA was the basis for high-throughput glycan microarray studies to identify an epitope for antibody binding. Determining the epitope is essential for the design of conjugate vaccines that result in the generation of specific and protective antibodies after immunization. In previous studies,^23^ -infected patients formed IgG and IgA antibodies in blood and saliva against pathogen-specific antigens.

Here, human sera and saliva from -infected, recovered, and healthy patients were used to screen for antibodies against the synthetic glycans **LPS-1** to **LPS-11** and to determine whether LPS fragments are potential leads for vaccine development. The periodontal status of three groups of patients was defined as healthy (20 patients, no clinical indications for periodontitis^44^), inflamed (16 patients, stage III or stage IV periodontitis^45^), and stable periodontitis^44^ (6 patients). Stable periodontitis patients were treated for stage III or stage IV periodontitis by all steps of periodontal therapy, including resective and/or regenerative surgery and two to four supportive periodontal therapies per year.^46^ While was identified in biofilm samples of all patients, the bacterial amount identified by polymerase chain reaction was generally higher for patients with periodontitis (mean threshold cycle (cT) of 18.52) and stable periodontitis patients (mean­(cT) of 21.70) compared to healthy individuals (mean­(cT) of 24.71) (Table S1, Supporting Information). Synthetic conjugation-ready LPS fragments of were immobilized on NHS-activated carboxyl-functionalized glass slides in triplicate (Figure 4A,B). α(1→2)-Trirhamnose **6** served as a positive control because most humans develop antibodies against this pathogen-associated glycan.^47^

**Figure 4:**
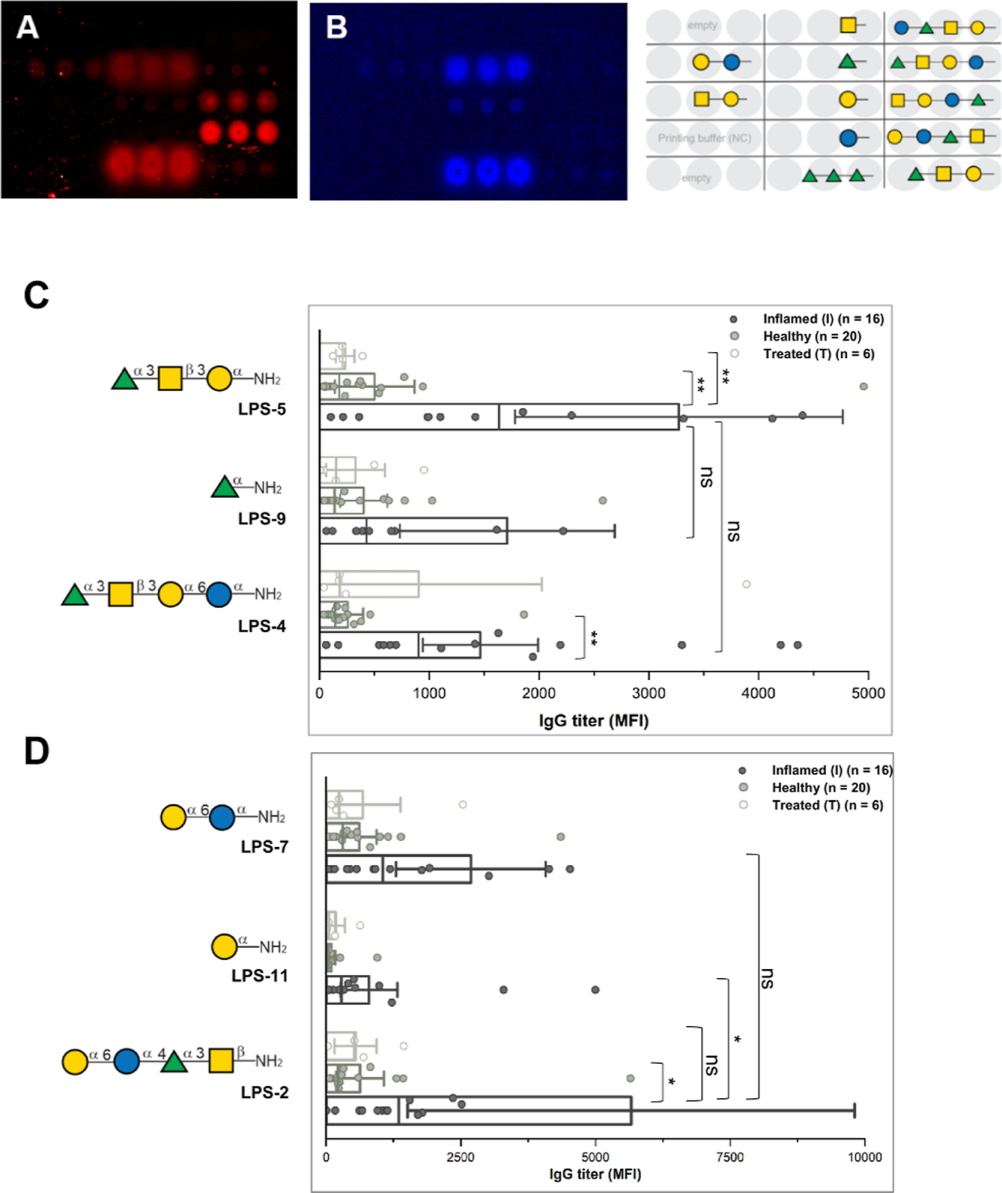
Glycan microarray studies used to determine human IgG and IgA antibodies from inflamed (I), treated (T), and healthy (H) patients binding to synthetic LPS fragments. (A) Fluorescence signals indicating IgG antibody binding in the saliva of one exemplary periodontitis patient; (B) fluorescence signals indicating IgA antibody binding in the saliva of one exemplary healthy patient. (C) Determination of mean fluorescence intensity (MFI) of human antibodies in saliva binding to terminal α(1→3)-rhamnose glycans (**P ≤ 0.01, ns—not significant, two-sided unpaired *t*-test) and (D) determination of MFI of human IgG antibodies in saliva binding to terminal α(1→6)-galactose glycans (*P ≤ 0.05, ns—not significant, two-sided unpaired *t*-test). For complete data, see Figure S1 in Supporting Information.

The glycan microarray analysis of saliva and serum generally showed IgG antibody binding to the same LPS fragments **LPS-4**, **LPS-2**, **LPS-9**, **LPS-7**, and **LPS-5**, as well as the positive control **6** (Figure 4C,D, LPS fragments without binding are displayed in the Supporting Information). Only the saliva of periodontitis patients with high bacterial load showed IgG antibody binding to the listed LPS glycan structures, while the serum of healthy and treated individuals contained IgG antibodies against those structures. Human sera contain antibodies against rhamnoses as well as α-galactoses that are common bacterial surface motifs.^48^ The specific binding of antibodies in the saliva to some LPS-glycans is evidence of active mucosal IgG antibody production during advanced infection in periodontitis patients. IgG antibodies against positive control **6** in saliva were also observed in healthy individuals but significantly less than in periodontitis and stable periodontitis patients. Generally, salivary antibodies reflect both mucosal and systemic immunity.^49,50^

The epitopes that are bound by IgG antibodies in saliva and blood are generally LPS fragments with a terminal α(1→3)-rhamnose unit (**LPS-4**, **LPS-5,** and **LPS-9**, Figure 4C) or a terminal α(1→6)-galactose unit (**LPS-2**, **LPS-7,** and **LPS-11**, Figure 4D).

A comparison of the mean fluorescence intensity (MFI) indicated IgG titers of bound human IgG antibodies in saliva compared to terminal α(1→3)-rhamnose LPS-fragments (Figure 4C). On the other hand, more IgG antibodies bind to **LPS-5** trisaccharide than to rhamnose monomer **LPS-9**, indicating that binding to **LPS-5** is not just the result of antibodies recognizing bacterial rhamnose motifs but of the actual LPS fragment of . These values, however, have low statistical significance (*P* ≤ 0.1), likely due to the different cT values within each group. Nevertheless, the statistical significance of the stronger IgG binding observed for periodontitis patients to **LPS-5** compared to low IgG binding for healthy and stable periodontitis patients is high (*P* ≤ 0.01), and there is clear evidence for the active mucosal IgG antibody production in the advanced affection of against **LPS-5** in periodontitis.

Human IgG antibodies in saliva bind with the highest MFI to the **LPS-2** tetrasaccharide (Figure 4D). Conversely, disaccharide **LPS-7** and monosaccharide **LPS-11** bind significantly less to IgG (*P* ≤ 0.05), suggesting that all four monosaccharides of the LPS repeating unit have an impact on IgG binding. Antibody binding arises not just from the binding of general α(1→6)-galactose antibodies. In addition, longer structures may be more accessible for antibody binding on the glycan array due to the distance to the glass slides and their flexibility or conformation. Furthermore, IgG binding in CP saliva to **LPS-2** is significantly higher than in periodontally healthy patients (*P* ≤ 0.05), supporting the assumption that the **LPS-2** epitope is specific for infections. The glycan microarray results show that the monosaccharide sequence in the LPS repeating unit of is important for IgG antibody binding. The terminal α(1→6)-galactose in the LPS fragment **LPS-2** is recognized by most IgG antibodies.

Binding of IgA antibodies, the predominant mucosal antibody type^51,52^ was also screened (Figure S1, Supporting Information). Saliva samples showed higher MFI compared to serum samples, as expected due to the high IgA content in saliva.^51,52^ Only rhamnose controls **LPS-9** and **6** and α-galactose dimer **LPS-7**, which are common for many bacteria are significantly bound by saliva and serum IgA.^47^ No significant difference was observed between periodontitis and stable periodontitis patients, as well as periodontally healthy individuals.

### Preparation of Glycoconjugates LPS-2-CRM_197_ and LPS-5-CRM_197_

High IgG antibody levels in the saliva and serum of CP patients against **LPS-2** were detected in glycan microarray analyses. **LPS-2** was selected among the structures with a terminal α(1→6)-galactose unit as a suitable vaccine candidate and covers the entire repeating unit. In addition, **LPS-5** was picked to investigate the influence of the terminal α(1→3)-rhamnose unit on vaccine design and showed significantly elevated levels in CP patients compared with the healthy and recovered control groups. For further immunization studies, semisynthetic glycoconjugates **LPS-2-CRM**_**197**_ and **LPS-5-CRM**_**197**_ were generated by conjugation of the synthetic glycan to the carrier protein CRM_197_ (Figure S2A). The successful conjugation was proven by MALDI-TOF-MS and SDS-PAGE (Figure S2B–F). The average loading was three **LPS-2** units and six **LPS-5** units per protein monomer.

### Immunization with Glycoconjugates

To illustrate the ability of glycoconjugates to induce a natural immune response, five mice per group were subcutaneously immunized with **LPS-2-CRM**_**197**_, **LPS-5-CRM**_**197**_, or **CRM**_**197**_. Three boosters followed the initial immunization. Since primarily affects the oral cavity, saliva samples were collected in addition to sera samples every 2 weeks (Figure 5A).

**Figure 5:**
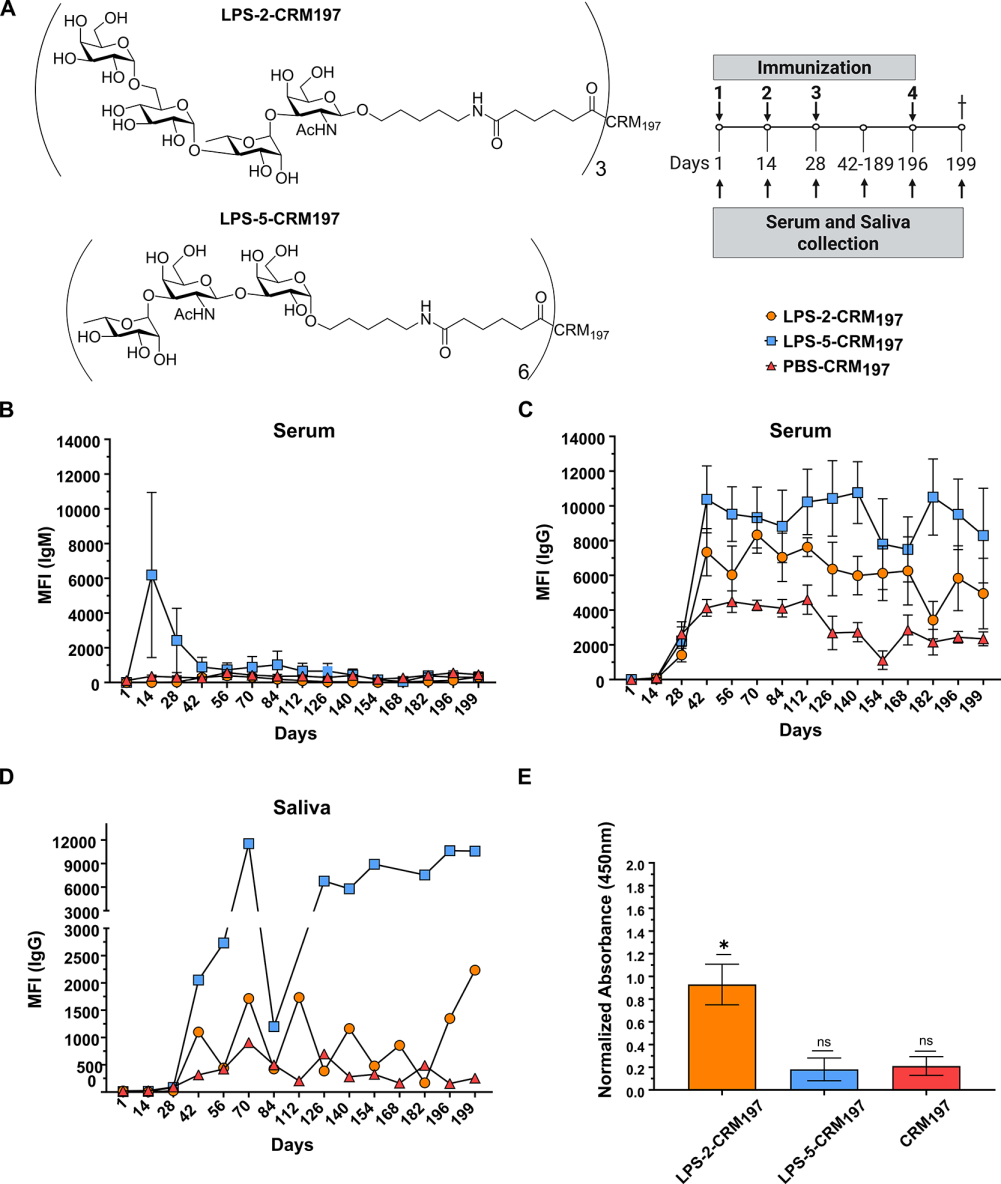
Mouse immunization schedule (A) for the active immunization with the glycoconjugate **LPS-2-CRM**_**197**_, **LPS-5-CRM**_**197**_ or PBS-CRM_197_. The primary immunization of five mice per group was followed by three boosts. Serum was collected before every immunization, every 2 weeks between day 42 and 189 and on the day of sacrifice. Glycan microarray analysis of the sera (B,C) and saliva (D) of the mice. Mean fluorescence intensity (MFI) of IgM antibodies (B) and IgG antibodies (C,D) directed toward the respective glycan or CRM_197_. Enzyme-Linked Immunosorbent Assay (ELISA) analysis of binding of IgG antibodies in pooled sera of mice immunized **with LPS-2-CRM197**, **LPS-5-CRM197**, or **PBS-CRM197** to W50 (E). Values represent mean ± SEM. Differences were tested for significance to the secondary antibody only using one sample *t*-test with *N* = 3, *: *p* < 0.0332 (E).

Glycan microarray analyses showed the development of glycan-specific IgM antibodies on day 14 in sera (Figure 5B) and IgG antibodies in sera and saliva of mice immunized with **LPS-5-CRM**_**197**_ (Figure 5C,D). Mice immunized with **LPS-2-CRM**_**197**_ showed IgG antibodies in sera and saliva that bound to **LPS-2** on the glycan microarray (Figure 5C,D). IgM antibodies remained at a low level (Figure 5B). IgG antibody levels started to increase at day 28 after initial immunization and stayed at high levels throughout the course of the experiment (Figure 5C,D). The antibody levels in mice immunized with **LPS-5-CRM**_**197**_ against **LPS-5** were in general slightly higher than the antibody levels against **LPS-2** in mice immunized with **LPS2-CRM**_**197**_ (Figure 5C,D). The higher loading of CRM_197_ with **LPS-5** than with **LPS-2** may explain this. Control mice immunized with unconjugated CRM_197_ developed only stable IgG antibody responses in sera against CRM_197_ and did not show immune responses against the synthetic structures (Figure 5C). IgM antibodies in the saliva could not be detected (Figure S2A), as well as serum and saliva IgA antibodies (Figure S2B,C). However, antibody titers may be below the detection limit since only a few microliters of saliva were recovered from each mouse.

### Bacterial ELISA with W50

A bacterial enzyme-linked immunosorbent assay (ELISA) was performed to show binding of IgG antibodies in the serum of the immunized mice to W50. Binding to the bacteria is particularly important for further vaccine development, since the epitopes can be hidden or presented differently on bacteria when compared to glycan microarrays. Mice immunized with **LPS-2-CRM**_**197**_ developed IgG antibodies in the sera binding to W50. This is consistent with the previous findings that IgG in the serum of patients infected with binds to synthetic **LPS-2.** Immunization with **LPS-5-CRM**_**197**_ or CRM_197_ did not lead to IgG antibody production that bind to the bacteria in ELISA.

## Conclusion and Outlook

 LPS oligosaccharides were prepared by AGA. α-Selective glucose and galactose building blocks helped overcome challenges in forming 1,2-*cis*-glycosidic linkages in AGA and enabled the efficient synthesis of vaccine leads against .

Glycan microarrays containing synthetic LPS fragments were used to screen for IgG and IgA in the saliva and serum of periodontitis patients, stable periodontitis patients, and periodontally healthy individuals. The α(1→6)-galactose terminal LPS-tetrasaccharide LPS-2 and the terminal α(1→3)-rhamnose LPS-trisaccharide LPS-5 were recognized in a higher level by salivary IgG from periodontitis patients compared to healthy and treated patients and nominated as the lead epitope antigens for the development of a glycoconjugate vaccine to protect from high loads. The lead epitopes **LPS-2** and **LPS-5** were conjugated to the carrier protein CRM_197_ to produce the semisynthetic glycoconjugates **LPS-2-CRM**_**197**_ and **LPS-5-CRM**_**197**_. The glycoconjugates were used to immunize mice that produced high levels of glycan-specific IgG antibodies. These antibodies bind to synthetic glycans on microarrays as well as to W50 on ELISA in the case of immunization with **LPS-2-CRM**_**197**_. Such vaccines may also be of a preventive nature when considering the negative influence of periodontitis (and ) on other noncommunicable diseases such as atherosclerosis, Alzheimer’s disease, and rheumatoid arthritis.

## Supplementary Material



## Notes

Experimental procedures and analytical data for the building block synthesis, LPS fragments, CP patient sample collection, and microarrays and further data on the microarray results (PDF)
