# Supplementary material for: Automated Glycan Assembly of Lipopolysaccharide Epitopes for Vaccine Design
Source: J Am Chem Soc. 2025 Jul 10;147(29):25969–77. doi: 10.1021/jacs.5c08663 (PMC12291450; doi:10.1021/jacs.5c08663)
Supplement: Supplementary file 1 [file ja5c08663_si_001.pdf]

Supporting Information

Automated Glycan Assembly of *Porphyromonas Gingivalis* Lipopolysaccharide Epitopes for Vaccine Design

Sabrina Omoregbee-Leichnitz[a],[b], Emelie E. Reuber[a],[b], Fabienne Weber[a],[b], Kim N. Stolte[c], José Danglad-Flores[b], Henrik Dommisch[c], Peter H. Seeberger[a],[b]*

[a] Freie Universität Berlin, Institute of Chemistry and Biochemistry, 14195 Berlin, Germany

[b] Max Planck Institute of Colloids and Interfaces, 14476 Potsdam, Germany

[c] Department of Periodontology, Oral Medicine and Oral Surgery, Institute for Dental and Craniofacial Sciences, Charité - Universitätsmedizin Berlin, corporate member of Freie Universität Berlin, Humboldt-Universität zu Berlin, and Berlin Institute of Health, 14197 Berlin, Germany

* Corresponding author: Peter H. Seeberger. Email: peter.seeberger@mpikg.mpg.de

Table of Contents

1 General Information 4

2 Materials and Conditions for Automated Synthesis 4

2.1 Materials and Measurements 4

2.2 Preparation of Stock Solutions 5

2.3 Modules for Automated Synthesis 5

2.4 Post-automated Synthesis Manipulations, Analysis and Purification 7

Analytical NP/RP-HPLC and purification 8

3 Automated Glycan Assembly of the Glycan Targets 9

5-Amino-pentyl α-D-glucopyranosyl-(1→4)-α-L-rhamnopyranosyl-(1→3)-2-acetamido-2-deoxy-β-D-galactopyranosyl-(1→3)-α-D-galactopyranoside (LPS-1) 9

5-Amino-pentyl α-D-galactopyranosyl-(1→6)-α-D-glucopyranosyl-(1→4)-α-L-rhamnopyranosyl-(1→3)-2-acetamido-2-deoxy-β-D-galactopyranoside (LPS-2) 13

5-Amino-pentyl 2-acetamido-2-deoxy-β-D-galactopyranosyl-(1→3)-α-D-galactopyranosyl-(1→6)-α-D-glucopyranosyl-(1→4)-α-L-rhamnopyranoside (LPS-3) 16

5-Amino-pentyl α-L-rhamnopyranosyl-(1→3)-2-acetamido-2-deoxy-β-D-galactopyranosyl-(1→3)-α-D-galactopyranosyl-(1→6)-α-D-glucopyranoside (LPS-4) 20

5-Amino-pentyl α-D-galactopyranoside (LPS-11) 24

5-Amino-pentyl α-D-glucopyranoside (LPS-8) 27

5-Amino-pentyl 2-acetamido-2-deoxy-β-D-galactopyranosyl-(1→3)-α-D-galactopyranoside (LPS-6) 30

5-Amino-pentyl α-L-rhamnopyranosyl-(1→3)-2-acetamido-2-deoxy-β-D-galactopyranosyl-(1→3)-α-D-galactopyranoside (LPS-5) 34

5-Amino-pentyl β-D-galactopyranosyl-(1→3)-α-D-galactopyranosyl-(1→6)-α-D-glucopyranoside (LPS-7) 38

5-Amino-pentyl α-(1→2)-L-trirhamnopyranoside (6) 41

4 Preparation of Building Blocks 43

4.1 Rhamnose 43

4-Methylphenyl 2,3-*O*-(1-methylethylidene)-4-*O*-(2-naphthalenylmethyl)-1-thio-α-L-rhamnopyranoside (5.12) 43

4-Methylphenyl 4-*O*-(2-naphthalenylmethyl)-1-thio-α-L-rhamnopyranoside (5.13) 46

4-Methylphenyl 3-*O*-benzyl-4-*O*-(2-naphthalenylmethyl)-1-thio-α-L-rhamnopyranoside (5.14) 48

4-Methylphenyl 3-*O*-benzyl-2-*O*-levulinyl-4-*O*-fluorenylmethoxycarbonyl-1-thio-α-L-rhamnopyranoside (5.15a) 51

4-Methylphenyl 2-*O*-benzoyl 3-*O*-benzyl-4-*O*-fluorenylmethoxycarbonyl-1-thio-α-L-rhamnopyranoside (4) 54

4.2 Galactose 58

Ethyl 2-*O*-benzyl-3-*O*-(2-naphthalenylmethyl)-4,6-*O*-[(*S*)-phenylmethylene]-1-thio-β-D-galactopyranoside[4] (5.08) 58

Ethyl 4-*O*-benzoyl-2,6-bis-*O*-benzyl-3-*O*-(2-naphthalenylmethyl)-1-thio-β-D-galactopyranoside (5.09) 61

Ethyl 4-*O*-benzoyl-2,6-bis-*O*-benzyl-3-*O*-fluorenylmethoxycarbonyl-1-thio-β-D-galactopyranoside (3) 64

4.3 Glucose 67

Ethyl 2-*O*-benzyl-4,6-*O*-[(*S*)-phenylmethylene]-1-thio-β-D-glucopyranoside[5] (5.16a) 67

Ethyl 3-*O*-benzoyl-2,4-bis-*O*-benzyl-1-thio-β-D-glucopyranoside (5.18) 68

Ethyl 3-*O*-benzoyl-2,4-bis-*O*-benzyl-6-*O*-(2-chloroacetyl)-1-thio-β-D-glucopyranoside (5) 71

4.4 Galactosamine 73

5 Human Sera and Saliva Sample Collection and Characterization 73

5.1 General Information 73

5.2 Sera Collection 73

5.3 Saliva Collection 74

5.4 PCR analysis of *Porphyromonas gingivalis* in dental plaque biofilm 74

6 Glycan Microarrays 75

6.1 Printing of Microarray-Slides 75

6.2 Glycan Microarray Experiments 75

6.3 Statistical Analysis 76

6.4 Further Microarray Data 77

6.5 *In vivo* and *in vitro* testing of vaccine candidates LPS-2-CRM_{197} and LPS-5-CRM_{197} 78

1 General Information

All **chemicals** were reagent grade and used as supplied unless otherwise noted. All **solvents** for chemical reactions were commercially purchased in p.a. quality. If stated, they were dried in a Solvent Dispensing System (J.C. Meyer). For HPLC and MS spectrometry, solvents with corresponding quality were used. Water was used from a Milli Q-station from Millipore. **The automated syntheses** were performed on a home-built synthesizer and the prototype 3.0 developed at the Max Planck Institute of Colloids and Interfaces.

Reaction completion, identity, and purity of all compounds were determined by low resolution mass spectrometry (**ESI-LRMS**) or analytical thin-layer chromatography (**TLC**). TLC was performed on Merck silica gel 60 F_{254} plates (0.25 mm). Compounds were visualized by UV irradiation (254 nm) or stained (5% sulfuric acid in ethanol or Hanessian's Stain: 235 mL of distilled water, 12 g of ammonium molybdate, 0.5 g of ceric ammonium molybdate, and 15 mL sulfuric acid). **Flash column chromatography** was performed on Kieselgel 60 with 230-400 mesh (Sigma-Aldrich, St. Louis, USA). Analysis and purification by normal and reverse phase **HPLC** and ESI-LRMS was performed by using an Agilent 1200 series. ^{1}H, ^{13}C, COSY and HSQC **NMR spectra** were recorded in parts per million (δ) relative to the resonance of the solvent on a Varian 400-MR (400 MHz), Varian 600-MR (600 MHz), or Bruker Biospin AVANCE700 (700 MHz) spectrometer. Assignments were supported by COSY and HSQC experiments. High resolution mass spectra (**HRMS**) were obtained using 6210 ESI-TOF mass spectrometer (Agilent) and **MALDI-TOF** autoflexTM (Bruker) instruments.

2 Materials and Conditions for Automated Synthesis

2.1 Materials and Measurements

Solvents used for dissolving all building blocks and making of various solutions were taken from Solvent Dispensing System (J.C. Meyer). Wash solvents were HPLC grade. Prior to automated synthesis, the building blocks were weighed and co-evaporated three times with anhydrous toluene and dried for at least one hour under high vacuum prior to use. All solutions were freshly prepared in oven-dried, argon-flushed glassware and kept under argon during the automation process. Isolated product yields were calculated on the basis of resin loading. Functionalized resin **1** was synthesized as previously reported

and resin loading (0.40 mmol/g) was determined following a published protocol.[2] Resin was placed in the reaction vessel and was swollen in dichloromethane for 20 min at room temperature before starting the first module. During this time, all reagent lines involved in the synthesis were washed and primed.

2.2 Preparation of Stock Solutions

Building Block Solution: Thioglycoside building block was dissolved in 1 mL (per cycle) of anhydrous CH_2Cl_2.

Activator Solution/Acidic Wash Solution: TMSOTf (0.9 mL, 0.62 mmol) was added to 80 mL of anhydrous CH_2Cl_2.

Pre-Capping Solution: Pyridine (10 mL) was added to 90 mL of DMF.

Capping Solution: Methanesulfonic acid (1.2 mL, 18.5 mmol), acetic anhydride (6 mL, 63.5 mmol) were added to 50 mL of anhydrous CH_2Cl_2.

Lev Deprotection Solution: $N_2H_4 \cdot HOAc$ (725 mg, 7.87 mmol) was dissolved in 50 mL of a 4:1:0.25 mixture of pyridine/acetic acid/water.

Fmoc Deprotection Solution 1: Piperidine (20 mL) was added to 80 mL anhydrous DMF.

Fmoc Deprotection Solution 2: Et_3N (20 mL) was added to 80 mL anhydrous DMF.

ClAc Deprotection Solution: Thiourea (2.5 g, 32.84 mmol) was dissolved in 55 mL of a 10:1 mixture of 2-methoxyethanol/pyridine.

2.3 Modules for Automated Synthesis

Initiation: The resin loaded in the reaction vessel is washed with DMF, THF, and CH_2Cl_2 (3 x 3 mL for 15 s, respectively). The resin is then swollen in 2 mL CH_2Cl_2 for 20 minutes while the temperature of the reaction vessel is cooled to the lowest temperature required throughout the synthesis.

Module I - Acidic Washing: Once the temperature of the reaction vessel has adjusted to the desired temperature of the subsequent glycosylation by the cooling device, 1 mL of the **Acidic Wash Solution** is delivered to the reaction vessel through the precooling device (set at -20 °C). After three minutes, the solution is drained. Finally, the resin is washed with 3 mL CH_2Cl_2 (bubbling = 15 s) and drained.

Module IIa – Glycosylation (for thioglycosides): Upon draining the CH_2Cl_2 in the reaction vessel, 1 mL of **Building Block Solution 1** containing the appropriate building block is delivered from the building block storing component to the reaction vessel. After the temperature reaches the desired temperature (T_1), **Activator Solution 1** (1 mL) is delivered to the reaction vessel from the respective activator storing component to the reaction vessel The glycosylation mixture is incubated for the selected duration (t_1) at the desired T_1, the reaction temperature is linearly ramped to T_2 (rate = 4 °C/min). Once T_2 is reached, it is maintained and the reaction mixture is incubated for an additional time (t_2). Once the incubation

time is finished, the reaction mixture is drained and the resin is washed with CH_2Cl_2 (1 x 2 mL for 15 s), then dioxane (1 x 2 mL for 15 s), and finally CH_2Cl_2 (2 x 2 mL for 15 s).

Module IIIa - Capping: The resin is washed with DMF (2 x 3 mL for 15 s). Then **Pre-capping Solution** (2 mL) is delivered and the reaction temperature is adjusted to and maintained at 50 °C for one minute (max power = 5 W). The resin is then washed with CH_2Cl_2 (3 x 2 mL for 15). Upon washing, **Capping Solution** (4 mL) is delivered and the temperature is adjusted and maintained 25 °C). The resin and the reagents are incubated for 8 min. The solution is then drained from the reactor vessel and the resin is washed with CH_2Cl_2 (3 x 3 mL for 15 s).

Module IVa - Fmoc Deprotection 1: The resin is first washed with DMF (3 x 3 mL for 15 s), and then **Fmoc Deprotection Solution 1** (2 mL) is delivered to the reaction vessel. The temperature of the reagents inside the reactor vessel is then adjusted to and maintained at 60 °C. After 1 min the reaction solution is drained and the resin is washed with DMF (3 x 3 mL for 15 s) and CH_2Cl_2 (5 x 3 mL for 15 s). After this module the resin is ready for the next glycosylation cycle.

Module IVb - Lev Deprotection: The resin is washed with CH_2Cl_2 (3 x 2 mL for 15 s), and then **Lev Deprotection Solution** (2 mL) is delivered to the reaction vessel. The temperature of the reagents inside the reactor vessel is then adjusted to and maintained at 25 °C. After 5 min, the reaction solution is drained from the reactor vessel and the resin is washed with CH_2Cl_2 (3 x 2 mL for 15 s). Then, of fresh **Lev Deprotection Solution** (2 mL) is delivered and the process is repeated twice more. Then, the resin is washed with DMF, THF, and CH_2Cl_2 (3 x 3 mL for 15 s, respectively). After this module the resin is ready for the next glycosylation cycle.

Module IVc - Fmoc Deprotection 2: The resin is first washed with DMF (3 x 3 mL for 15 s), and then **Fmoc Deprotection Solution 2** (2 mL) is delivered to the reaction vessel. The temperature of the reagents inside the reactor vessel is then adjusted to and maintained at 60 °C. After 5 min the reaction solution is drained and the resin is washed with DMF (3 x 2 mL for 15 s). Then, fresh **Fmoc Deprotection Solution 2** (2 mL) is delivered and the process is repeated twice more. Then, the resin is washed with DMF (3 x 3 mL) and CH_2Cl_2 (3 x 3 mL) for 15 s each time. After this module the resin is ready for the next glycosylation cycle.

Module IVe – ClAc Deprotection: The resin is first washed with CH_2Cl_2 (3 x 2 mL for 15 s) then **ClAc Deprotection Solution** (2 mL) was delivered to the reaction vessel. The temperature of the reagents inside the reactor vessel is then adjusted to and maintained at 90 °C. After 22 min, the reaction solution is drained from the reactor vessel. The resin is washed with DMF (3 x 2 mL for 15 s). Then fresh **ClAc Deprotection Solution 2** (2 mL) is delivered and the process is repeated twice more. Then, the resin is washed with DMF (3 x

3 mL for 15 s) and CH_2Cl_2 (5 x 3 mL for 15 s). After this module the resin is ready for the next glycosylation cycle.

2.4 Post-automated Synthesis Manipulations, Analysis and Purification

Cleavage from Solid Support (Method A-1): Protected Oligosaccharides

After automated synthesis, the resin was removed from the reaction vessel, suspended in CH_2Cl_2 (20 mL), and photocleaved in a continuous-flow photoreactor. A Vapourtec E-Series easy-MedCHem, equipped with a UV-150 Photochemical reactor having a UV-150 Medium-Pressure Mercury Lamp (arc length 27.9 cm, 450 W) surrounded by a long-pass UV filter (Pyrex, 50% transmittance at 305 nm) was used. A Pump 11 Elite Series (Harvard Apparatus syringe pump at a flow rate of 0.8 mL/min was used to pump the mixture through a FEP tubing (i.d. 3.0 inch, volume: 12 mL) at 20 °C. The reactor was washed with 20 mL CH_2Cl_2 at a flow rate of 2.0 mL/min. The output solution was filtered to remove the resin and the solvent was evaporated *in vacuo*. Crude was then analyzed by MALDI.

Deprotection of Oligosaccharides

AGA-synthesized and photocleaved product was subjected to methanolysis and hydrogenolysis. The hydrogenolysis product was purified by RP-HPLC and lyophilized on a Christ Alpha 2-4 LD plus freeze dryer to afford the final deprotected compound.

- **Methanolysis (Method C):** To a solution of protected oligosaccharide in MeOH:CH_2Cl_2 (2 mL, 1:1), sodium methoxide (0.5 M solution in MeOH, 2.2 equiv. per ester group) was added. The mixture was stirred at room temperature for 2 h. Then Amberlite IR-120 (H^+ form) was added to quench. After neutralization, the reaction mixture was filtered and the solvent was removed in vacuo. The crude compound was used for hydrogenolysis without further purification.
- **Hydrogenolysis (Method D):** The crude compound obtained after methanolysis was dissolved in 4 mL of EtOAc:t-BuOH:H_2O (2:1:1). Pd/C (10%) was added to the solution and the suspension was stirred in a H_2 bomb with 60 psi pressure over night. The insoluble material was removed by a CHROMAFIL ®Xtra, RC 0.45 syringe filter. The solid was washed once with t-BuOH and several times with water. The filtrate was collected and concentrated in vacuo.

Analytical NP/RP-HPLC and purification

Analytical NP-HPLC of Crude Material (Method B-2)

Analytical NP-HPLC was conducted on an Agilent 1200 Series system. A YMC-Diol-300-NP column (150 mm x 4.60 mm I.D.) was used at a flow rate of 1.00 mL/min with hexane/EtOAc as eluent (20% EtOAc in hexane for 5 min, 20 → 55% EtOAc in hexane over 35 min, 55 → 100% EtOAc in hexane over 35 min, 100% EtOAc for 10 min).

Analytical/preparative RP-HPLC of Crude Material (Method E-1)

Crude products were dissolved in water and analyzed/purified using analytical/preparative HPLC. A Thermo-Scientific Hypercarb column (150 mm x 4.60 mm I.D.) was used for analytical RP-HPLC with a flow rate of 0.70 mL/min with water (0.1% HCO_2H)/acetonitrile as eluents (100% H_2O (0.1% HCO_2H) for 5 min, 0 → 30% acetonitrile in H_2O (0.1% HCO_2H) over 30 min, 30 → 100% acetonitrile in H_2O (0.1% HCO_2H) over 5 min, 100% acetonitrile for 5 min).

3 Automated Glycan Assembly of the Glycan Targets

5-Amino-pentyl α-D-glucopyranosyl-(1→4)-α-L-rhamnopyranosyl-(1→3)-2-acetamido-2-deoxy-β-D-galactopyranosyl-(1→3)-α-D-galactopyranoside (LPS-1)

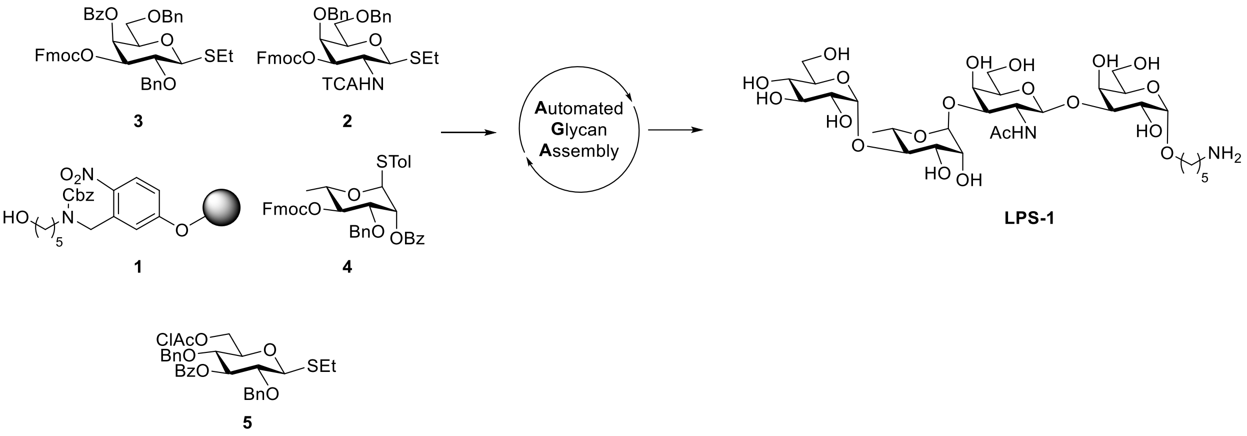

Repeat	Building Blocks	Modules	Notes
1x		**I** – Acidic Wash	
	**3** (2 x 5.0 equiv.)	**IIa** – Glycosylation with thioglycoside – 2 cycles	-40 °C (T_1) 10 min (t_1)<br>-10 °C (T_2) 50 min (t_2)
		**III** – Capping<br>**IVa** – Fmoc Deprotection - 2 cycles	
1x		**I** – Acidic Wash	
	**2** (2 x 5.0 equiv.)	**IIa** – Glycosylation with thioglycoside – 2 cycles	-40 °C (T_1) 30 min (t_1)<br>-20 °C (T_2) 20 min (t_2)
		**III** – Capping<br>**IVa** – Fmoc Deprotection - 2 cycles	
1x		**I** – Acidic Wash	
	**4** (2 x 5.0 equiv.)	**IIa** – Glycosylation with thioglycoside – 2 cycles	-20 °C (T_1) 10 min (t_1)<br>0 °C (T_2) 30 min (t_2)
		**III** – Capping<br>**IVa** – Fmoc Deprotection	
1x		**I** – Acidic Wash	
	**5** (2 x 5.0 equiv.)	**IIa** – Glycosylation with thioglycoside – 2 cycles	-20 °C (T_1) 5 min (t_1)<br>0 °C (T_2) 30 min (t_2)

Protected **LPS-1** (22 mg, 0.011 mmol, crude yield: 81%) was obtained as a colorless oil after photocleavage from solid support following **Method A-1**. Deprotection of **LPS-1** following **Method C** and **D** and purification by reverse-phase HPLC (**Method E-1**, t_R = 18.8 min) afforded deprotected compound **LPS-1** (1.1 mg, 0.002 mmol, 11%) as a white solid after lyophylization.

^{1}H NMR (700 MHz, D_2O): δ 4.97 – 4.92 (m, 1H), 4.85 – 4.80 (m, 1H), 4.79 (s, 1H), 4.63 (d, J = 8.2 Hz, 1H), 4.10 (s, 1H), 4.01 – 3.55 (m, 19H), 3.49 – 3.42 (m, 2H), 3.43 – 3.35 (m, 2H), 2.94 – 2.89 (m, 2H), 1.95 (s, 3H), 1.64 – 1.57 (m, 4H), 1.40 – 1.34 (m, 2H), 1.27 (d, J = 6.2 Hz, 3H) ppm.

^{13}C NMR (176 MHz, D_2O): δ 174.9, 102.5, 102.0, 99.7, 98.4, 81.0, 79.0, 75.0, 72.7, 70.7, 70.5, 69.3, 68.7, 68.4, 67.8, 67.3, 61.2, 60.9, 60.1, 51.8, 39.4, 28.1, 26.6, 22.4, 22.2, 16.9 ppm.

HRMS (QToF): Calcd for $C_{31}H_{57}N_2O_{20}$ $[M + H]^+$ 777.3499; found 777.3508.

RP-HPLC of crude deprotected **LPS-1** (ELSD trace, **Method E-1**, t_R = 18.8 min):

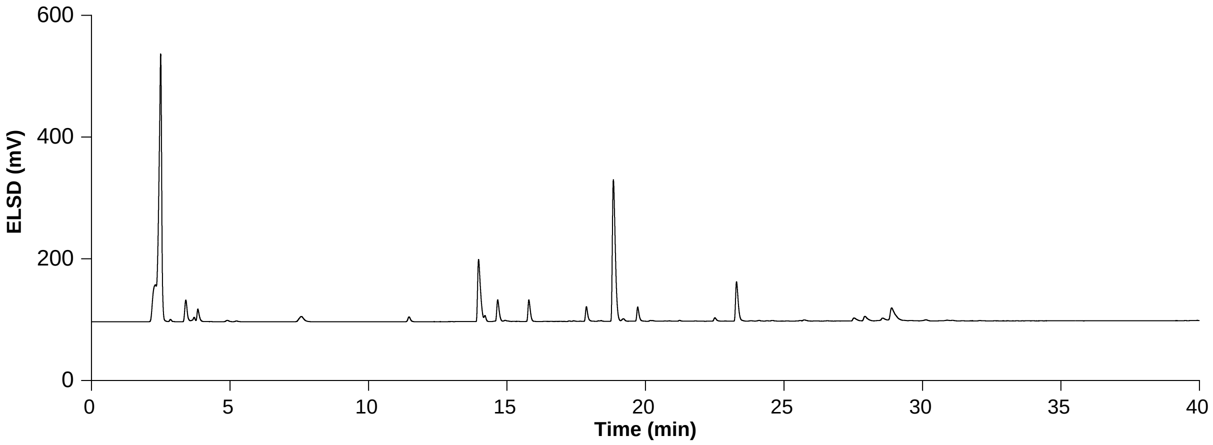

RP-HPLC of purified deprotected **LPS-1** (ELSD trace, **Method E-1**, t_R = 18.8 min):

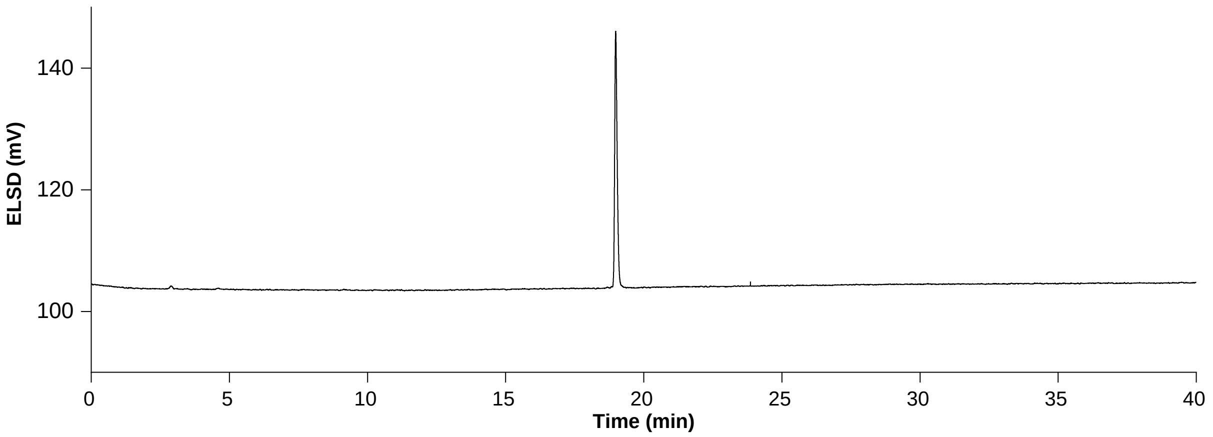

^{1}H NMR (700 MHz, D_2O) of **LPS-1**:

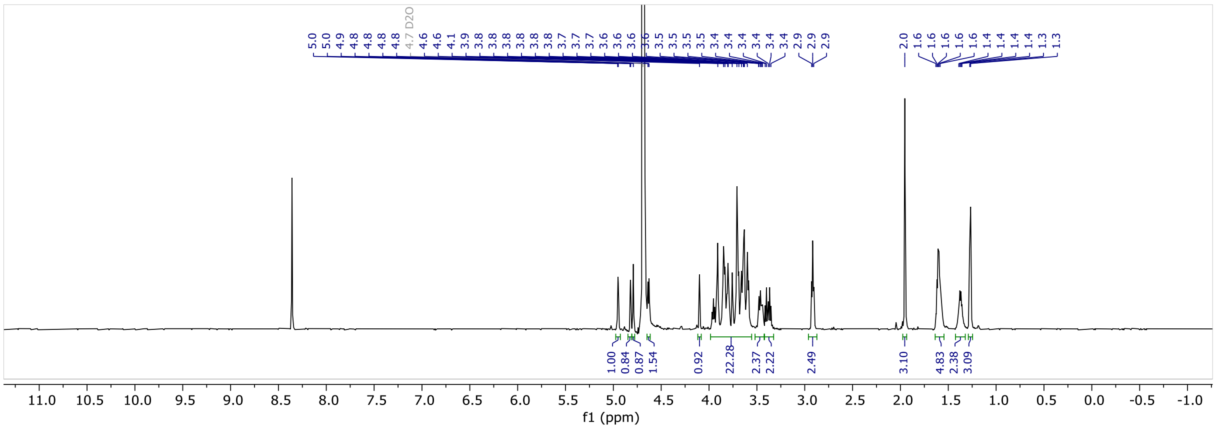

^{13}C NMR (176 MHz, D_2O) of **LPS-1**:

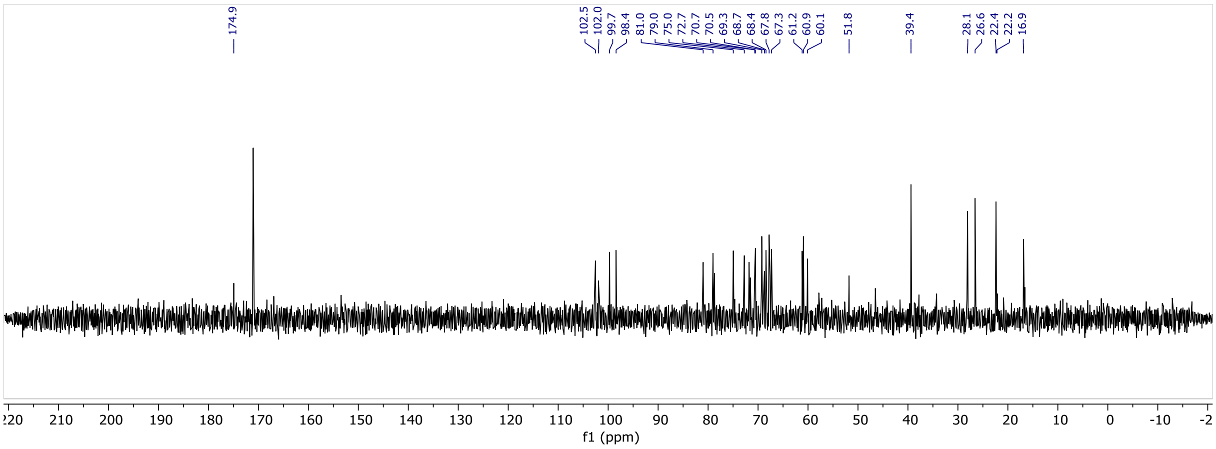

Coupled ^{13}C,^{1}H HSQC of **LPS-1**:

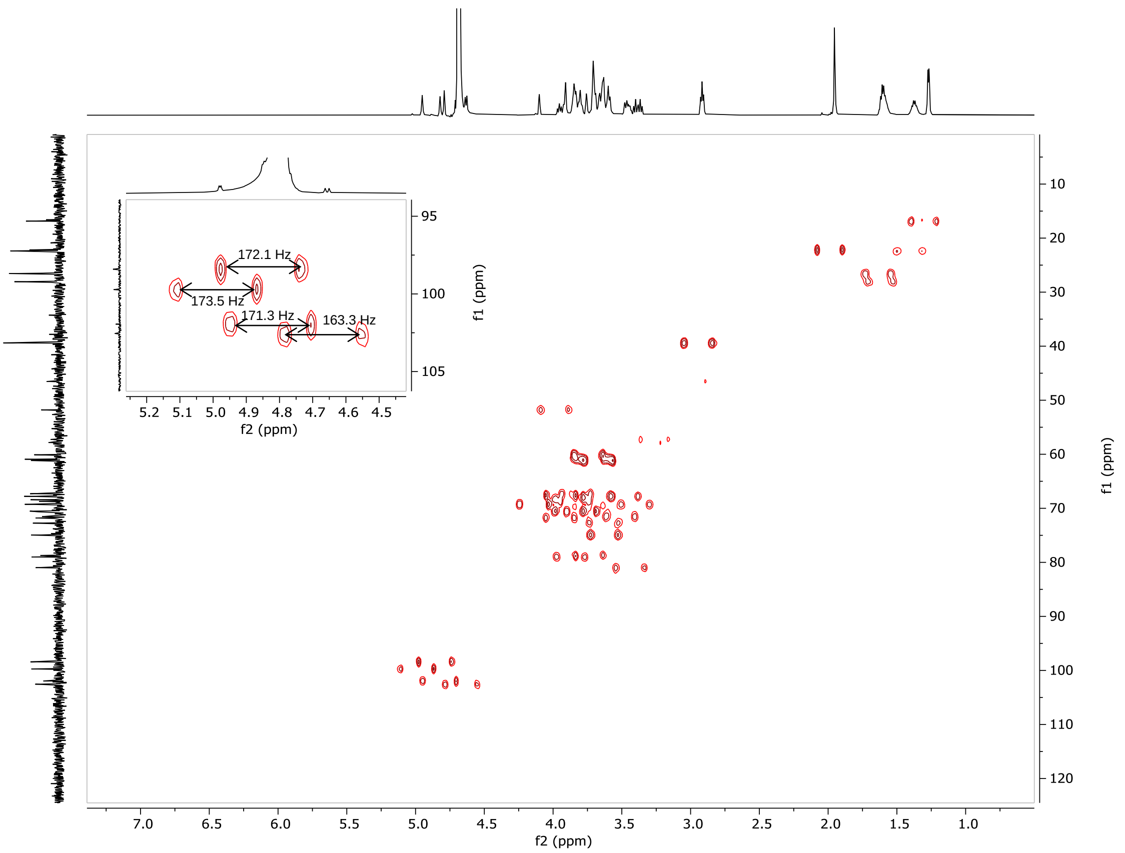

^{13}C,^{1}H HSQC of **LPS-1**:

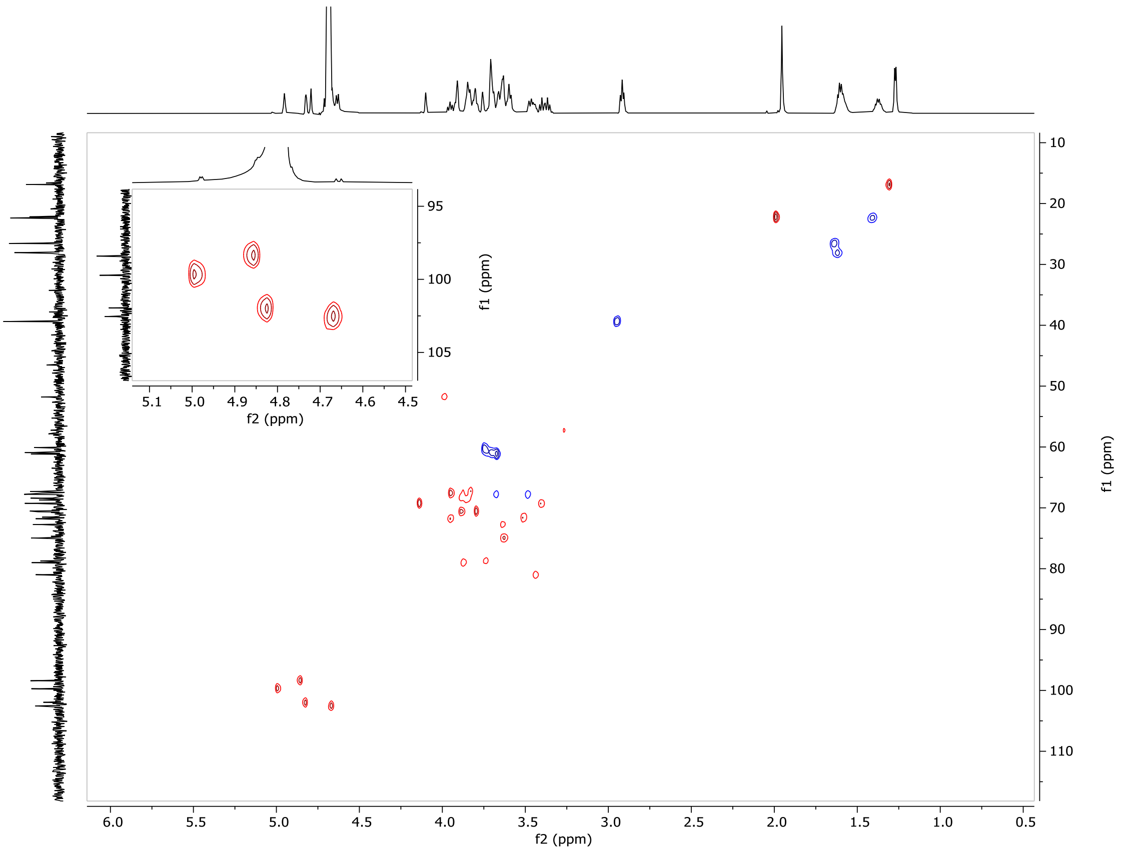

5-Amino-pentyl α-D-galactopyranosyl-(1→6)-α-D-glucopyranosyl-(1→4)-α-L-rhamnopyranosyl-(1→3)-2-acetamido-2-deoxy-β-D-galactopyranoside (LPS-2)

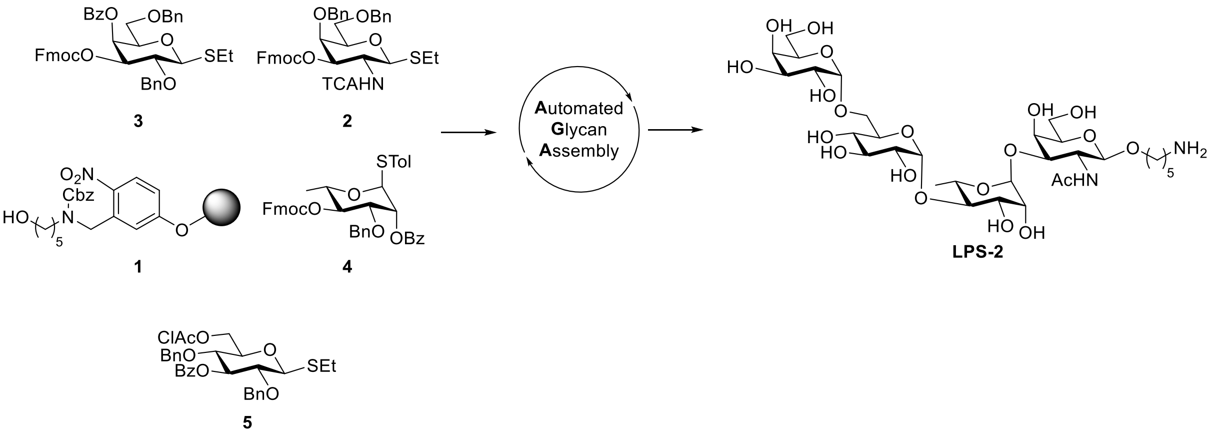

Repeat	Building Blocks	Modules	Notes	
1x		**I** – Acidic Wash		
	**2** (2 x 5.0 equiv.)	**IIa** – Glycosylation with thioglycoside – 2 cycles	-40 °C (T_1)<br>-20 °C (T_2)	30 min (t_1)<br>20 min (t_2)
		**III** – Capping<br>**IVc** – Fmoc Deprotection - 2 cycles		
1x		**I** – Acidic Wash		
	**4** (2 x 5.0 equiv.)	**IIa** – Glycosylation with thioglycoside – 2 cycles	-20 °C (T_1)<br>0 °C (T_2)	10 min (t_1)<br>30 min (t_2)
		**III** – Capping<br>**IVc** – Fmoc Deprotection		
1x		**I** – Acidic Wash		
	**5** (2 x 5.0 equiv.)	**IIa** – Glycosylation with thioglycoside – 2 cycles	-20 °C (T_1)<br>0 °C (T_2)	5 min (t_1)<br>30 min (t_2)
		**III** – Capping<br>**IVe** – ClAc Deprotection		
1x		**I** – Acidic Wash		
	**3** (2 x 5.0 equiv.)	**IIa** – Glycosylation with thioglycoside – 2 cycles	-40 °C (T_1)<br>-10 °C (T_2)	10 min (t_1)<br>50 min (t_2)

Protected **LPS-2** (26 mg, 0.012 mmol, crude yield: 86%) was obtained as a colorless oil after photocleavage from solid support following **Method A-1**. Deprotection of **LPS-2** following **Method C** and **D** and purification by reverse-phase HPLC (**Method E-1**, t_R = 18.9 min) afforded deprotected compound **LPS-2** (1.5 mg, 0.002 mmol, 15%) as a white solid after lyophylization.

^{1}H NMR (700 MHz, D_2O): δ 5.00 (d, J = 4.0 Hz, 1H), 4.94 (d, J = 3.8 Hz, 1H), 4.83 (s, 1H), 4.47 (d, J = 8.6 Hz, 1H), 4.19 – 4.15 (m, 1H), 3.99 – 3.40 (m, 23H), 2.97 – 2.92 (m, 2H), 2.00

(s, 3H), 1.67 – 1.60 (m, 2H), 1.59 – 1.54 (m, 2H), 1.40 – 1.33 (m, 2H), 1.31 (d, J = 6.3 Hz, 3H) ppm.

^{13}C NMR (176 MHz, D_2O): δ 174.4, 102.0, 101.0, 99.7, 98.1, 80.9, 78.9, 75.0, 72.8, 71.4, 70.9, 70.6, 70.4, 69.9, 69.4, 69.1, 68.7, 68.4, 68.3, 67.5, 65.4, 61.0, 60.8, 51.4, 39.2, 28.0, 26.3, 22.1, 22.0, 16.7 ppm.

HRMS (QToF): Calcd for $C_{31}H_{57}N_2O_{20}$ $[M + H]^+$ 777.3499; found 777.3489.

RP-HPLC of purified deprotected **LPS-2** (ELSD trace, **Method E-1**, t_R = 18.9 min):

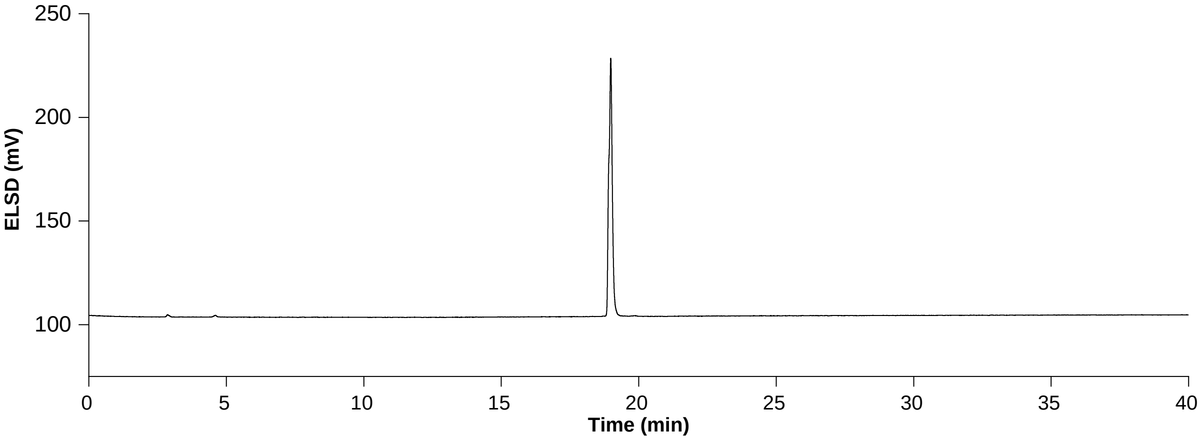

^{1}H NMR (700 MHz, D_2O) of **LPS-2**:

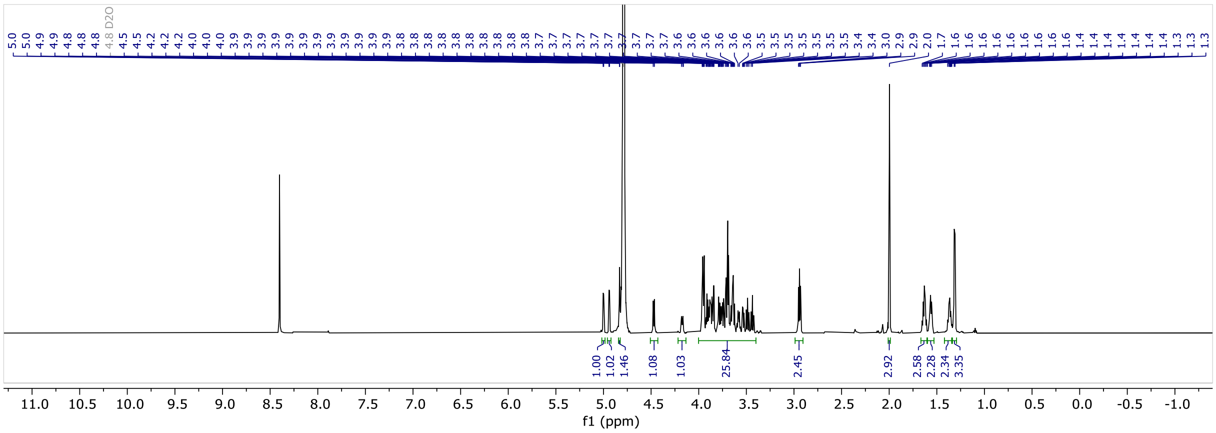

^{13}C NMR (176 MHz, D_2O) of **LPS-2**:

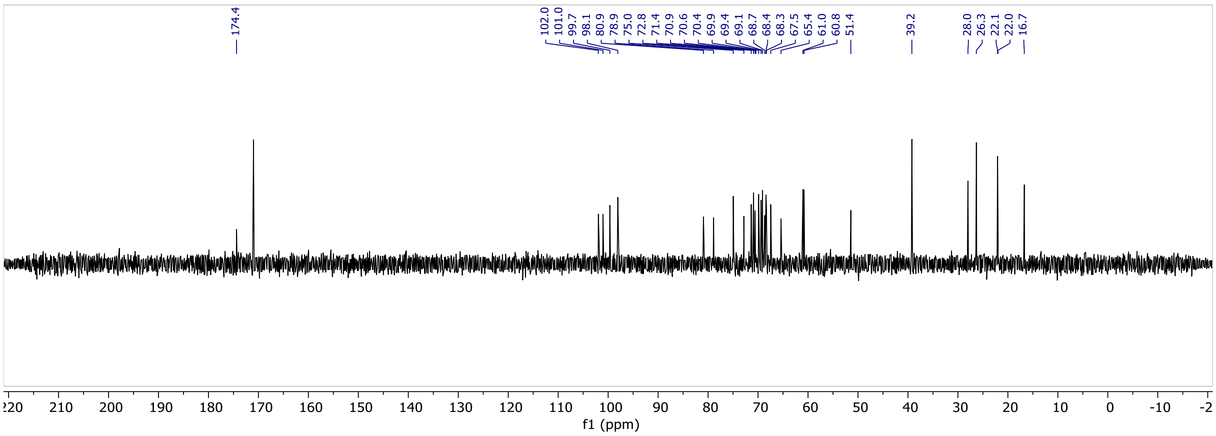

Coupled $^{13}C,^{1}H$ HSQC of **LPS-2**:

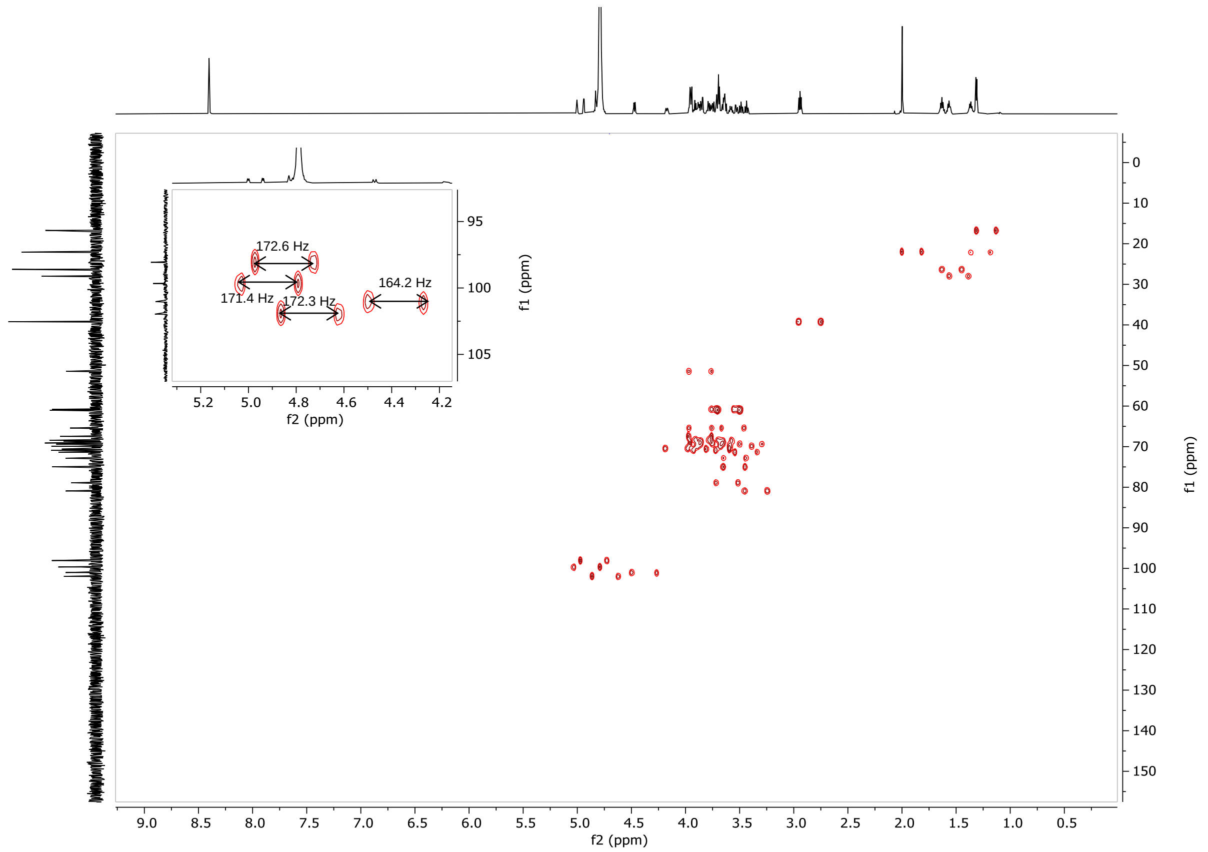

$^{13}C,^{1}H$ HSQC of **LPS-2**:

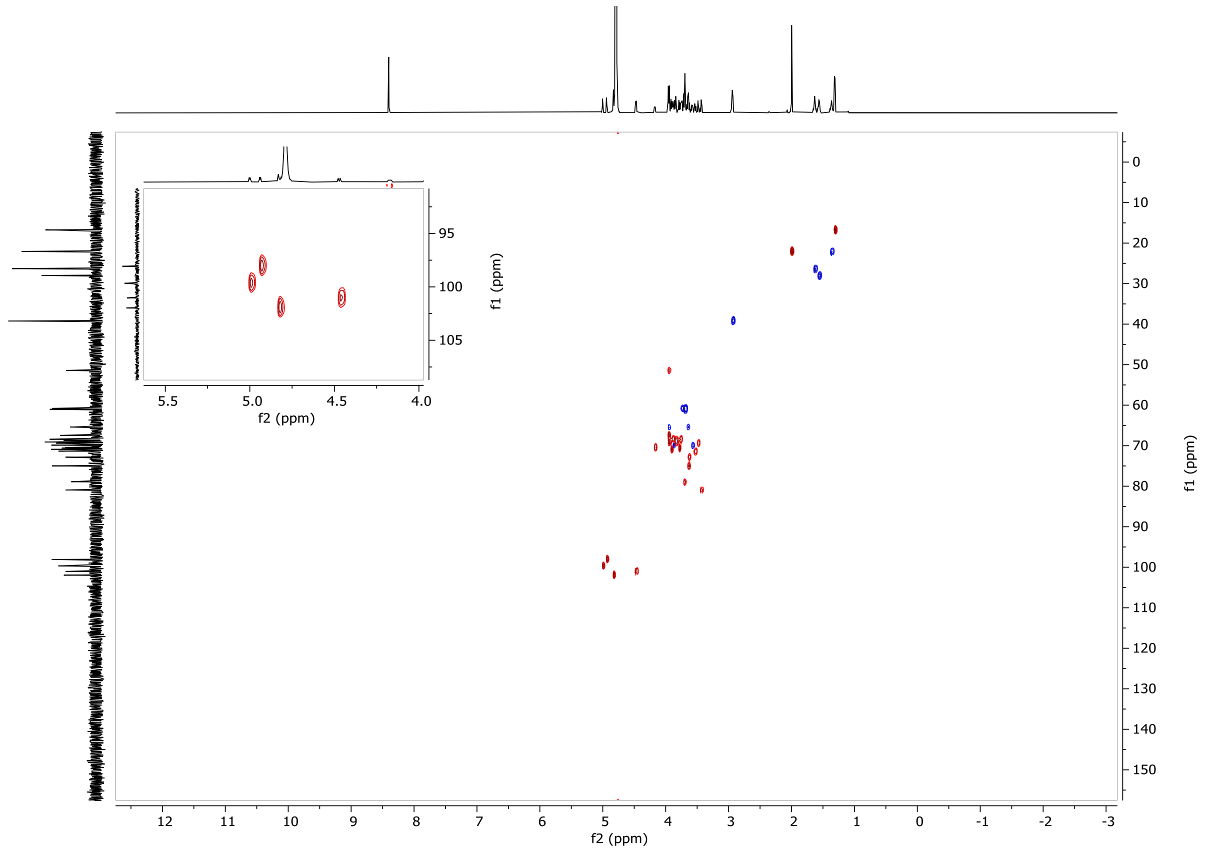

5-Amino-pentyl 2-acetamido-2-deoxy-β-D-galactopyranosyl-(1→3)-α-D-galactopyranosyl-(1→6)-α-D-glucopyranosyl-(1→4)-α-L-rhamnopyranoside (LPS-3)

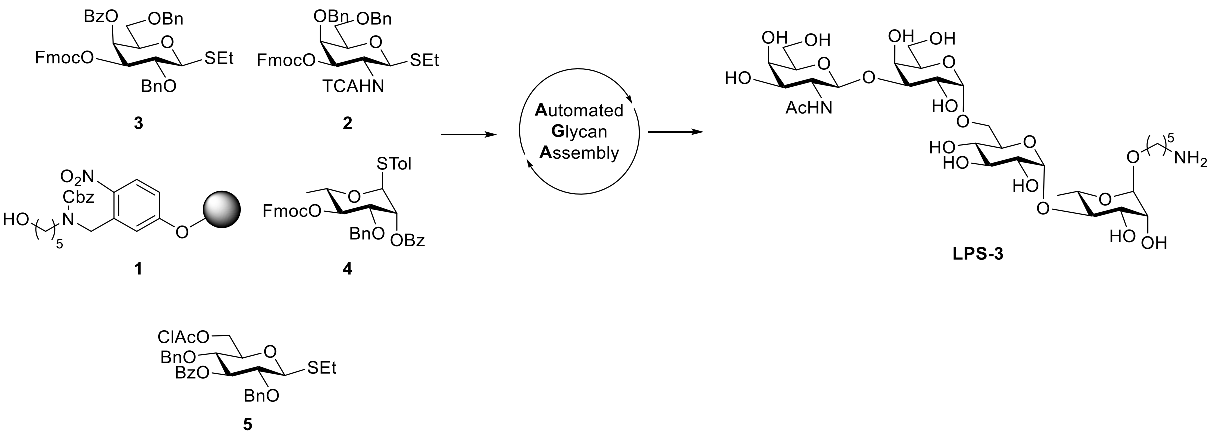

Repeat	Building Blocks	Modules	Notes
1x		**I** – Acidic Wash	
	**4** (2 x 5.0 equiv.)	**IIa** – Glycosylation with thioglycoside – 2 cycles	-20 °C (T_1) 10 min (t_1)<br>0 °C (T_2) 30 min (t_2)
		**III** – Capping<br>**IVc** – Fmoc Deprotection	
1x		**I** – Acidic Wash	
	**5** (2 x 5.0 equiv.)	**IIa** – Glycosylation with thioglycoside – 2 cycles	-20 °C (T_1) 5 min (t_1)<br>0 °C (T_2) 30 min (t_2)
		**III** – Capping<br>**IVe** – ClAc Deprotection	
1x		**I** – Acidic Wash	
	**3** (2 x 5.0 equiv.)	**IIa** – Glycosylation with thioglycoside – 2 cycles	-40 °C (T_1) 10 min (t_1)<br>-10 °C (T_2) 50 min (t_2)
		**III** – Capping<br>**IVc** – Fmoc Deprotection	
1x		**I** – Acidic Wash	
	**2** (3 x 5.0 equiv.)	**IIa** – Glycosylation with thioglycoside – 3 cycles	-40 °C (T_1) 30 min (t_1)<br>-20 °C (T_2) 20 min (t_2)

Protected **LPS-3** (27 mg, 0.012 mmol, crude yield: 92%) was obtained as a colorless oil after photocleavage from solid support following **Method A-1**. Deprotection of **LPS-3** following **Method C** and **D** and purification by reverse-phase HPLC (**Method E-1**, t_R = 19.4 min) afforded deprotected compound **LPS-3** (1.8 mg, 0.002 mmol, 18%) as a white solid after lyophylization.

^{1}H NMR (700 MHz, D_2O): δ 5.01 (d, J = 3.9 Hz, 1H), 4.91 (d, J = 3.9 Hz, 1H), 4.76 – 4.74 (m, 1H), 4.60 (d, J = 8.5 Hz, 1H), 4.19 – 4.14 (m, 2H), 3.98 (dd, J = 11.3, 3.5 Hz, 1H), 3.94 – 3.60 (m, 18H), 3.57 – 3.48 (m, 3H), 3.44 (t, J = 9.4 Hz, 1H), 2.96 (t, J = 7.7 Hz, 2H), 1.99 (s, 2H), 1.68 – 1.56 (m, 4H), 1.41 (d, J = 7.9 Hz, 2H), 1.33 (d, J = 6.3 Hz, 3H) ppm.

^{13}C NMR (176 MHz, D_2O): δ 175.1, 103.2, 99.7, 99.4, 98.1, 81.1, 79.1, 74.9, 72.9, 71.3, 70.7, 70.5, 70.4, 69.2, 69.1, 69.0, 67.6, 67.5, 67.4, 65.1, 60.9, 52.6, 39.2, 28.0, 26.5, 22.3, 22.2, 16.6 ppm.

HRMS (QToF): Calcd for $C_{31}H_{57}N_2O_{20}$ $[M + H]^+$ 777.3499; found 777.3501.

RP-HPLC of crude deprotected **LPS-3** (ELSD trace, **Method E-1**, t_R = 19.4 min):

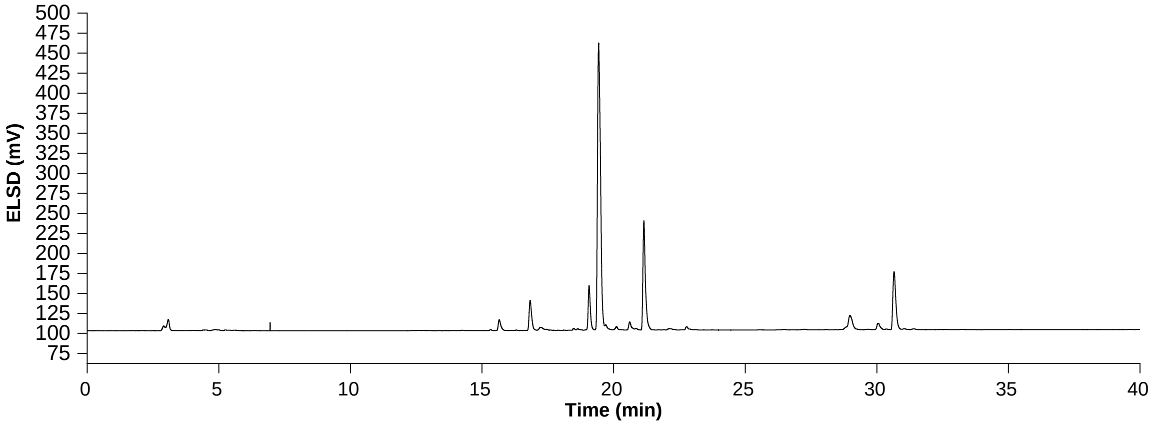

RP-HPLC of purified deprotected **LPS-3** (ELSD trace, **Method E-1**, t_R = 19.4 min):

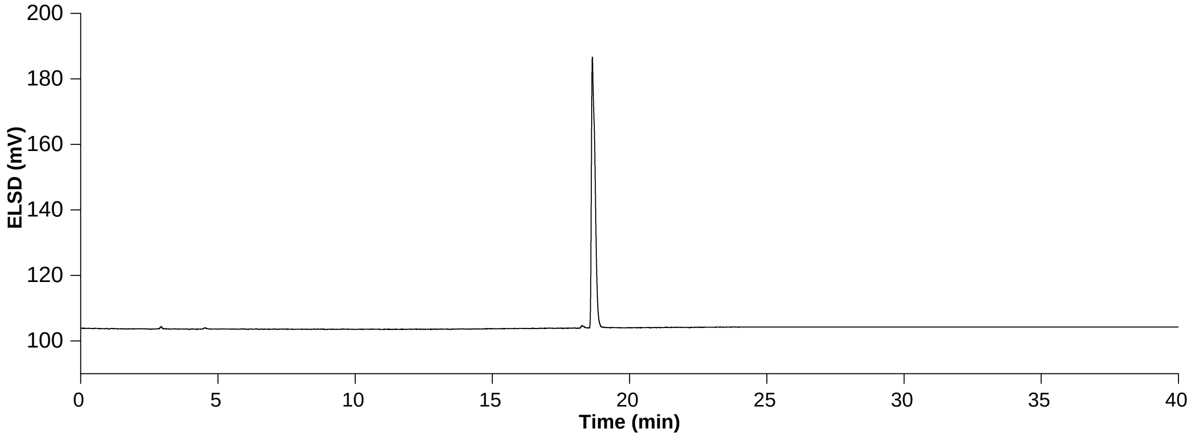

^{1}H NMR (700 MHz, D_2O) of **LPS-3**:

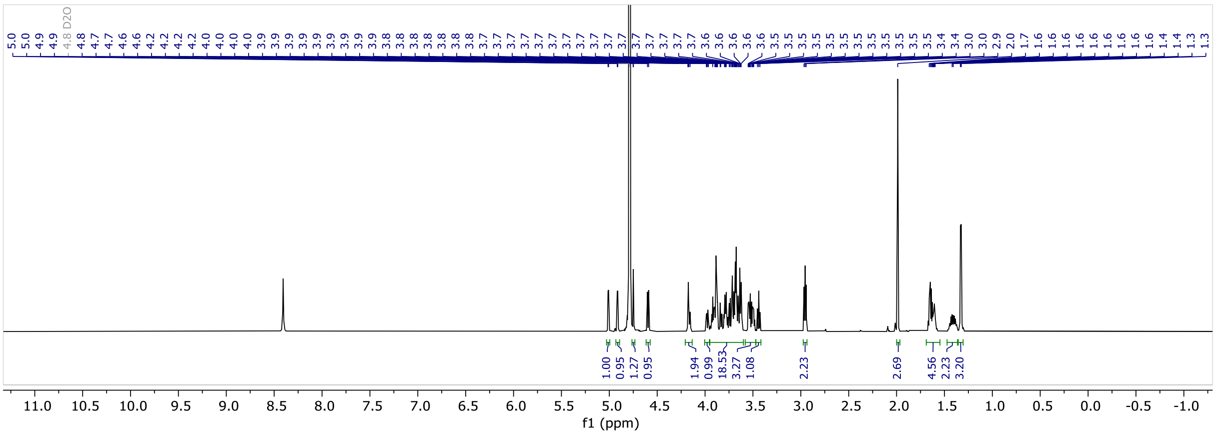

^{13}C NMR (176 MHz, D_2O) of **LPS-3**:

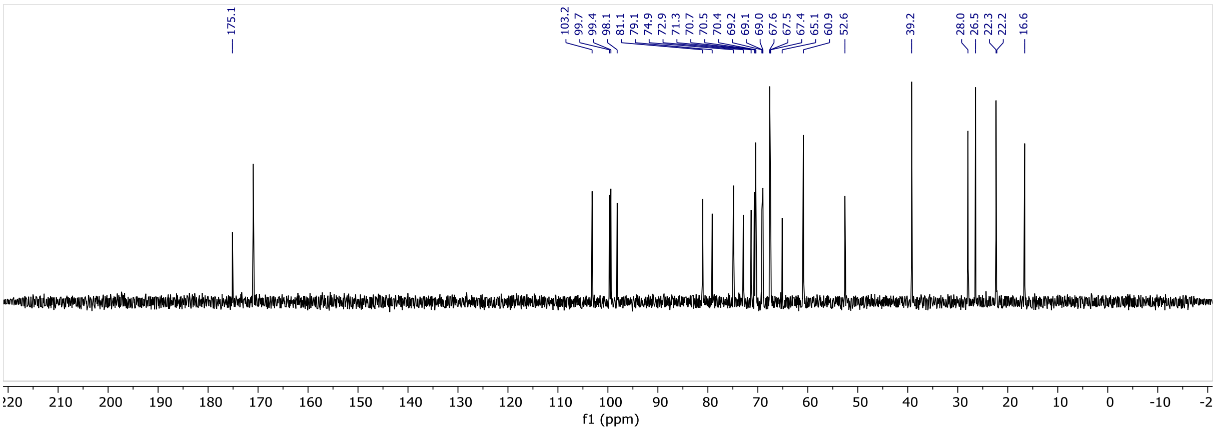

Coupled $^{13}C,^{1}H$ HSQC of **LPS-3**:

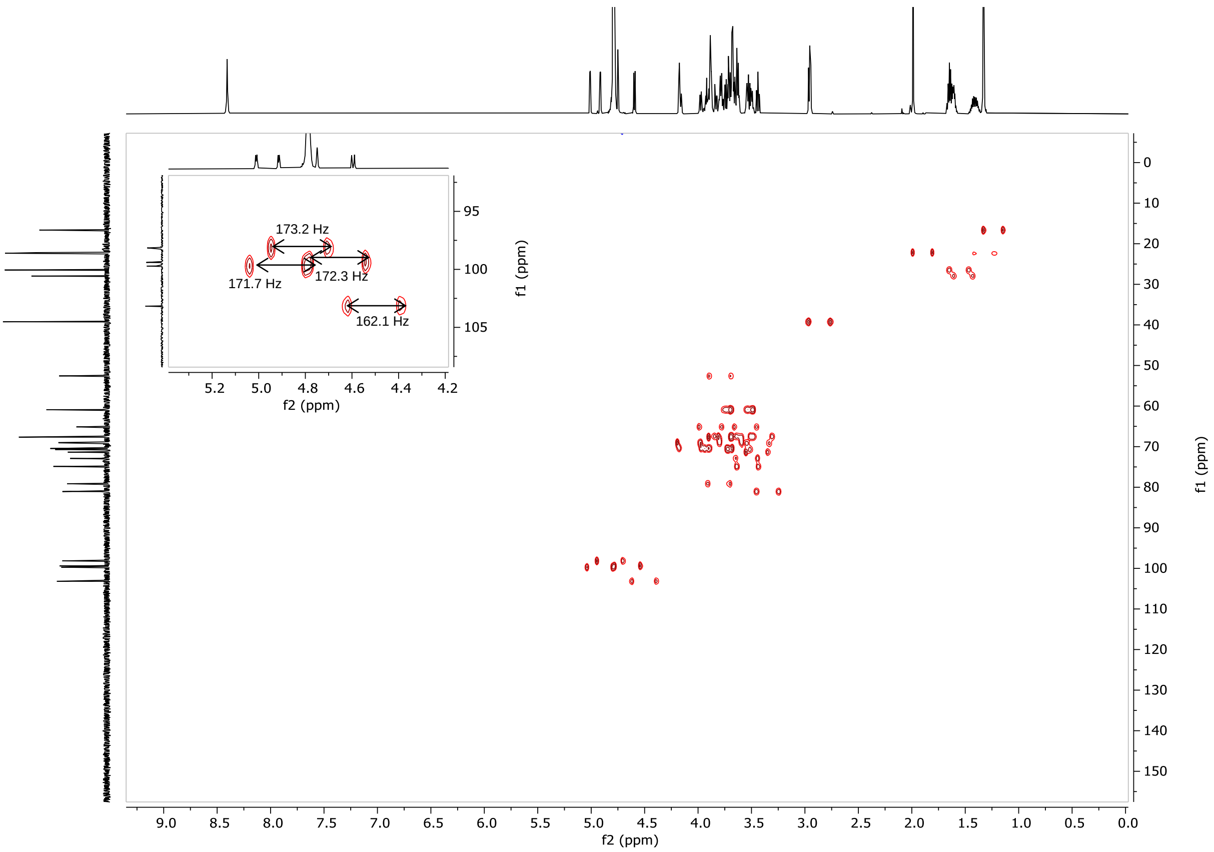

$^{13}C,^{1}H$ HSQC of **LPS-3**:

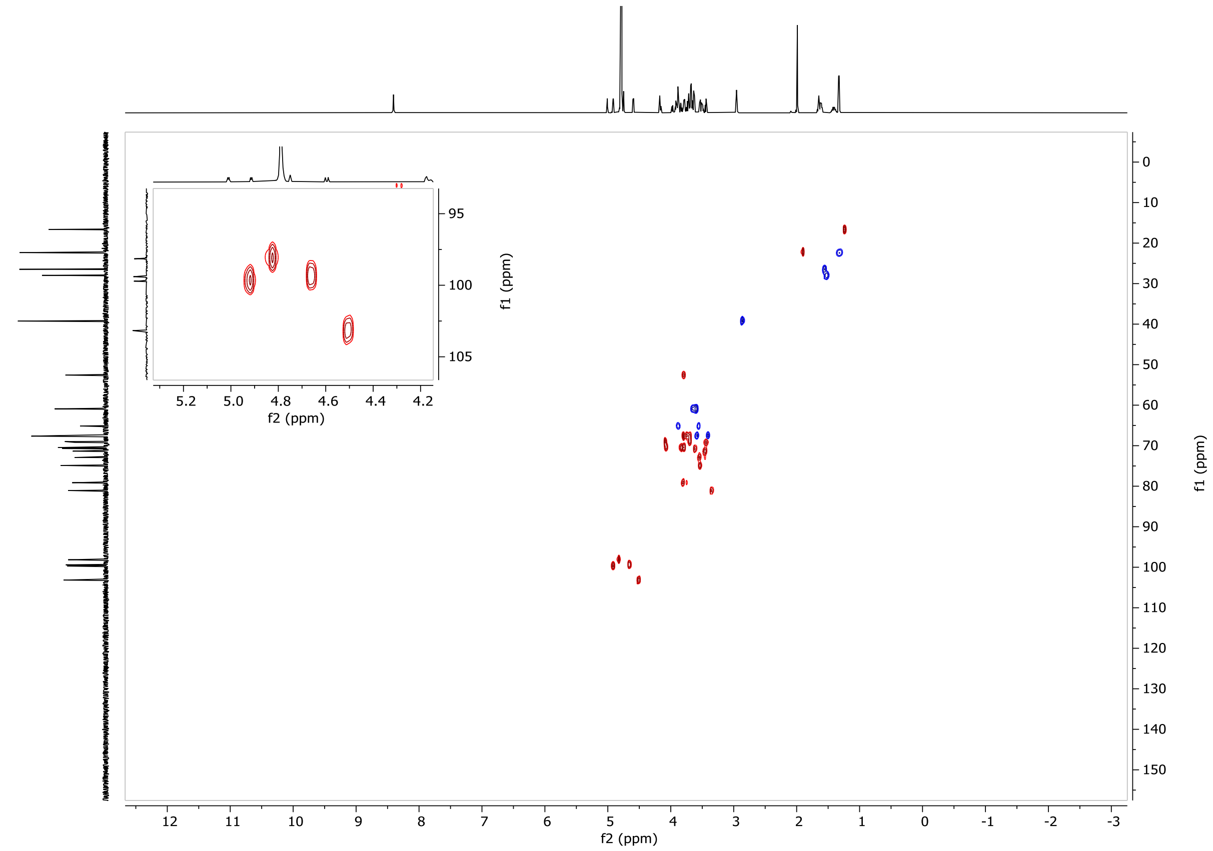

5-Amino-pentyl α-L-rhamnopyranosyl-(1→3)-2-acetamido-2-deoxy-β-D-galactopyranosyl-(1→3)-α-D-galactopyranosyl-(1→6)-α-D-glucopyranoside (LPS-4)

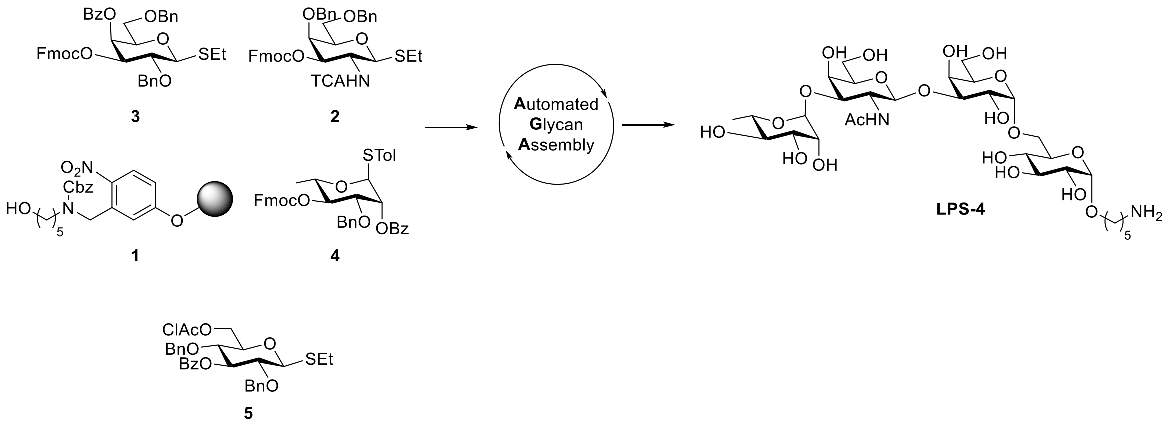

Repeat	Building Blocks	Modules	Notes
1x		**I** – Acidic Wash	
	**5** (2 x 5.0 equiv.)	**IIa** – Glycosylation with thioglycoside – 2 cycles	-20 °C (T_1) 5 min (t_1)<br>0 °C (T_2) 30 min (t_2)
		**III** – Capping<br>**IVe** – ClAc Deprotection	
1x		**I** – Acidic Wash	
	**3** (2 x 5.0 equiv.)	**IIa** – Glycosylation with thioglycoside – 2 cycles	-40 °C (T_1) 10 min (t_1)<br>-10 °C (T_2) 50 min (t_2)
		**III** – Capping<br>**IVc** – Fmoc Deprotection	
1x		**I** – Acidic Wash	
	**2** (2 x 5.0 equiv.)	**IIa** – Glycosylation with thioglycoside – 2 cycles	-40 °C (T_1) 30 min (t_1)<br>-20 °C (T_2) 20 min (t_2)
		**III** – Capping<br>**IVc** – Fmoc Deprotection	
1x		**I** – Acidic Wash	
	**4** (2 x 5.0 equiv.)	**IIa** – Glycosylation with thioglycoside – 2 cycles	-20 °C (T_1) 10 min (t_1)<br>0 °C (T_2) 30 min (t_2)

Protected **LPS-4** (17 mg, 0.012 mmol, crude yield: 57%) was obtained as a colorless oil after photocleavage from solid support following **Method A-1**. Deprotection of **LPS-3** following **Method C** and **D** and purification by reverse-phase HPLC (**Method E-1**, t_R = 19.4 min) afforded deprotected compound **LPS-3** (0.8 mg, 0.001 mmol, 8%) as a white solid after lyophylization.

^{1}H NMR (700 MHz, D_2O): δ 4.88 (d, *J* = 3.8 Hz, 1H), 4.86 (d, *J* = 4.1 Hz, 1H), 4.64 (d, *J* = 8.5 Hz, 1H), 4.15 (s, 1H), 4.02 – 3.83 (m, 6H), 3.82 – 3.59 (m, 14H), 3.52 – 3.44 (m, 3H), 3.36 (t, *J* = 9.7 Hz, 1H), 2.93 (t, *J* = 7.6 Hz, 2H), 1.97 (s, 3H), 1.63 (p, *J* = 7.4 Hz, 4H), 1.40 (dt, *J* = 16.0, 8.4 Hz, 2H), 1.20 (d, *J* = 6.3 Hz, 3H) ppm.

^{13}C NMR (176 MHz, D_2O): δ 174.8, 102.6, 102.3, 98.2, 98.1, 73.3, 70.0, 69.1, 67.9, 60.9, 51.8, 46.5, 39.4, 38.7, 28.0, 26.6, 22.5, 16.6 ppm.

HRMS (QToF): Calcd for $C_{31}H_{57}N_2O_{20}$ $[M + H]^+$ 777.3499; found 777.3502.

RP-HPLC of crude deprotected **LPS-4** (ELSD trace, **Method E-1**, t_R = 16.0 min):

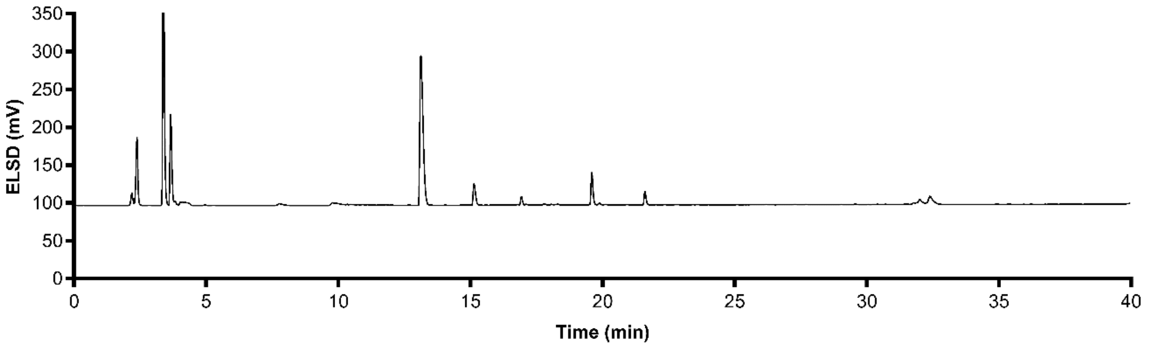

RP-HPLC of purified deprotected **LPS-4** (ELSD trace, **Method E-1**, t_R = 16.0 min):

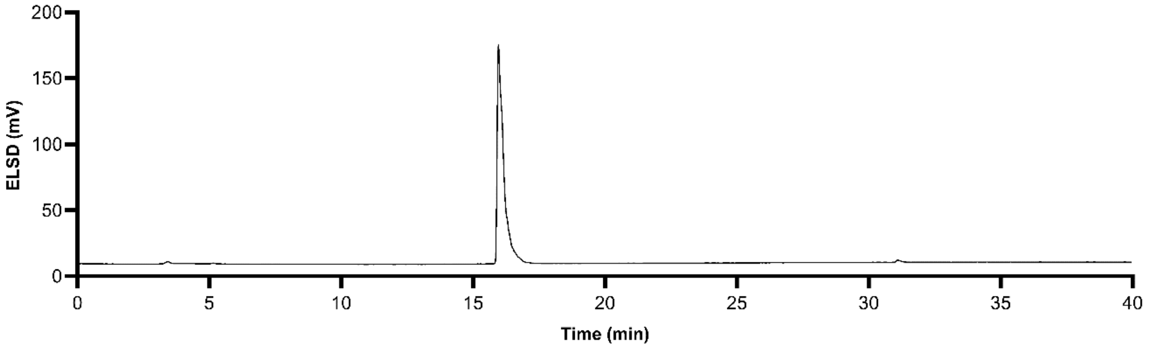

^{1}H NMR (700 MHz, D_2O) of **LPS-4**:

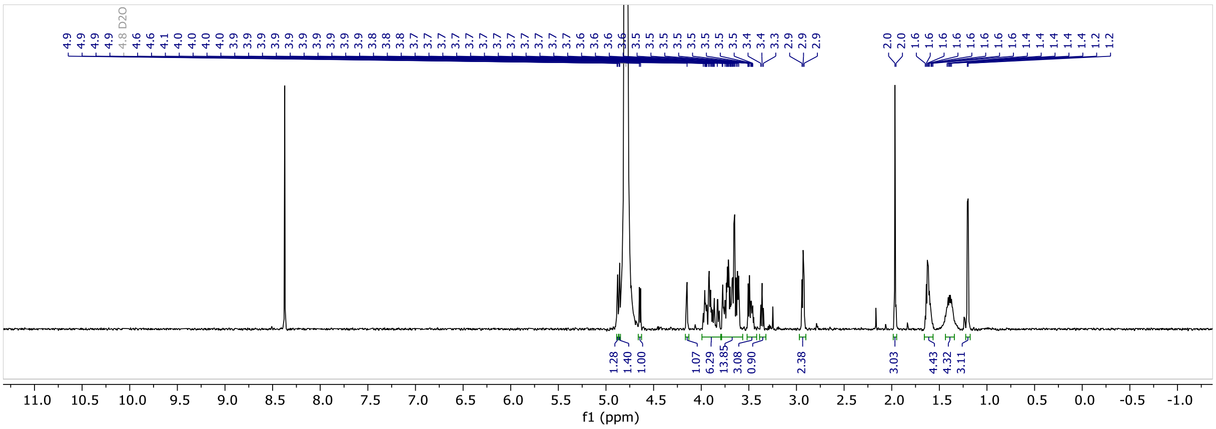

^{13}C NMR (176 MHz, D_2O) of **LPS-4**:

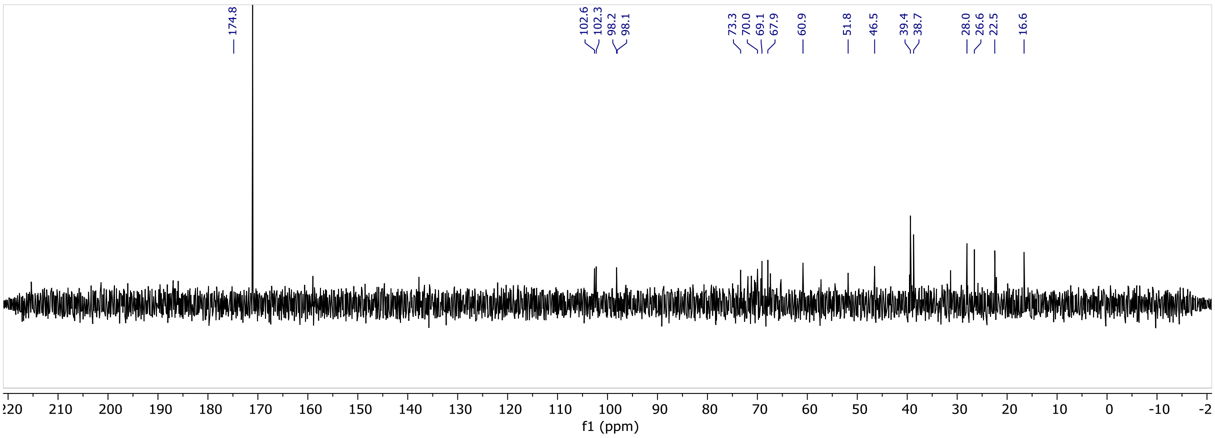

Coupled $^{13}C,^{1}H$ HSQC of **LPS-4**:

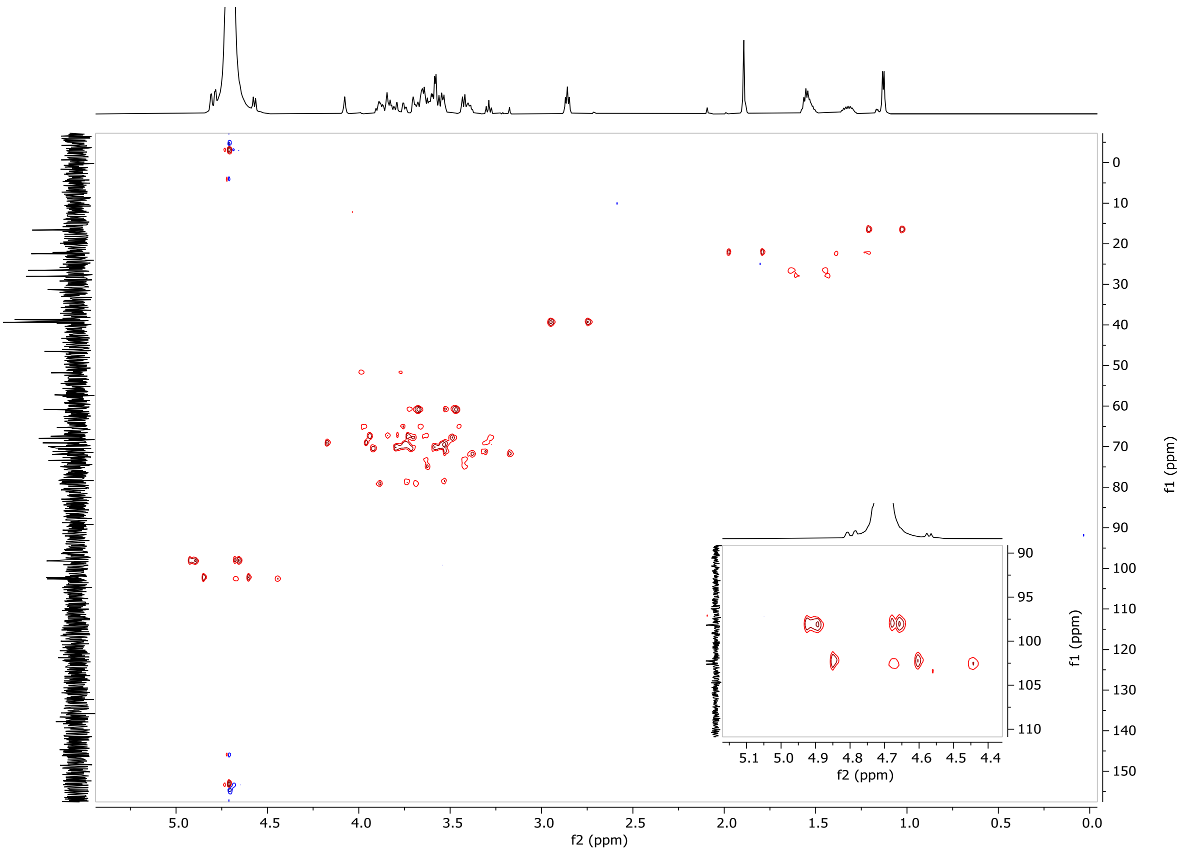

$^{13}C,^{1}H$ HSQC of **LPS-4**:

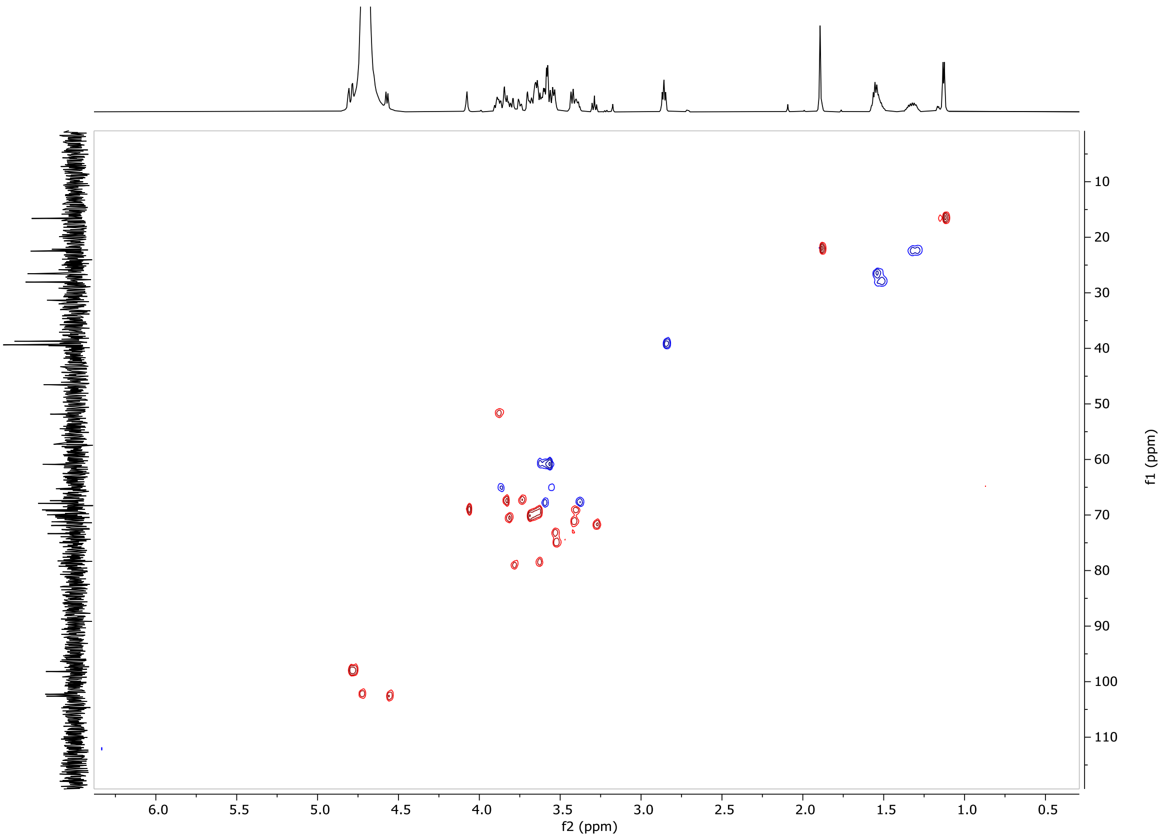

5-Amino-pentyl α-D-galactopyranoside (LPS-11)

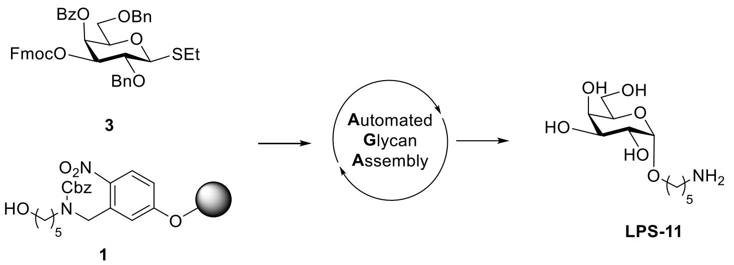

Repeat	Building Blocks	Modules	Notes	
1x		**I** – Acidic Wash		
	**3** (2 x 5.0 equiv.)	**IIa** – Glycosylation with thioglycoside – 2 cycles	-40 °C (T_1)	10 min (t_1)
			-10 °C (T_2)	50 min (t_2)

Protected **LPS-11** (8 mg, 0.012 mmol, crude yield: 87%) was obtained as a colorless oil after photocleavage from solid support following **Method A-1**. Deprotection of **LPS-11** following **Method C** and **D** and purification by reverse-phase HPLC (**Method E-1**, t_R = 13.5 min) afforded deprotected compound **LPS-11** (1.2 mg, 0.004 mmol, 18%) as a white solid after lyophylization.

^{1}H NMR (700 MHz, D_2O): δ 4.95 (d, *J* = 3.4 Hz, 1H), 3.98 (d, *J* = 3.2 Hz, 1H), 3.93 (t, *J* = 6.2 Hz, 1H), 3.87 – 3.81 (m, 2H), 3.78 – 3.72 (m, 3H), 3.54 (dt, *J* = 10.0, 6.2 Hz, 1H), 3.01 (t, *J* = 7.6 Hz, 2H), 1.75 – 1.62 (m, 4H), 1.53 – 1.42 (m, 2H) ppm.

^{13}C NMR (176 MHz, D_2O): δ 98.2, 70.9, 69.5, 69.3, 68.3, 67.8, 61.3, 39.4, 28.0, 26.5, 22.4 ppm.

HRMS (QToF): Calcd for $C_{11}H_{24}NO_6$ $[M + H]^+$ 266.1598; found 266.1726.

RP-HPLC of crude deprotected **LPS-11** (ELSD trace, **Method E-1**, t_R = 13.5 min):

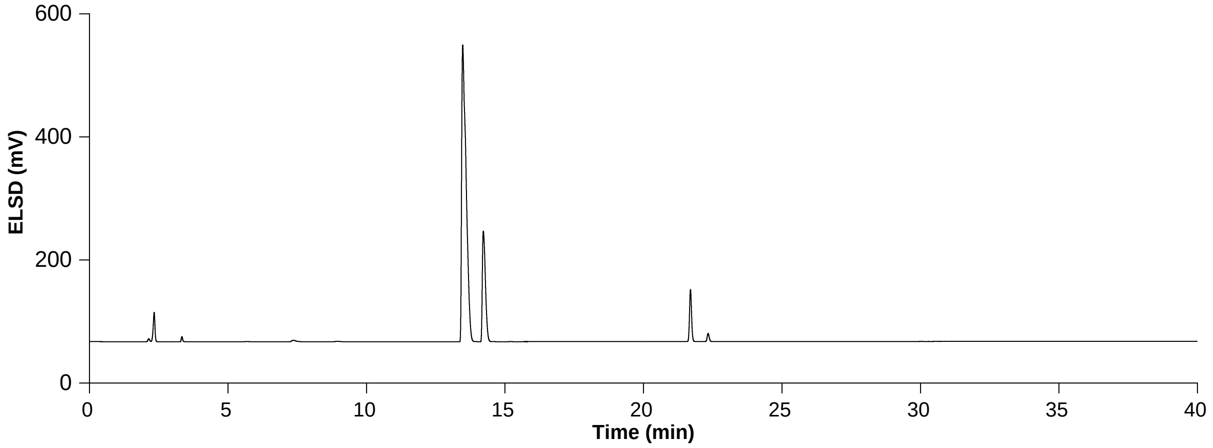

RP-HPLC of purified deprotected **LPS-11** (ELSD trace, **Method E-1**, t_R = 13.5 min):

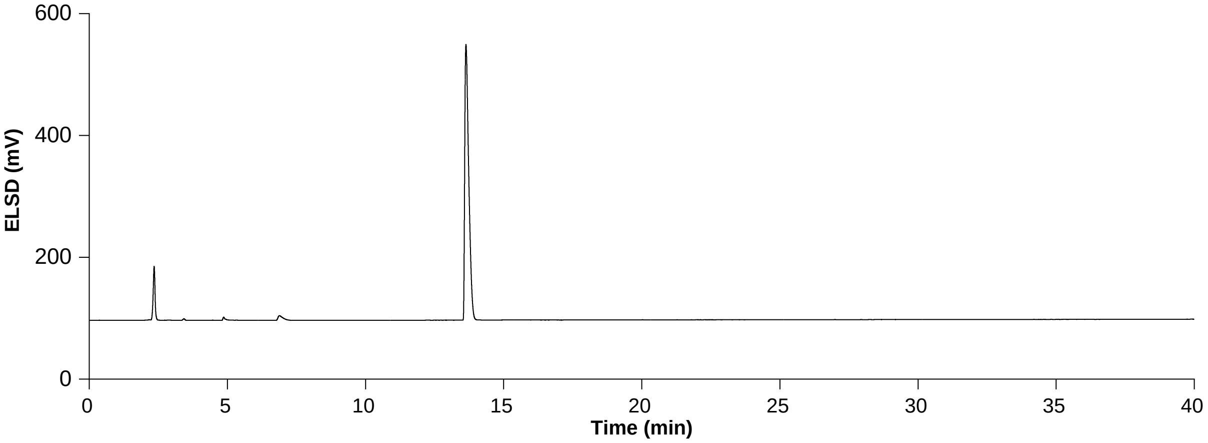

^{1}H NMR (700 MHz, D_2O) of **LPS-11**:

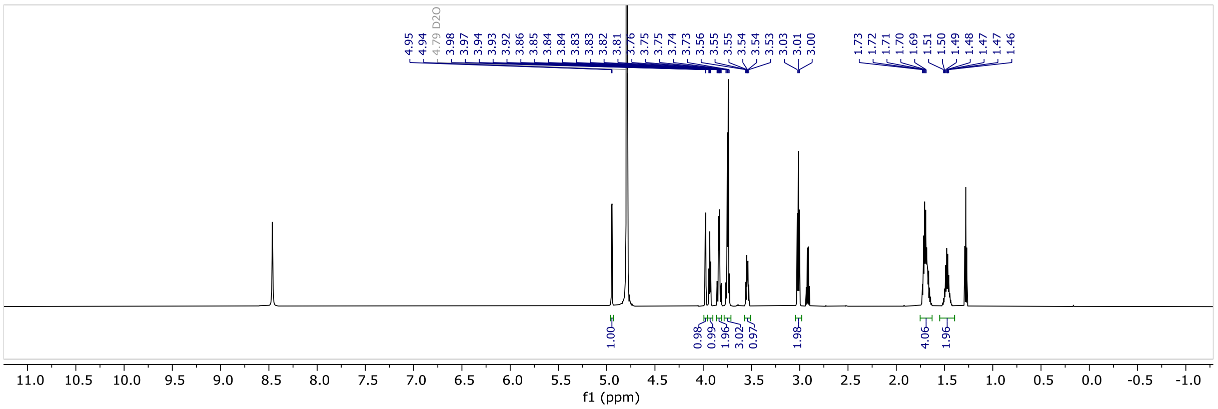

^{13}C NMR (176 MHz, D_2O) of **LPS-11**:

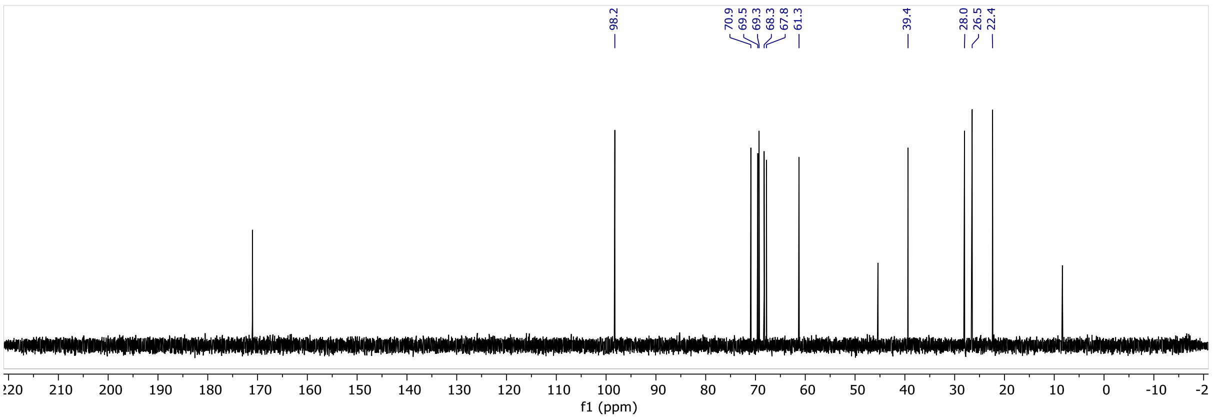

Coupled $^{13}C,^{1}H$ HSQC of **LPS-11**:

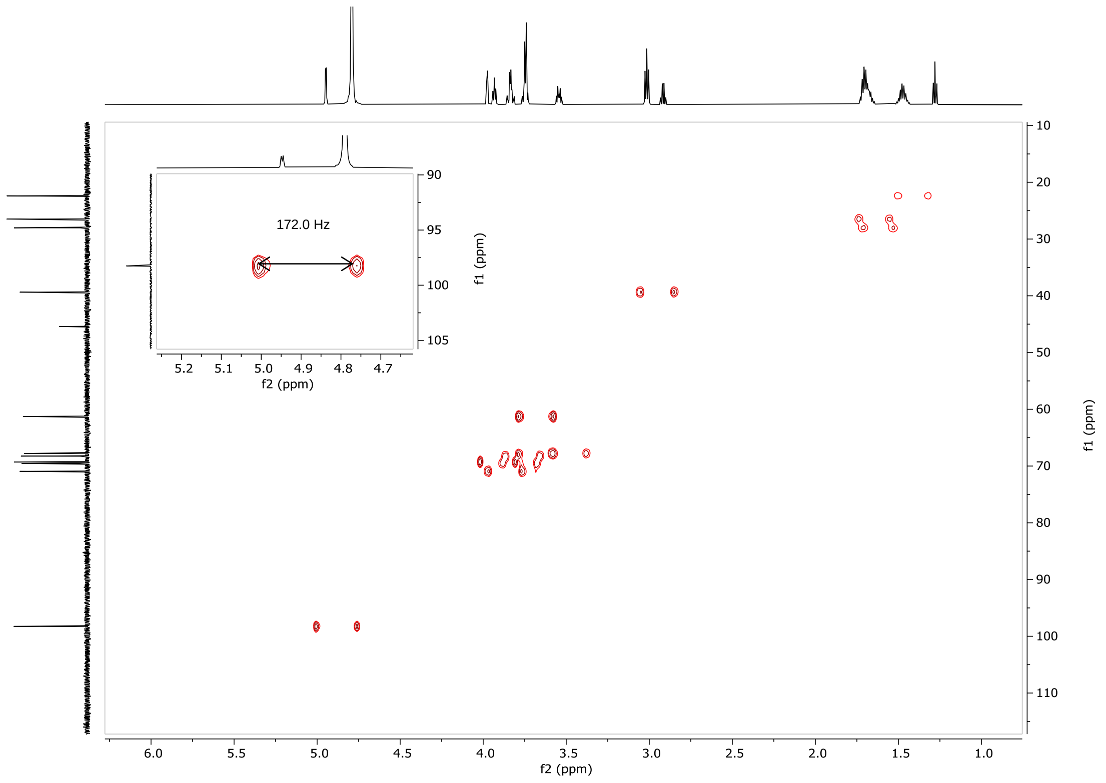

Coupled $^{13}C,^{1}H$ HSQC of **LPS-11**:

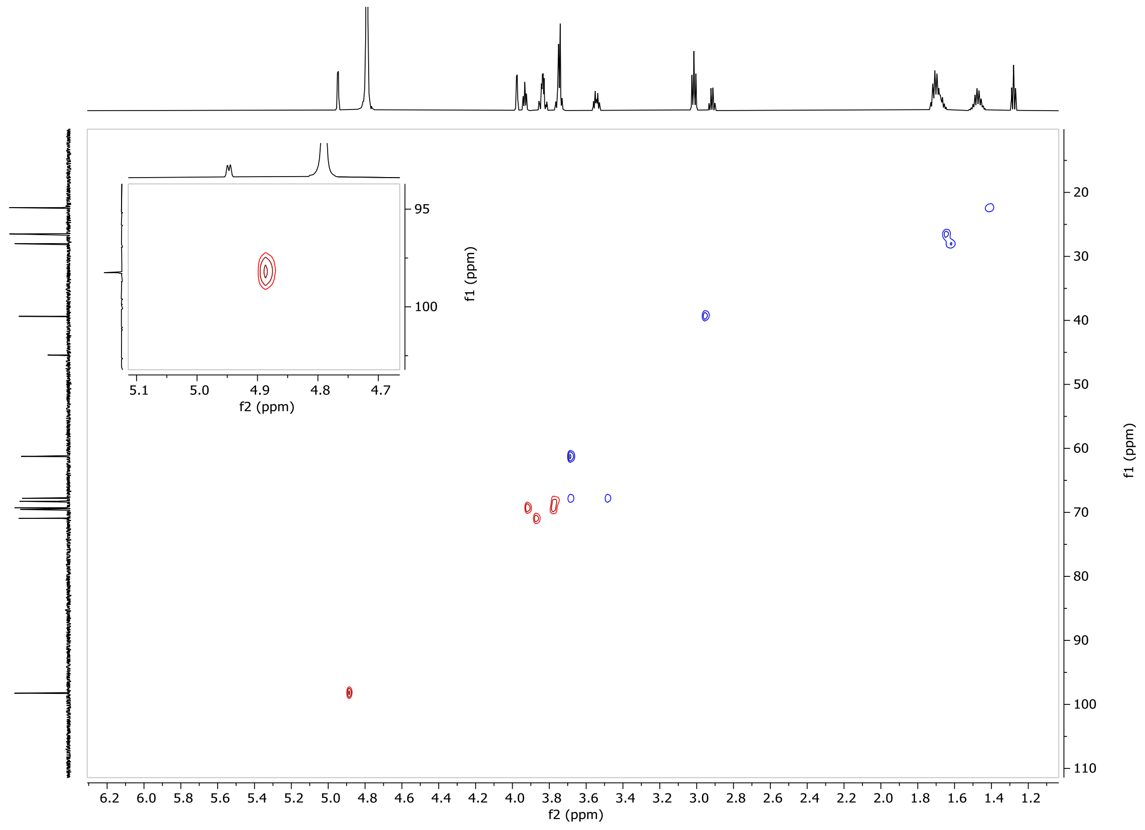

5-Amino-pentyl α-D-glucopyranoside (LPS-8)

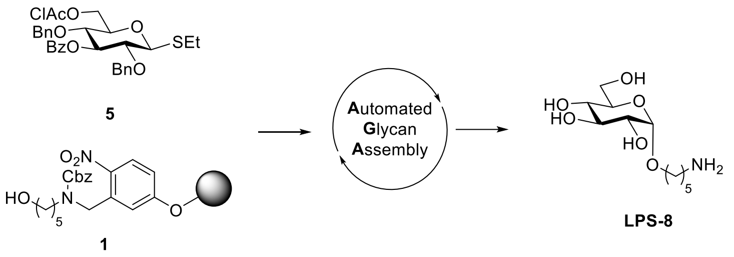

Repeat	Building Blocks	Modules	Notes
**1x**		**I** – Acidic Wash	
	**5** (2 x 5.0 equiv.)	**IIa** – Glycosylation with thioglycoside – 2 cycles	-20 °C (T_1) 5 min (t_1)<br>0 °C (T_2) 30 min (t_2)

Protected **LPS-8** (5 mg, 0.006 mmol, crude yield: 46%) was obtained as a colorless oil after photocleavage from solid support following **Method A-1**. Deprotection of **LPS-8** following **Method C** and **D** and purification by reverse-phase HPLC (**Method E-1**, t_R = 12.8 min) afforded deprotected compound **LPS-8** (0.9 mg, 0.003 mmol, 25%) as a white solid after lyophylization.

^{1}H NMR (700 MHz, D_2O): δ 4.92 (d, *J* = 3.8 Hz, 1H), 3.86 (dd, *J* = 12.3, 2.4 Hz, 1H), 3.78 – 3.65 (m, 4H), 3.59 – 3.52 (m, 2H), 3.41 (dd, *J* = 10.1, 9.1 Hz, 1H), 3.02 (t, *J* = 7.6 Hz, 2H), 1.72 – 1.63 (m, 4H), 1.54 – 1.41 (m, 2H) ppm.

^{13}C NMR (176 MHz, D_2O): δ 97.9, 73.0, 71.7, 71.1, 69.5, 67.6, 60.5, 39.2, 28.0, 26.4, 22.4 ppm.

HRMS (QToF): Calcd for $C_{11}H_{24}NO_6$ $[M + H]^+$ 266.1598; found 266.1750.

RP-HPLC of crude deprotected **LPS-8** (ELSD trace, **Method E-1**, t_R = 12.8 min):

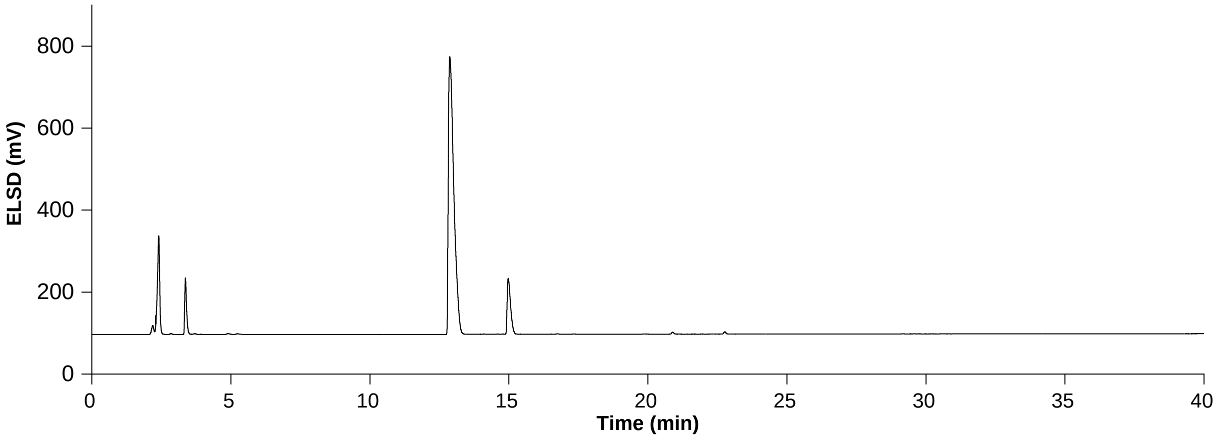

RP-HPLC of purified deprotected **LPS-8** (ELSD trace, **Method E-1**, t_R = 12.8 min):

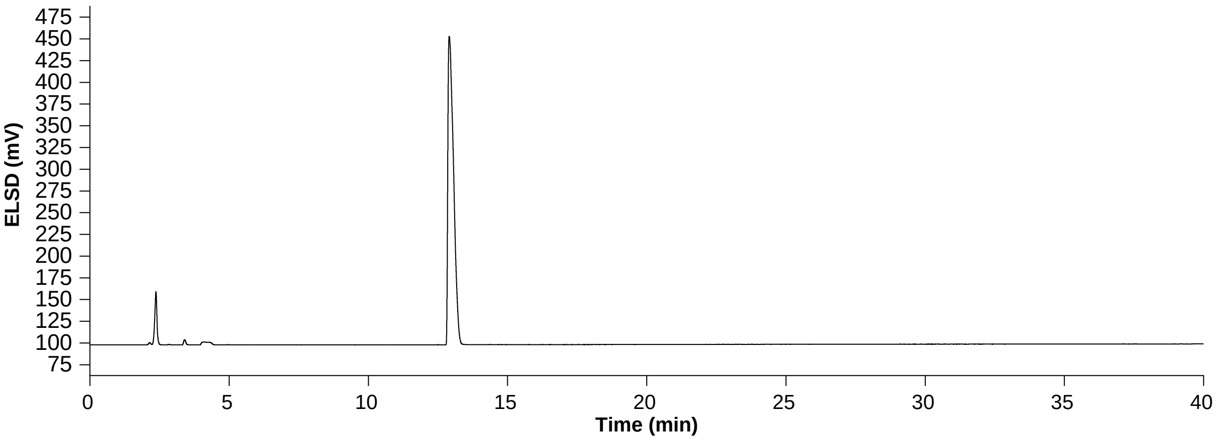

^1H NMR (700 MHz, D_2O) of **LPS-8**:

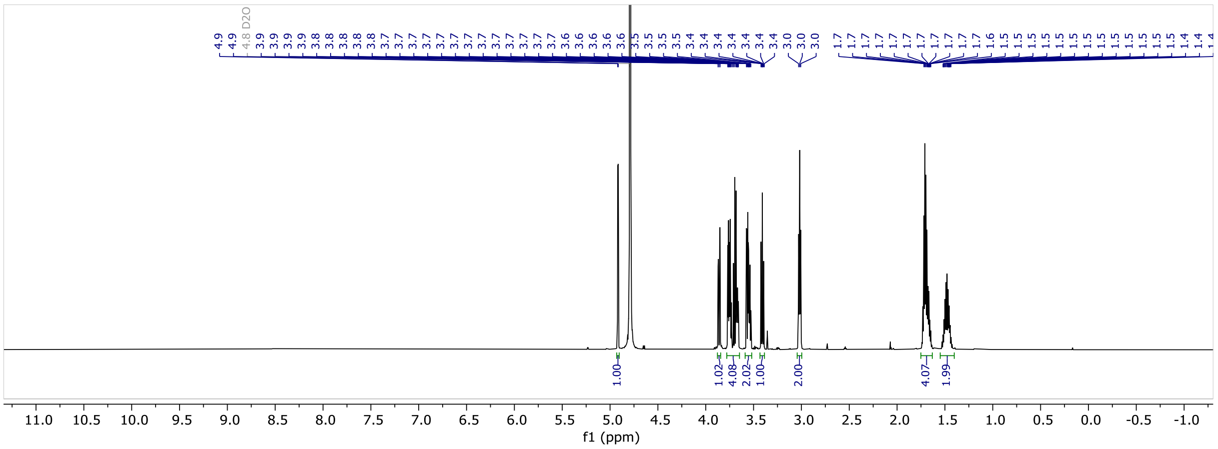

^{13}C NMR (176 MHz, D_2O) of **LPS-8**:

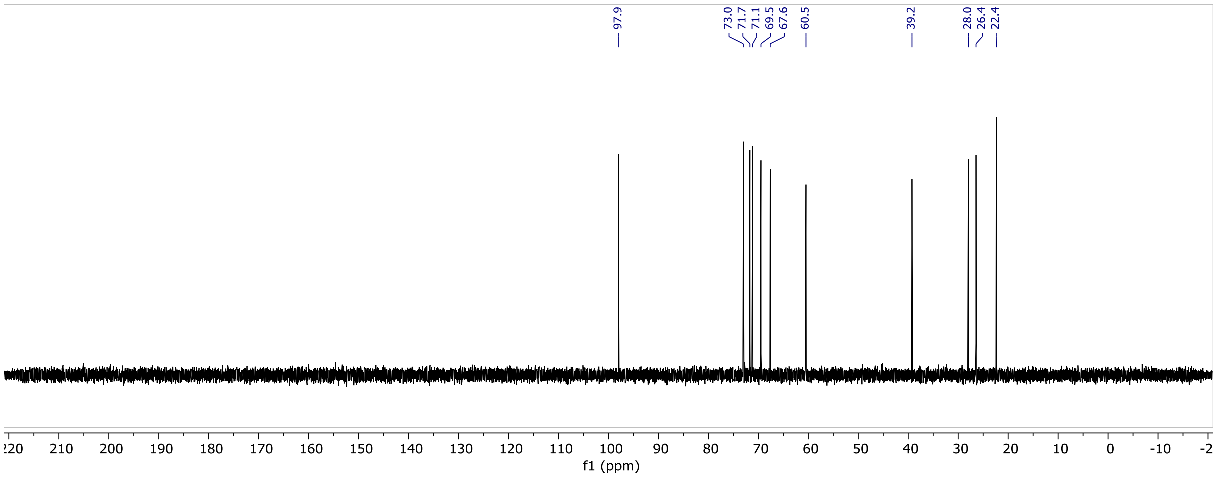

Coupled ^{13}C,^{1}H HSQC of **LPS-8**:

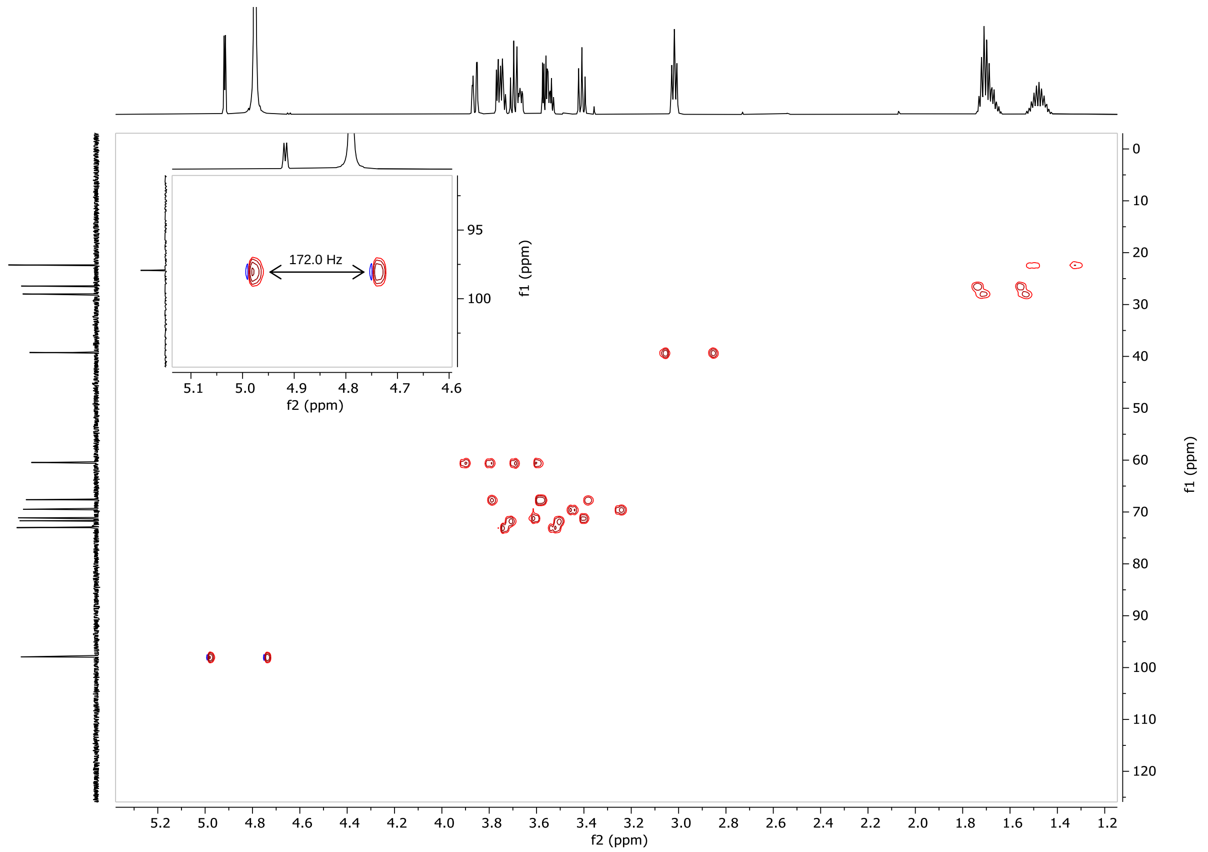

Coupled ^{13}C,^{1}H HSQC of **LPS-8**:

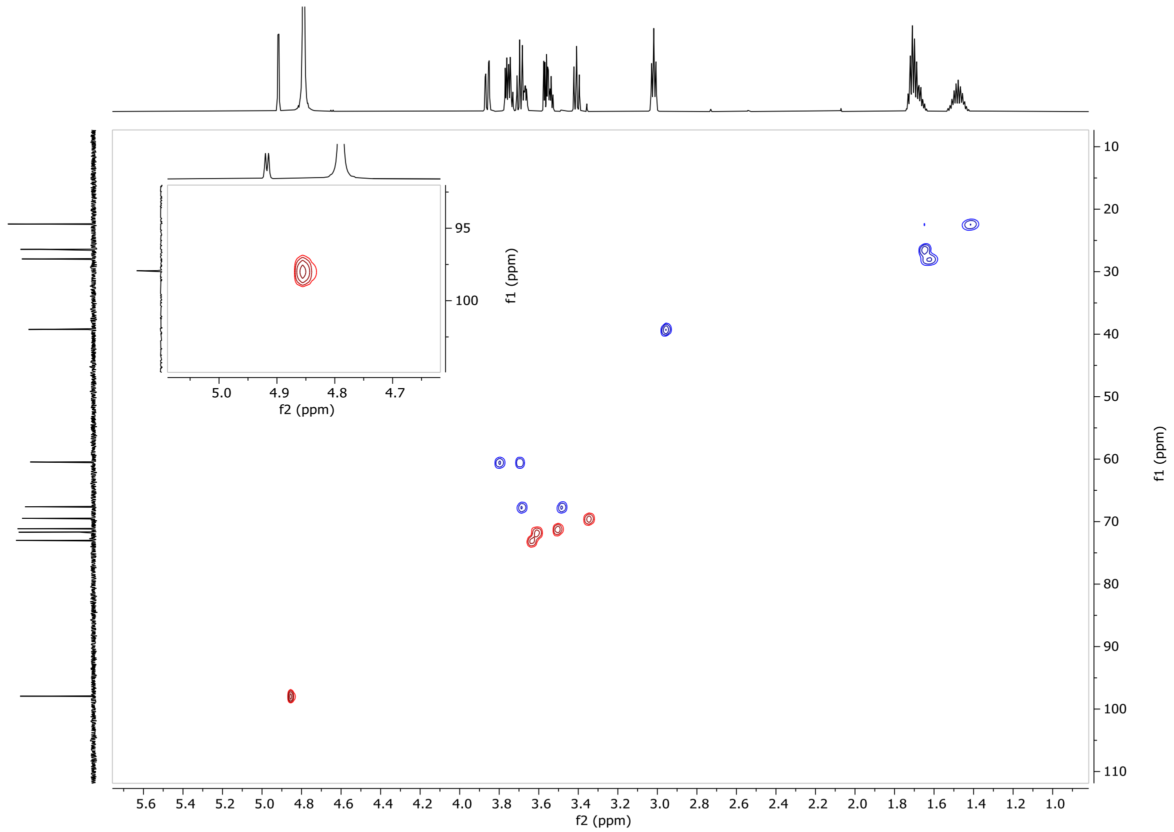

5-Amino-pentyl 2-acetamido-2-deoxy-β-D-galactopyranosyl-(1→3)-α-D-galactopyranoside (LPS-6)

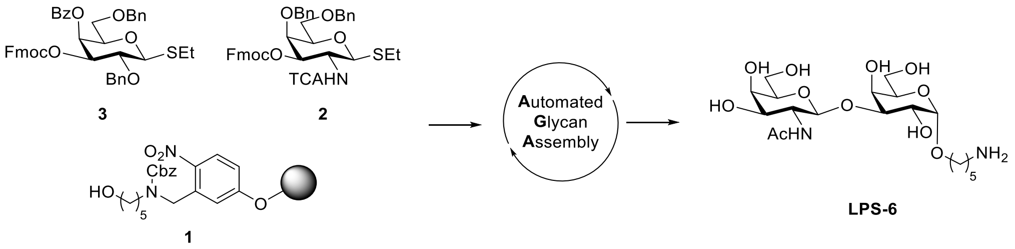

Repeat	Building Blocks	Modules	Notes	
1x		**I** – Acidic Wash		
	**3** (2 x 5.0 equiv.)	**IIa** – Glycosylation with thioglycoside – 2 cycles	-40 °C (T_1)<br>-10 °C (T_2)	10 min (t_1)<br>50 min (t_2)
		**III** – Capping<br>**IVc** – Fmoc Deprotection		
1x		**I** – Acidic Wash		
	**2** (2 x 5.0 equiv.)	**IIa** – Glycosylation with thioglycoside – 2 cycles	-40 °C (T_1)<br>-20 °C (T_2)	30 min (t_1)<br>20 min (t_2)
		**III** – Capping<br>**IVc** – Fmoc Deprotection		

Protected **LPS-6** (11 mg, 0.009 mmol, crude yield: 67%) was obtained as a colorless oil after photocleavage from solid support following **Method A-1**. Deprotection of **LPS-6** following **Method C** and **D** and purification by reverse-phase HPLC (**Method E-1**, t_R = 15.6 min) afforded deprotected compound **LPS-6** (1.1 mg, 0.002 mmol, 17%) as a white solid after lyophylization.

^{1}H NMR (700 MHz, D_2O): δ 4.92 (d, *J* = 3.8 Hz, 1H), 4.65 (d, *J* = 8.4 Hz, 1H), 4.20 (d, *J* = 3.2 Hz, 1H), 3.97 – 3.86 (m, 5H), 3.83 – 3.71 (m, 6H), 3.70 – 3.65 (m, 1H), 3.54 (dt, *J* = 9.9, 6.2 Hz, 1H), 3.01 (t, *J* = 7.6 Hz, 2H), 2.04 (s, 3H), 1.74 – 1.63 (m, 4H), 1.53 – 1.42 (m, 2H) ppm.

^{13}C NMR (176 MHz, D_2O): δ 175.2, 103.1, 98.4, 79.0, 75.0, 70.8, 70.5, 69.3, 67.8, 67.3, 61.2, 61.0, 52.7, 39.4, 28.1, 26.5, 22.4, 22.3 ppm.

HRMS (QToF): Calcd for $C_{19}H_{37}N_2O_{11}$ $[M + H]^+$ 469.2392; found 469.2402.

RP-HPLC of crude deprotected **LPS-6** (ELSD trace, **Method E-1**, t_R = 15.6 min):

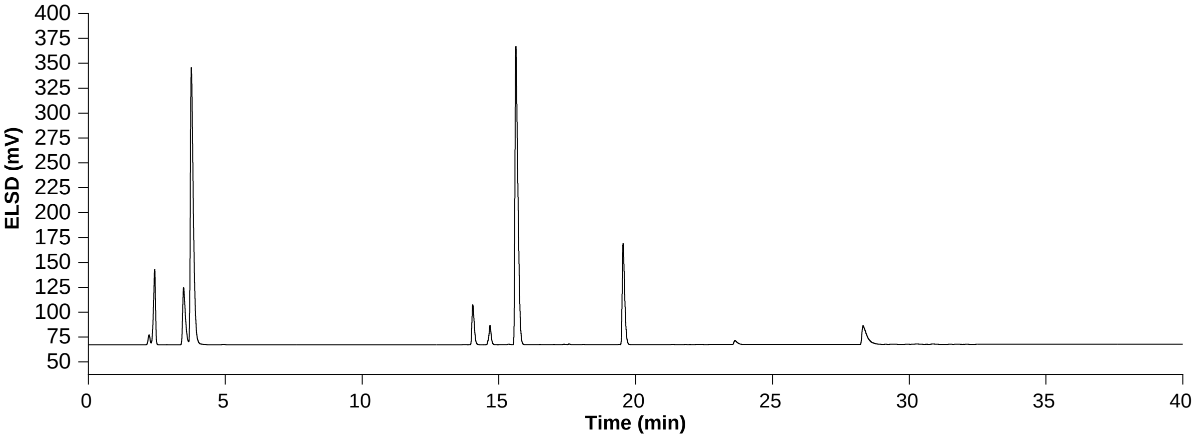

RP-HPLC of purified deprotected **LPS-6** (ELSD trace, **Method E-1**, t_R = 15.6 min):

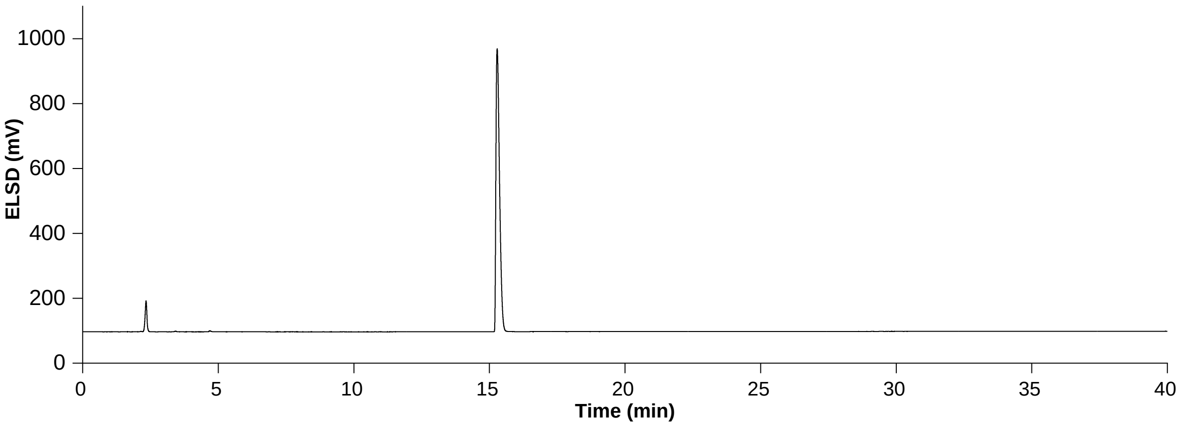

^{1}H NMR (700 MHz, D_2O) of **LPS-6**:

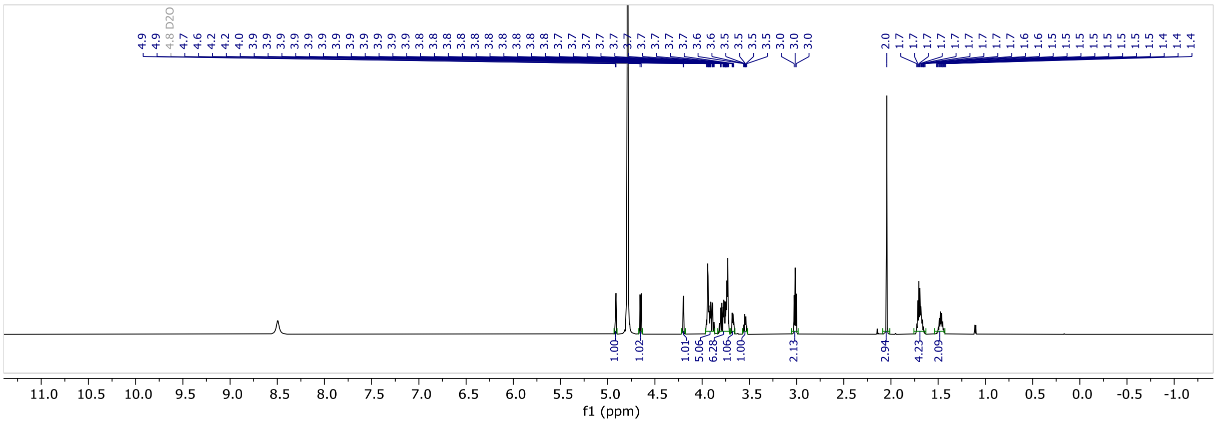

^{13}C NMR (176 MHz, D_2O) of **LPS-6**:

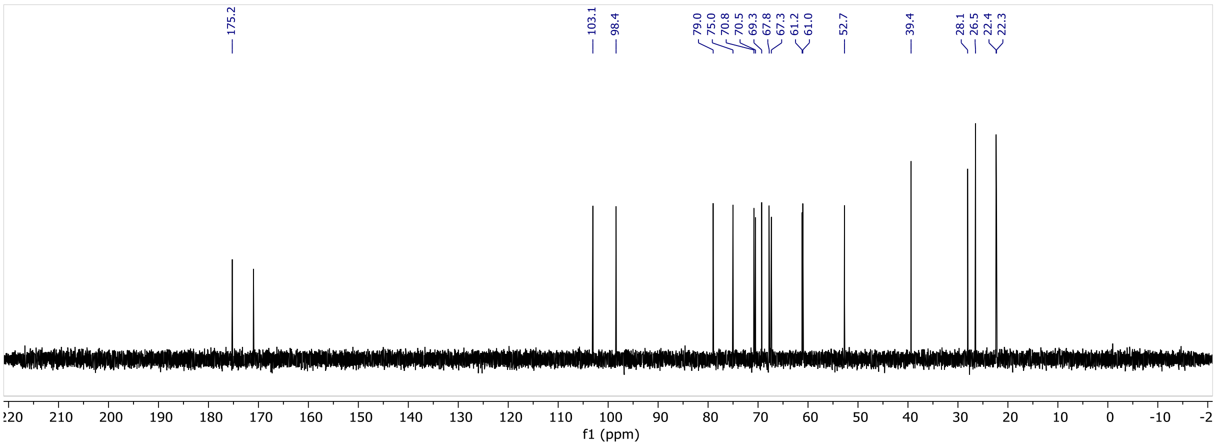

Coupled $^{13}C,^{1}H$ HSQC of **LPS-6**:

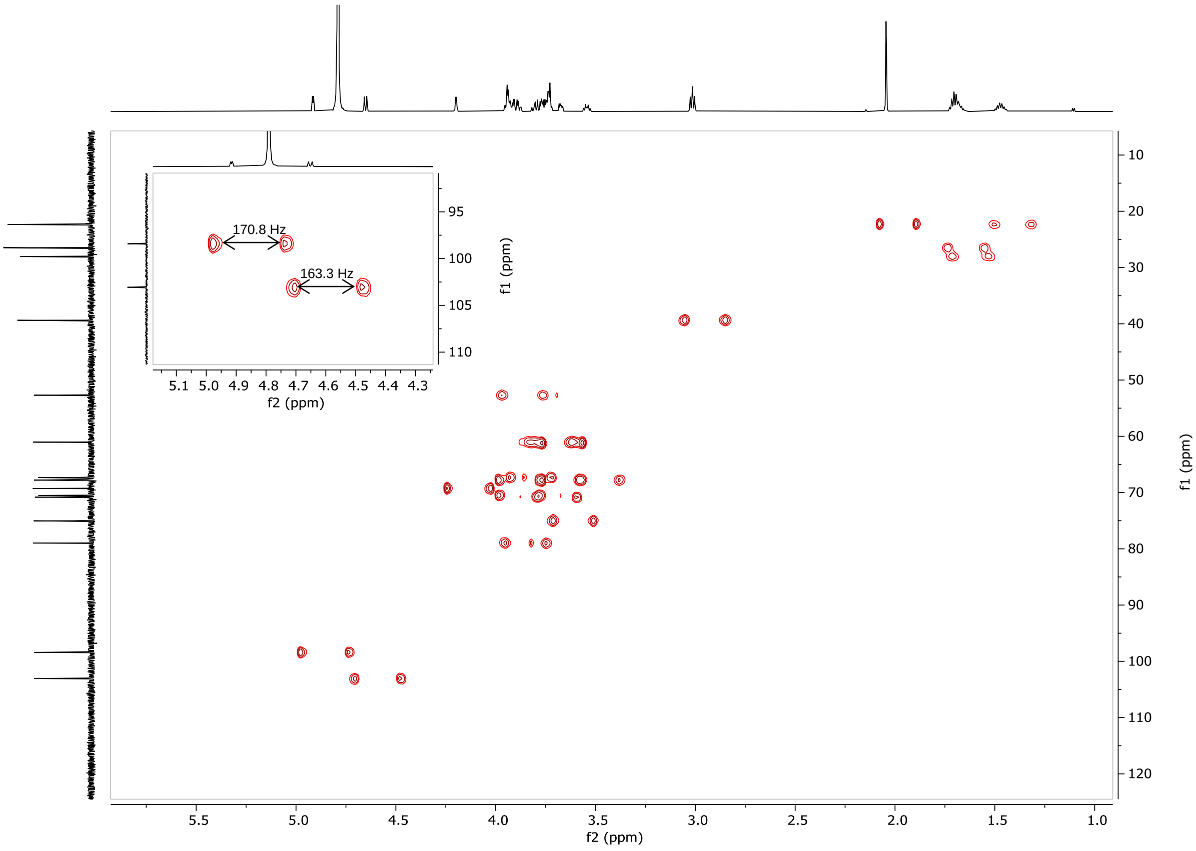

Coupled $^{13}C,^{1}H$ HSQC of **LPS-6**:

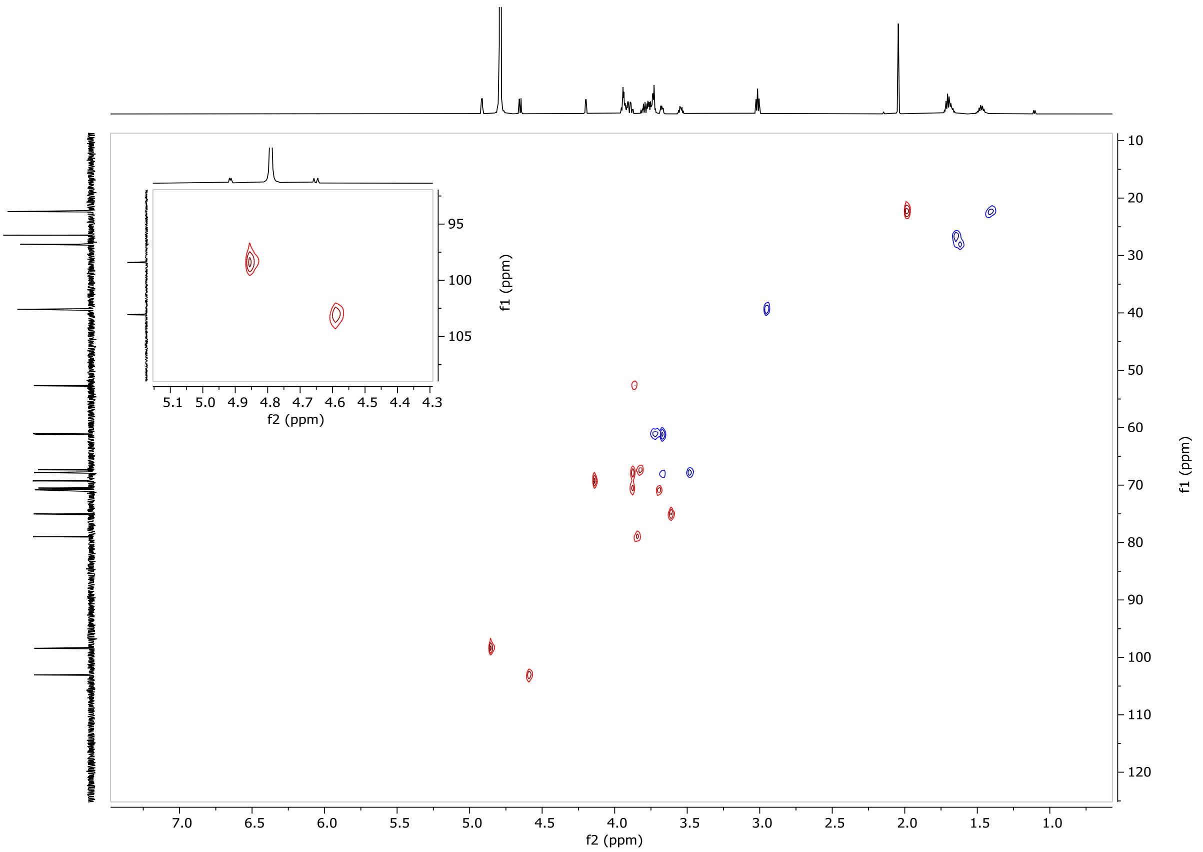

5-Amino-pentyl α-L-rhamnopyranosyl-(1→3)-2-acetamido-2-deoxy-β-D-galactopyranosyl-(1→3)-α-D-galactopyranoside (LPS-5)

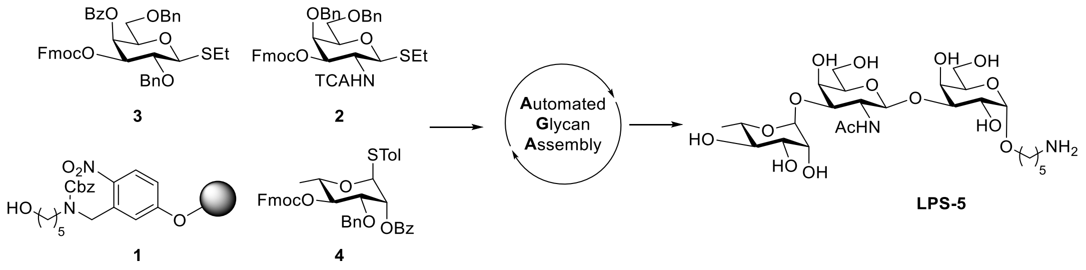

Repeat	Building Blocks	Modules	Notes	
1x		**I** – Acidic Wash		
	**3** (2 x 5.0 equiv.)	**IIa** – Glycosylation with thioglycoside – 2 cycles	-40 °C (T_1)<br>-10 °C (T_2)	10 min (t_1)<br>50 min (t_2)
		**III** – Capping<br>**IVc** – Fmoc Deprotection - 2 cycles		
1x		**I** – Acidic Wash		
	**2** (2 x 5.0 equiv.)	**IIa** – Glycosylation with thioglycoside – 2 cycles	-40 °C (T_1)<br>-20 °C (T_2)	30 min (t_1)<br>20 min (t_2)
		**III** – Capping<br>**IVc** – Fmoc Deprotection - 2 cycles		
1x		**I** – Acidic Wash		
	**4** (2 x 5.0 equiv.)	**IIa** – Glycosylation with thioglycoside – 2 cycles	-20 °C (T_1)<br>0 °C (T_2)	10 min (t_1)<br>30 min (t_2)

Protected **LPS-5** (16 mg, 0.011 mmol, crude yield: 79%) was obtained as a colorless oil after photocleavage from solid support following **Method A-1**. Deprotection of **LPS-5** following **Method C** and **D** and purification by reverse-phase HPLC (**Method E-1**, t_R = 17.4 min) afforded deprotected compound **LPS-5** (1.2 mg, 0.002 mmol, 15%) as a white solid after lyophylization.

^{1}H NMR (700 MHz, D_2O): δ 4.83 (d, *J* = 3.9 Hz, 1H), 4.80 (s, 1H), 4.65 (d, *J* = 8.5 Hz, 1H), 4.11 (d, *J* = 3.4 Hz, 1H), 3.96 (dd, *J* = 11.0, 8.5 Hz, 1H), 3.89 – 3.56 (m, 14H), 3.46 (dt, *J* = 10.1, 6.3 Hz, 1H), 3.36 (t, *J* = 9.7 Hz, 1H), 2.93 (t, *J* = 7.6 Hz, 2H), 1.97 (s, 3H), 1.61 (dt, *J* = 12.3, 6.1 Hz, 4H), 1.38 (q, *J* = 8.7 Hz, 2H), 1.20 (d, *J* = 6.2 Hz, 3H) ppm.

^{13}C NMR (176 MHz, D_2O): δ 174.9, 102.5, 102.3, 98.4, 79.0, 78.6, 75.0, 71.9, 70.5, 70.4, 70.0, 69.3, 67.8, 67.6, 67.3, 61.2, 60.9, 51.8, 39.4, 28.1, 26.5, 22.4, 22.2, 16.6 ppm.

HRMS (QToF): Calcd for $C_{25}H_{47}N_2O_{15}$ [M + H]$^+$ 615.2971; found 615.2974.

RP-HPLC of crude deprotected **LPS-5** (ELSD trace, **Method E-1**, t_R = 17.4 min):

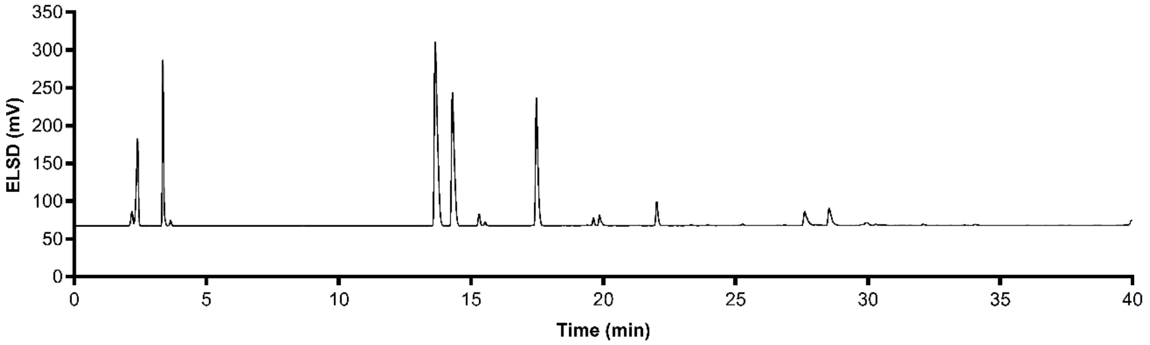

RP-HPLC of purified deprotected **LPS-5** (ELSD trace, **Method E-1**, t_R = 17.4 min):

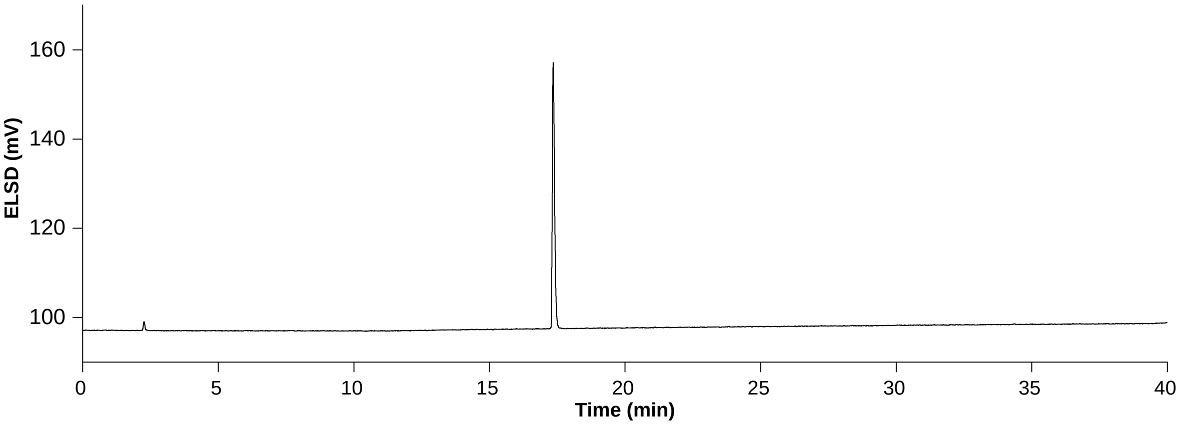

^{1}H NMR (700 MHz, D_2O) of **LPS-5**:

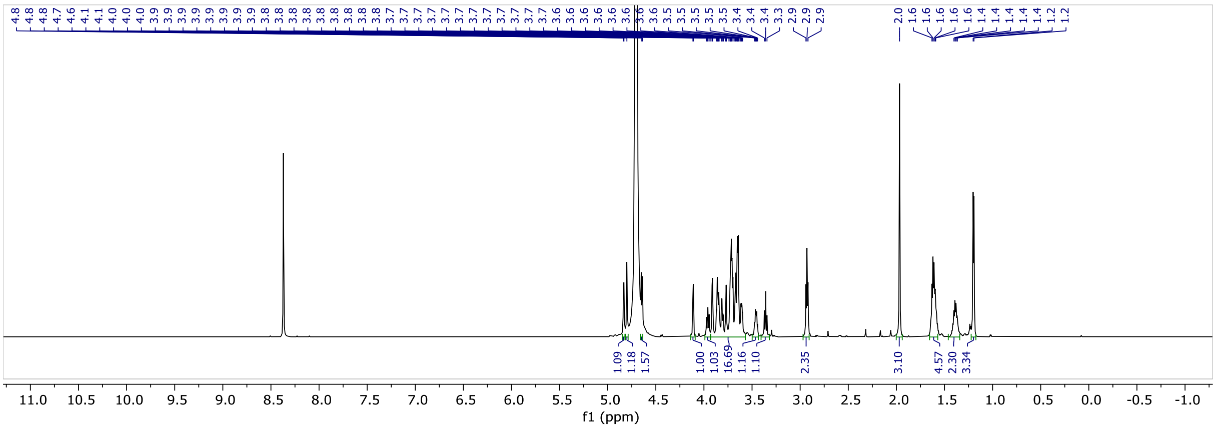

^{13}C NMR (176 MHz, D_2O) of **LPS-5**:

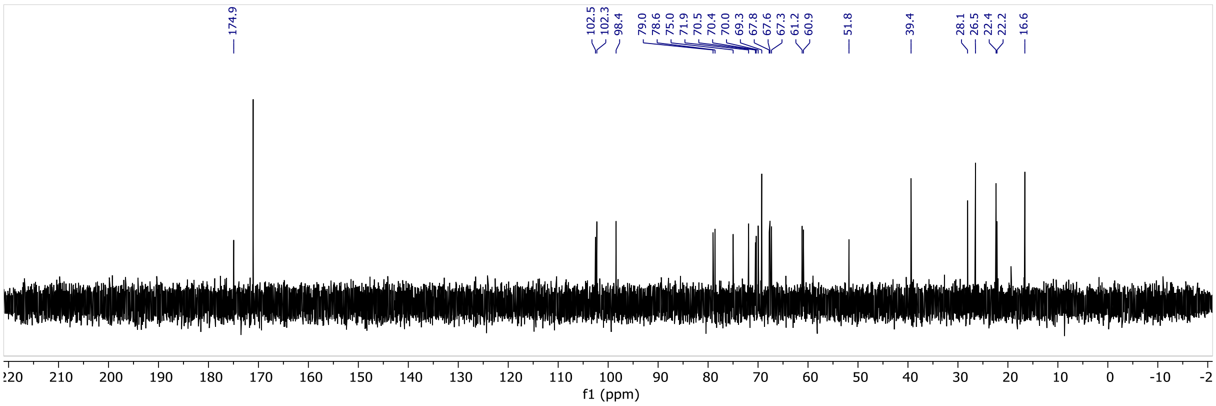

Coupled ^{13}C,^{1}H HSQC of **LPS-5**:

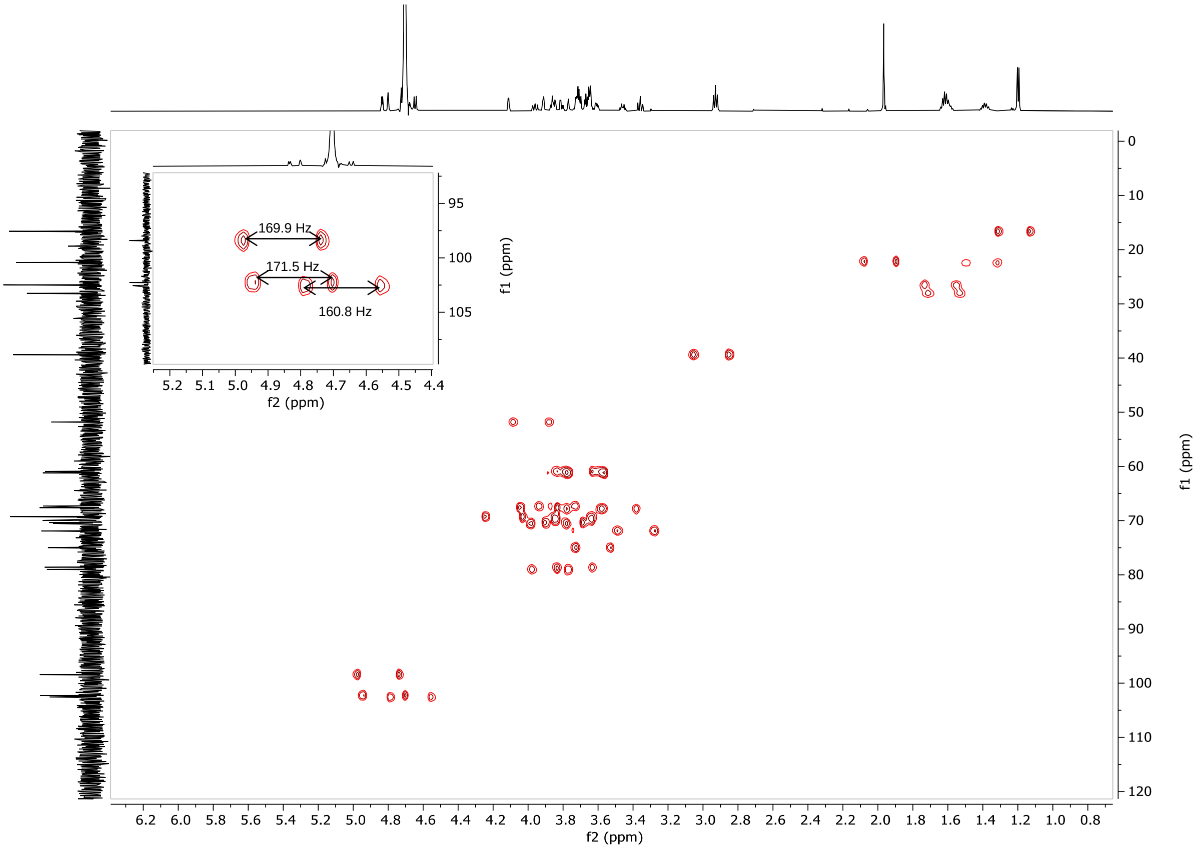

$^{13}C,^{1}H$ HSQC of **LPS-5**:

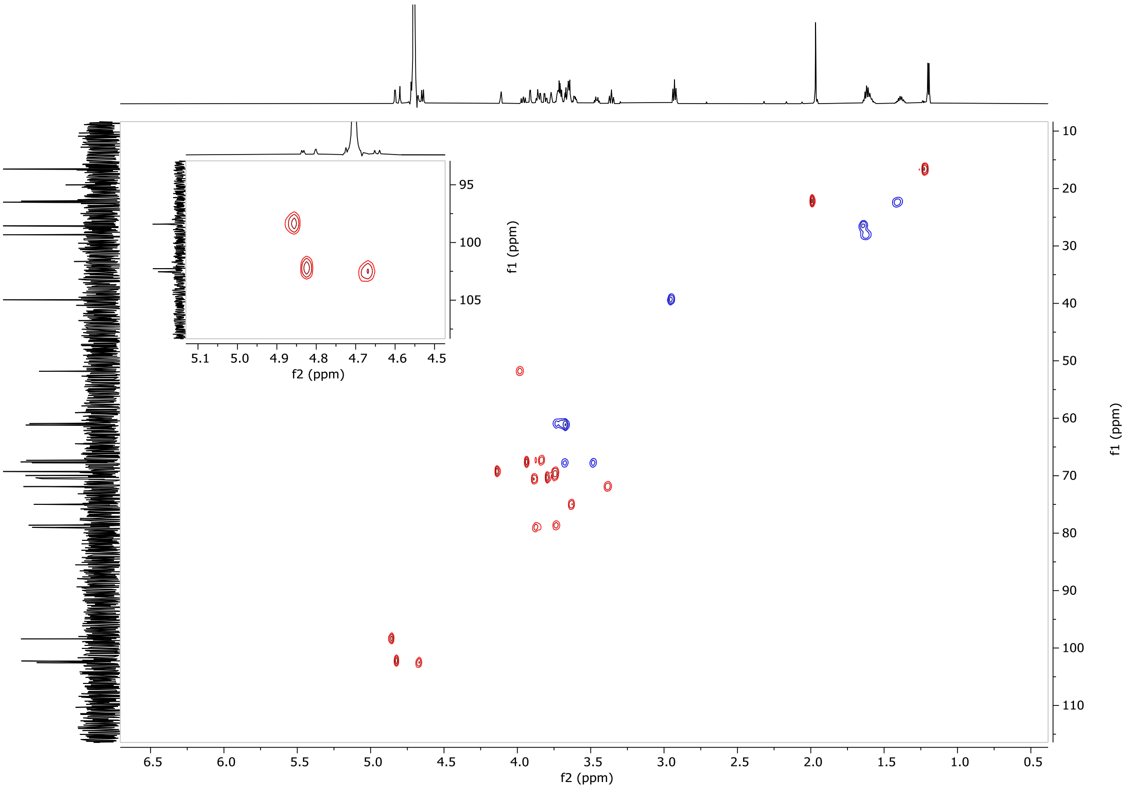

5-Amino-pentyl β-D-galactopyranosyl-(1→3)-α-D-galactopyranosyl-(1→6)-α-D-glucopyranoside (LPS-7)

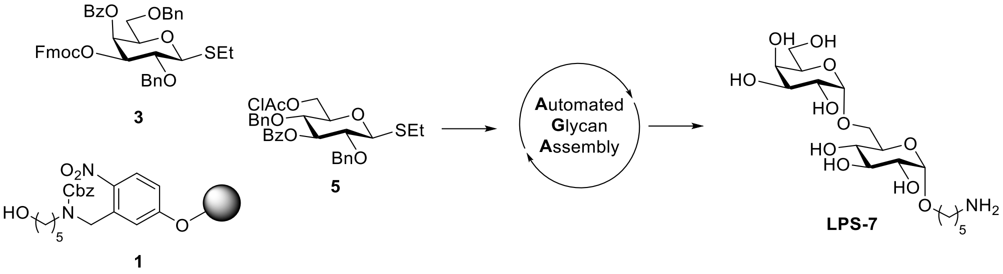

Repeat	Building Blocks	Modules	Notes	
1x		**I** – Acidic Wash		
	**5** (2 x 5.0 equiv.)	**IIa** – Glycosylation with thioglycoside – 2 cycles	-20 °C (T_1)<br>0 °C (T_2)	5 min (t_1)<br>30 min (t_2)
		**III** – Capping<br>**IVe** – ClAc Deprotection		
1x		**I** – Acidic Wash		
	**3** (2 x 5.0 equiv.)	**IIa** – Glycosylation with thioglycoside – 2 cycles	-40 °C (T_1)<br>-10 °C (T_2)	10 min (t_1)<br>50 min (t_2)
		**III** – Capping<br>**IVc** – Fmoc Deprotection		

Protected **LPS-7** (12 mg, 0.010 mmol, crude yield: 76%) was obtained as a colorless oil after photocleavage from solid support following **Method A-1**. Deprotection of **LPS-7** following **Method C** and **D** and purification by reverse-phase HPLC (**Method E-1**, t_R = 17.4 min) afforded deprotected compound **LPS-7** (0.56 mg, 0.013 mmol, 10%) as a white solid after lyophylization.

^{1}H NMR (700 MHz, D_2O): δ 4.93 (d, *J* = 3.7 Hz, 1H), 4.88 (d, *J* = 3.8 Hz, 1H), 3.96 – 3.91 (m, 3H), 3.85 – 3.76 (m, 3H), 3.73 – 3.66 (m, 4H), 3.64 (t, *J* = 9.4 Hz, 1H), 3.55 – 3.44 (m, 3H), 2.96 (t, *J* = 7.6 Hz, 2H), 1.69 – 1.60 (m, 4H), 1.48 – 1.36 (m, 2H).

^{13}C NMR (176 MHz, D_2O): δ 98.0, 97.9, 73.3, 71.1, 70.9, 70.2, 69.3, 69.1, 68.3, 67.8, 65.5, 61.0, 39.3, 28.0, 26.5, 22.4.

HRMS (QToF): Calcd for $C_{17}H_{34}NO_{11}$ $[M + H]^+$ 428.2126; found 428.2142.

^{1}H NMR (700 MHz, D_2O) of **LPS-7**:

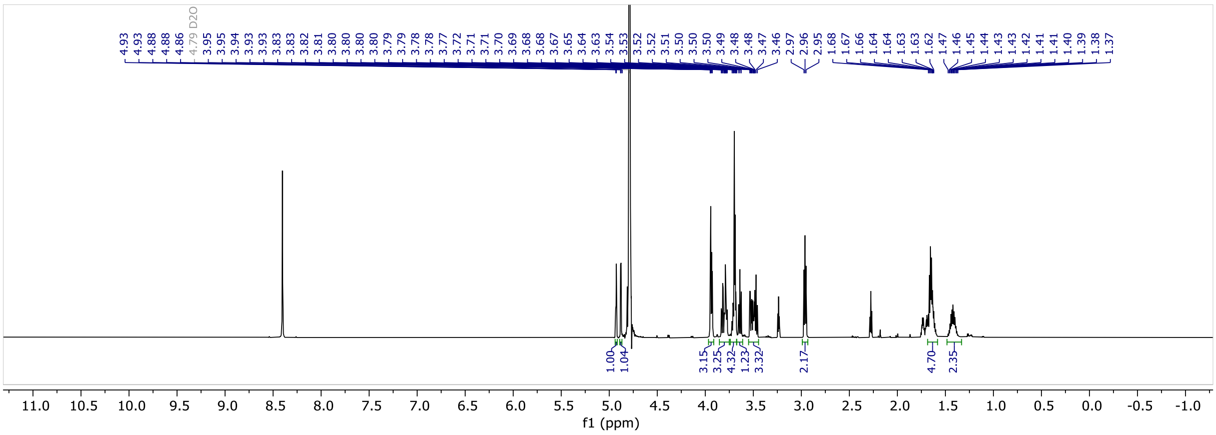

^{13}C NMR (176 MHz, D_2O) of **LPS-7**:

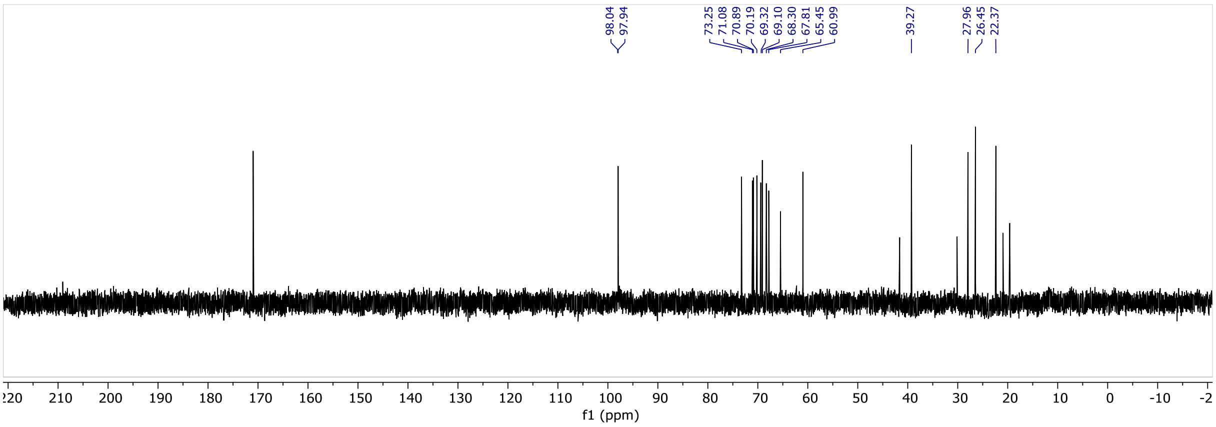

Coupled $^{13}C,^{1}H$ HSQC of **LPS-7**:

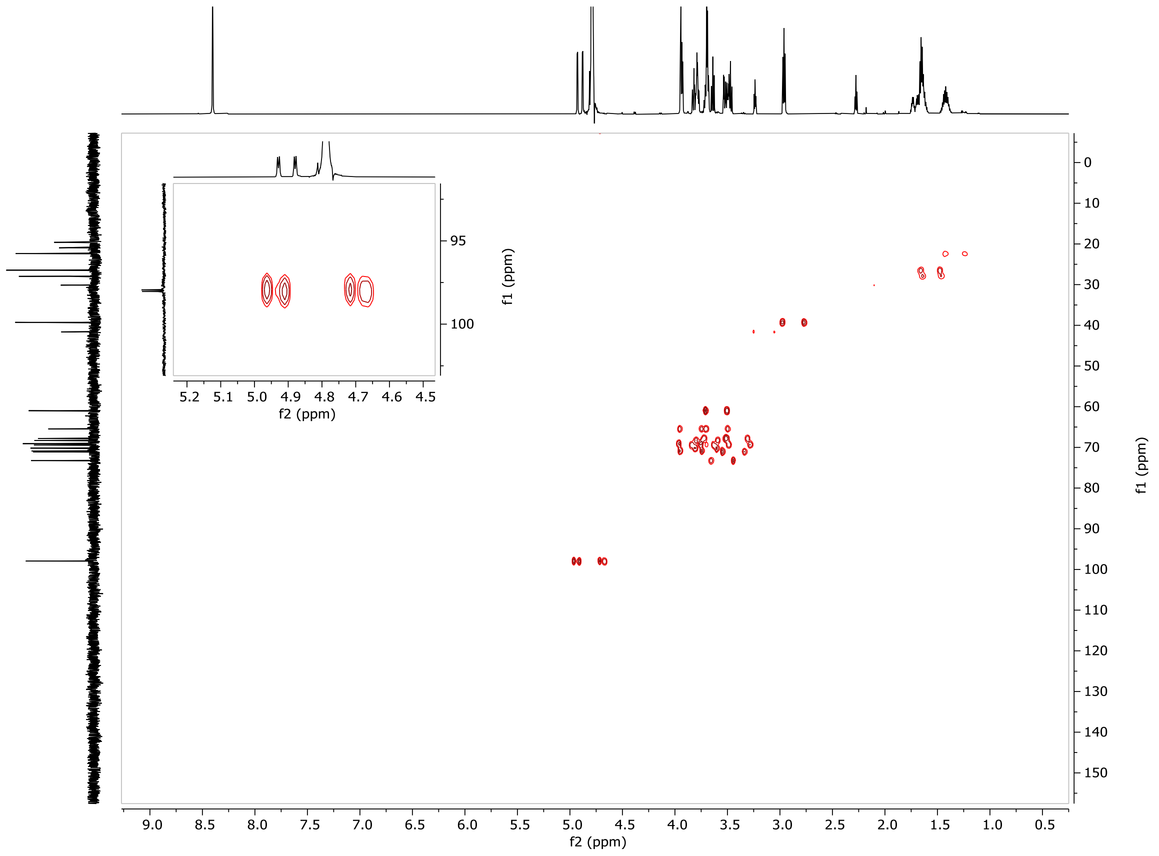

$^{13}C,^{1}H$ HSQC of **LPS-7**:

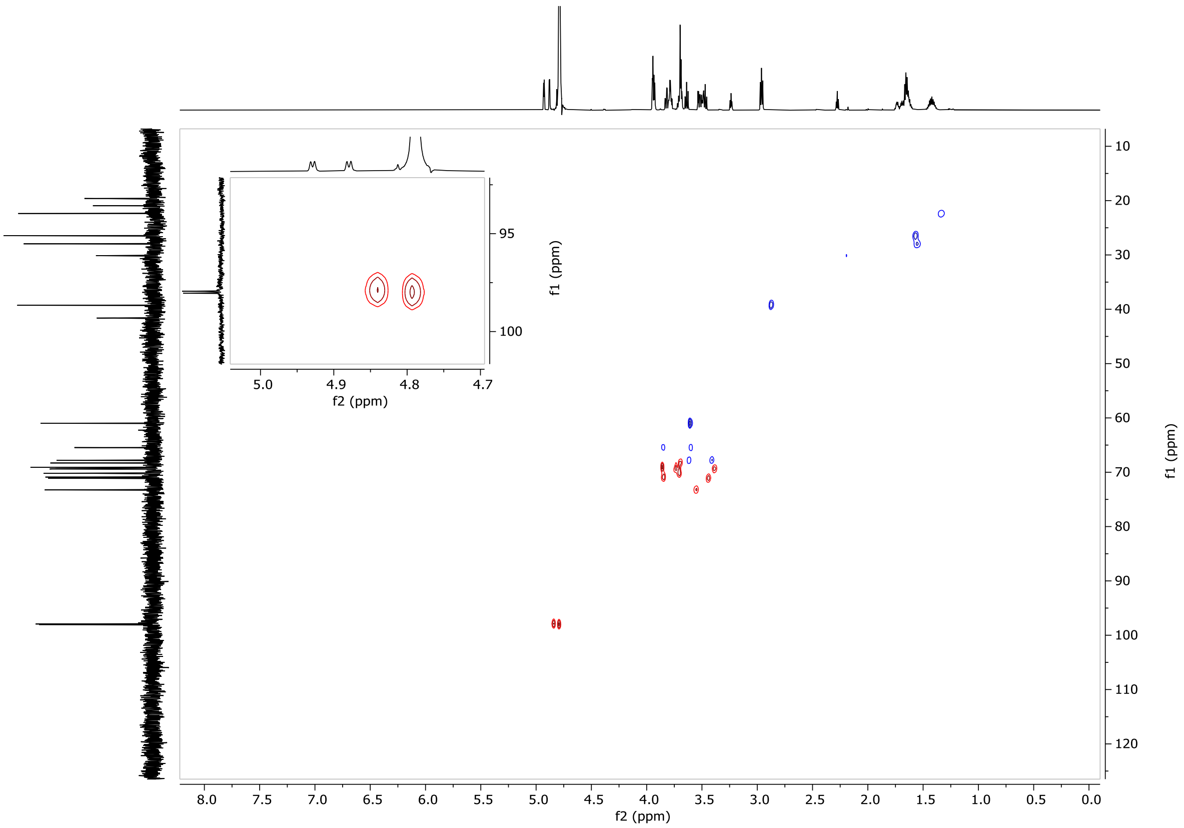

5-Amino-pentyl α-(1→2)-L-trirhamnopyranoside (6)

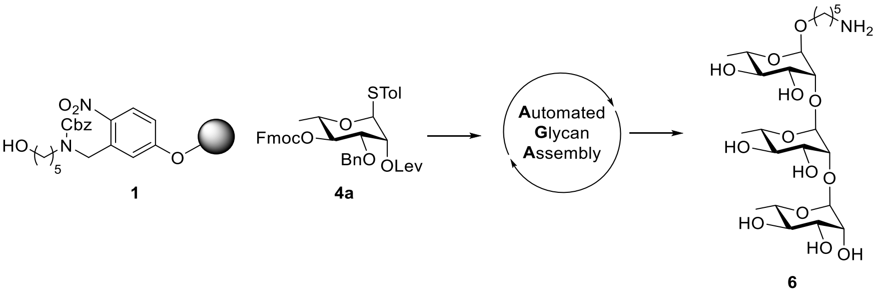

Repeat	Building Blocks	Modules	Notes	
**3x**		**I** – Acidic Wash		
	**4a** (3 x 3.0 equiv.)	**IIa** – Glycosylation with thioglycoside – 2 cycles	-20 °C (T_1)<br>0 °C (T_2)	10 min (t_1)<br>30 min (t_2)
		**III** – Capping<br>**IVb** – Lev Deprotection		

Protected **6** (35 mg, 0.022 mmol, crude yield: 80%) was obtained as a colorless oil after photocleavage from solid support following **Method A-1**. Deprotection of **6** following **Method C** and **D** and purification by reverse-phase HPLC (**Method E-1**, t_R = 17.4 min) afforded deprotected compound **6** (6 mg, 0.011 mmol, 41%) as a white solid after lyophylization.

^{1}H NMR (700 MHz, D_2O): δ 5.12 (d, *J* = 1.8 Hz, 1H), 4.98 (d, *J* = 1.8 Hz, 1H), 4.88 (d, *J* = 1.7 Hz, 1H), 4.09 (ddd, *J* = 11.5, 3.4, 1.7 Hz, 2H), 3.95 – 3.88 (m, 2H), 3.86 (dd, *J* = 9.8, 3.4 Hz, 1H), 3.82 – 3.67 (m, 5H), 3.56 (dt, *J* = 10.0, 6.1 Hz, 1H), 3.53 – 3.40 (m, 3H), 3.01 (t, *J* = 7.6 Hz, 2H), 1.76 – 1.61 (m, 4H), 1.53 – 1.41 (m, 2H), 1.31 (d, *J* = 6.3 Hz, 3H), 1.30 (d, *J* = 6.2 Hz, 3H), 1.28 (d, *J* = 6.2 Hz, 3H) ppm.

^{13}C NMR (176 MHz, D_2O): δ 102.2, 100.9, 98.4, 72.1, 72.1, 72.0, 70.2, 70.1, 70.0, 69.8, 69.3, 69.1, 68.8, 67.7, 39.4, 28.0, 26.6, 22.4, 16.6 ppm.

HRMS (QToF): Calcd for $C_{23}H_{44}NO_{13}$ $[M + H]^+$ 542.2807; found 542.2811.

^{1}H NMR (700 MHz, D_2O) of **6**:

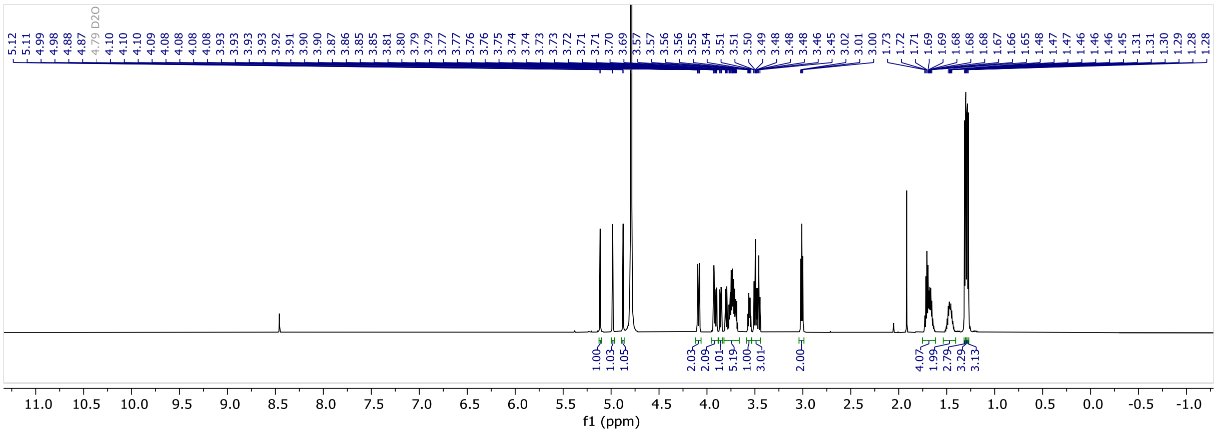

^{13}C NMR (176 MHz, D_2O) of **6**:

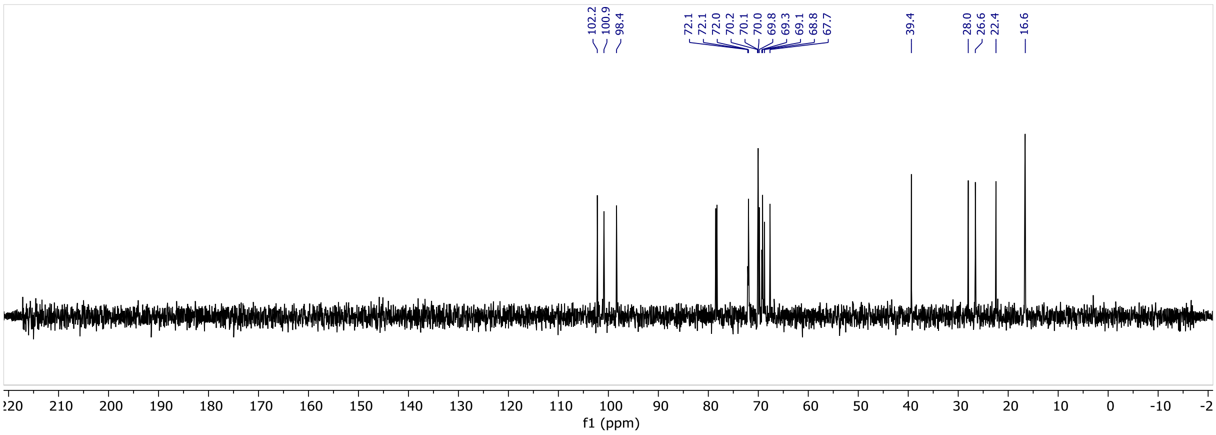

Coupled ^{13}C,^{1}H HSQC of **6**:

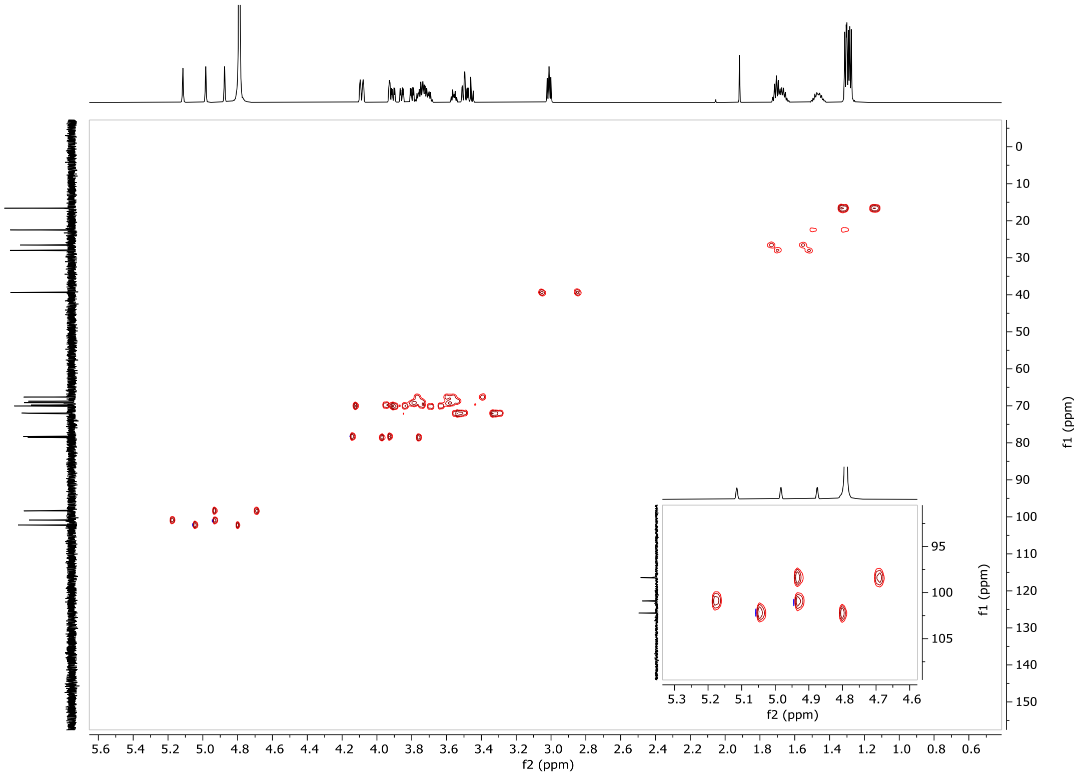

4 Preparation of Building Blocks

4.1 Rhamnose

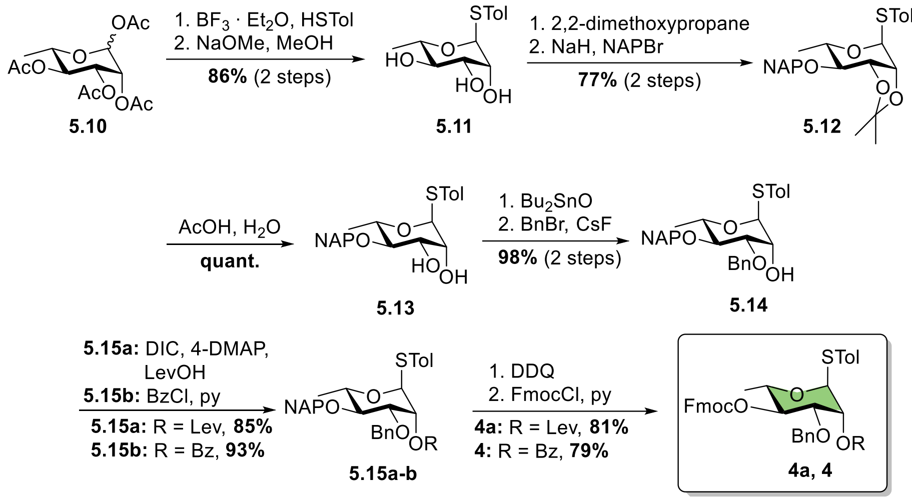

Scheme 1. Synthetic route to rhamnose building block **4a** and **4**.

4-Methylphenyl 2,3-*O*-(1-methylethylidene)-4-*O*-(2-naphthalenylmethyl)-1-thio-α-L-rhamnopyranoside (5.12)

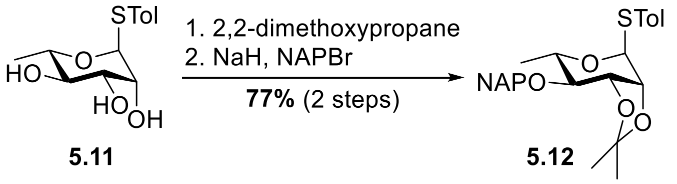

Rhamnopyranose **5.11**[3] (4.13 g, 15.3 mmol, 1.0 equiv.) was dissolved in anhydr. acetone (60 mL). 2,2,-Dimethoxypropane (7.5 mL, 61.2 mmol, 4.0 equiv.) was added followed by para-toluenesulfonic acid (582 mg, 3.1 mmol, 0.2 equiv.). The reaction mixture was stirred for 6 h at room temperature. Then, it was quenched by the addition of sat. aq. $NaHCO_3$, filtered and concentrated under reduced pressure. The residue was extracted with CH_2Cl_2 (2 x 100 mL), dried over Na_2SO_4, filtered and concentrated. 2,3-Isopropylidene product (3.7 g, 11.9 mmol, 78%) was obtained as a colorless solid after purification by column chromatography (Hex:AcOEt 3:1). R_f = 0.48 (Hex:AcOEt, 3:1). The residue (3.7 g, 11.9 mmol, 1.0 equiv.) was dissolved in dry *N,N*-dimethylformamide (40 mL). The stirred solution was cooled to 0 °C and sodium hydride (1.1 g, 27.4 mmol; 60% dispersion in mineral oil, 2.3 eq.) was added in small portions. After 30 min, NAPBr (4.0 g, 17.9 mmol, 1.5 equiv.) was added. The reaction mixture was allowed to warm up to room temperature and was stirred over night. Methanol (10 mL) was added, the reaction mixture was stirred for 10 min and afterwards diluted with ethyl acetate (100 mL). The organic layer was washed with water (2 x 100 mL). The aqueous phase was

extracted with ethyl acetate (2 x 100 mL). The combined organic phase was washed with water (100 mL), dried over Na_2SO_4 and concentrated. Product **5.12** (4.0 g, 8.9 mmol, **75%**) was obtained as a colorless oil after purification by column chromatography (Hex:AcOEt 9:1 to 3:1).

R*f* = 0.8 (Hex:AcOEt, 1:1).

^{1}H NMR (400 MHz, $CDCl_3$) δ 7.88 – 7.83 (m, 4H), 7.56 – 7.46 (m, 3H), 7.42 – 7.37 (m, 2H), 7.17 – 7.12 (m, 2H), 5.71 (s, 1H), 4.98 (dd, *J* = 108.5, 11.8 Hz, 2H), 4.41 – 4.39 (m, 1H), 4.23 (dd, *J* = 9.8, 6.2 Hz, 1H), 3.42 – 3.35 (m, 1H), 1.55 (s, 3H), 1.43 (s, 3H), 1.30 (d, *J* = 6.2 Hz, 3H) ppm.

^{13}C NMR (101 MHz, $CDCl_3$) δ 137.9, 135.8, 133.3, 133.1, 132.6, 129.9, 129.8, 128.2, 128.0, 127.8, 126.9, 126.2, 126.0, 109.5, 84.3, 81.5, 78.6, 76.8, 73.2, 66.2, 28.2, 26.6, 21.2, 17.9 ppm.

HRMS (QToF): Calcd for $C_{27}H_{30}O_4SNa$ $[M + Na]^+$ 473.1757; found 473.1743.

^{1}H NMR (400 MHz, $CDCl_3$) of **5.12**:

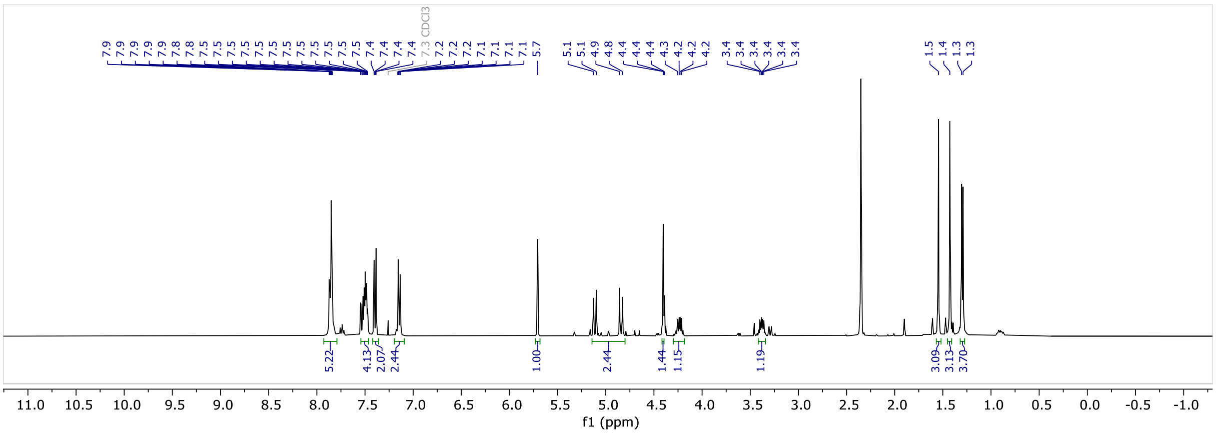

^{13}C NMR (101 MHz, $CDCl_3$) of **5.12**:

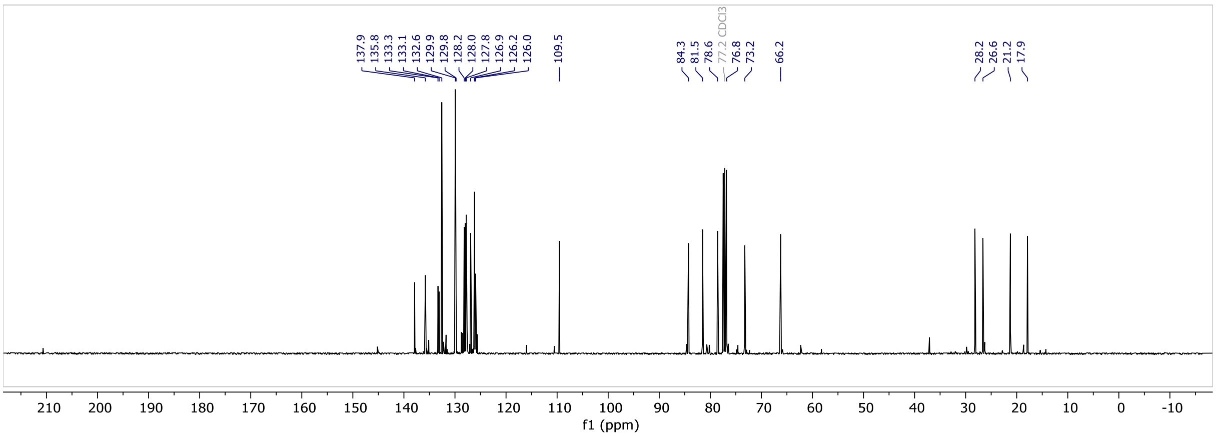

$^{13}C,^{1}H$ HSQC of **5.12**:

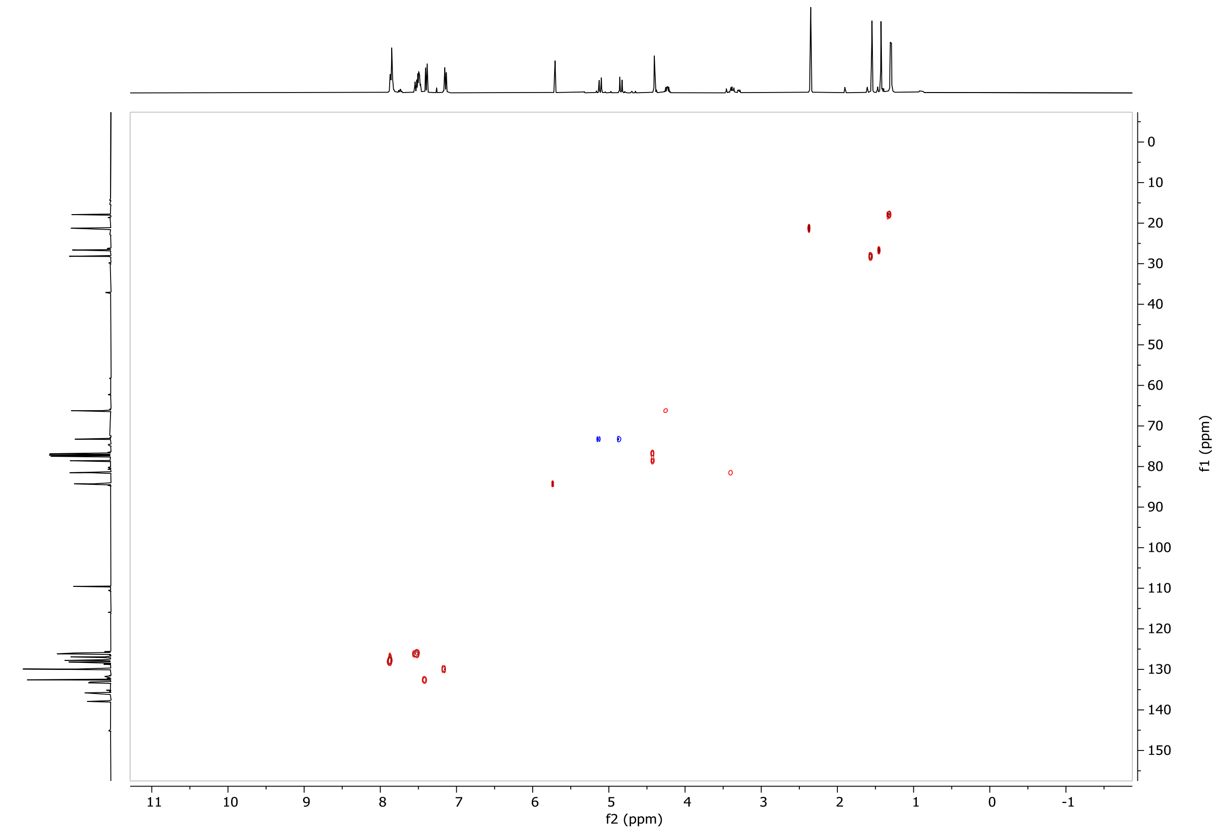

$^{1}H,^{1}H$ COSY of **5.12**:

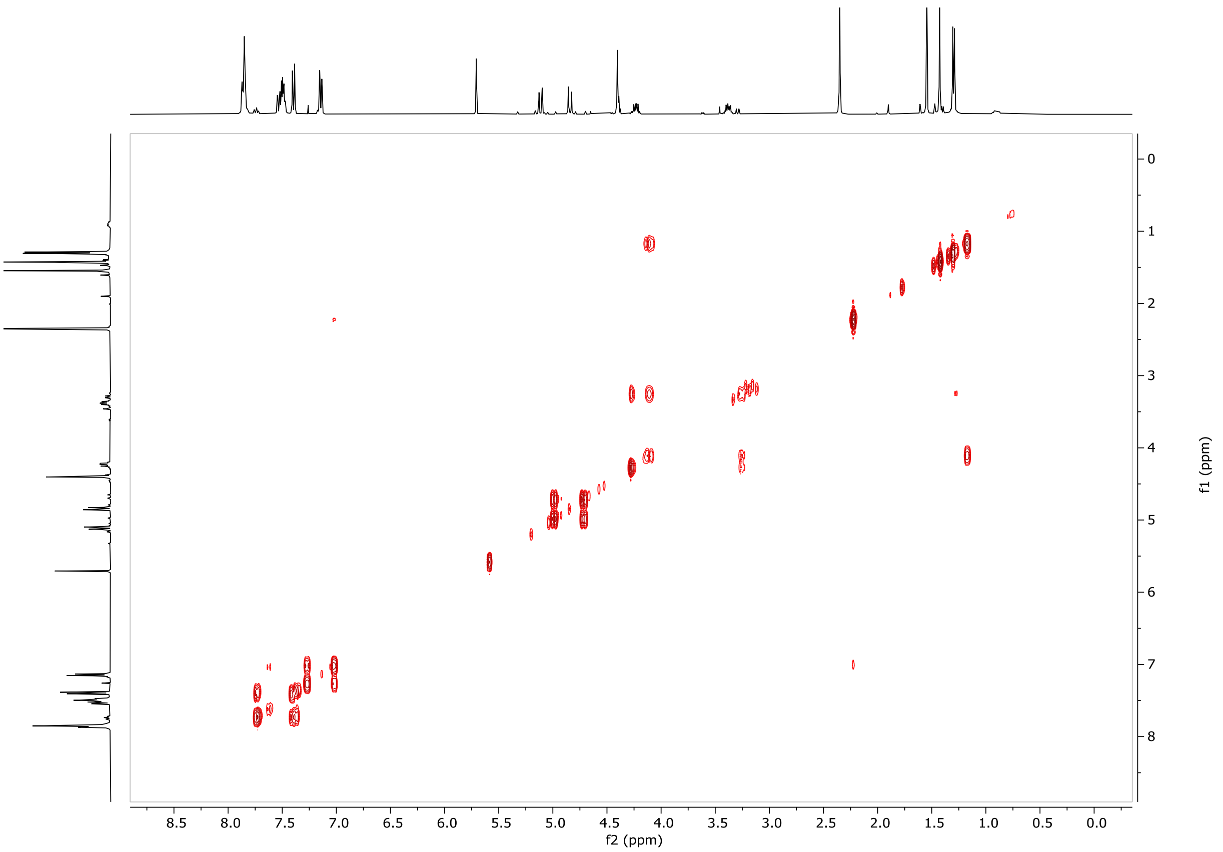

4-Methylphenyl 4-*O*-(2-naphthalenylmethyl)-1-thio-α-L-rhamnopyranoside (5.13)

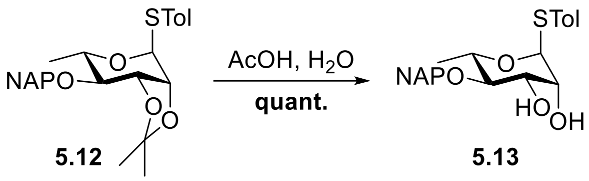

2,3-Isopropylidene **5.12** (4.0 g, 8.8 mmol, 1.0 equiv.) was dissolved in acetic acid/water (9:1, 50 mL) solution and reacted for 18 h at 50 °C. The mixture was concentrated and purified by column chromatography (Hex:AcOEt 3:1 to 1:1) to obtain title compound **5.13** (3.6 g, 1.7 mmol, 99%) as a colorless syrup.

R*f* = 0.27 (Hex:AcOEt, 1:1).

^{1}H NMR (400 MHz, $CDCl_3$) δ 7.90 – 7.76 (m, 4H), 7.53 – 7.46 (m, 3H), 7.40 – 7.31 (m, 2H), 7.12 (dd, *J* = 8.2, 4.1 Hz, 2H), 5.41 (d, *J* = 1.7 Hz, 1H), 4.92 (d, *J* = 3.2 Hz, 2H), 4.32 – 4.23 (m, 1H), 4.19 (dd, *J* = 3.4, 1.6 Hz, 1H), 3.97 (dd, *J* = 9.1, 3.4 Hz, 1H), 3.47 (t, *J* = 9.2 Hz, 1H), 2.33 (s, 3H), 1.39 (d, *J* = 6.2 Hz, 3H) ppm.

^{13}C NMR (101 MHz, $CDCl_3$) δ 137.9, 135.7, 133.4, 133.2, 132.3, 130.3, 130.0, 129.9, 128.7, 128.1, 127.9, 126.9, 126.4, 126.3, 125.9, 87.9, 82.0, 75.3, 72.7, 72.0, 68.7, 21.3, 18.1 ppm.

HRMS (QToF): Calcd for $C_{24}H_{26}O_4SNa$ $[M + Na]^+$ 433.1444; found 433.1432.

^{1}H NMR (400 MHz, $CDCl_3$) of **5.13**:

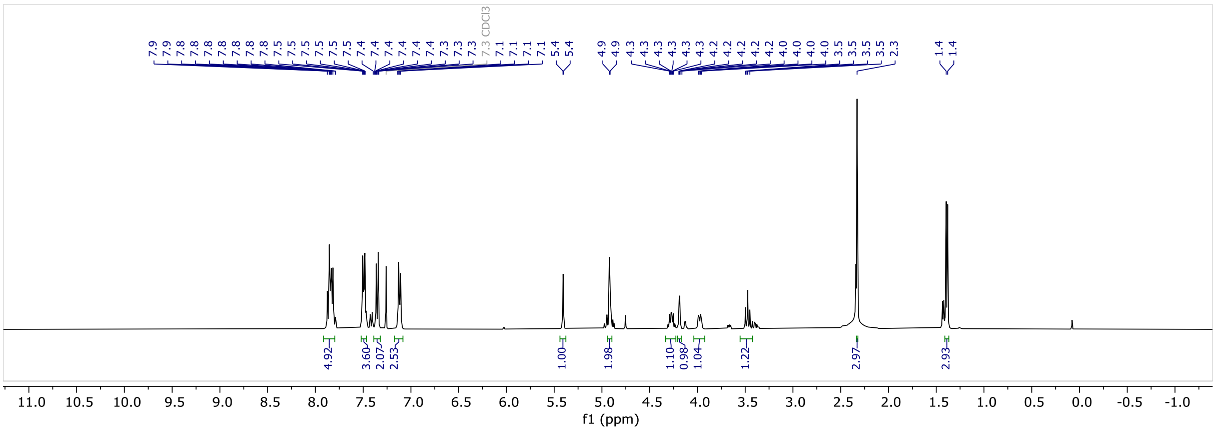

^{13}C NMR (101 MHz, $CDCl_3$) of **5.13**:

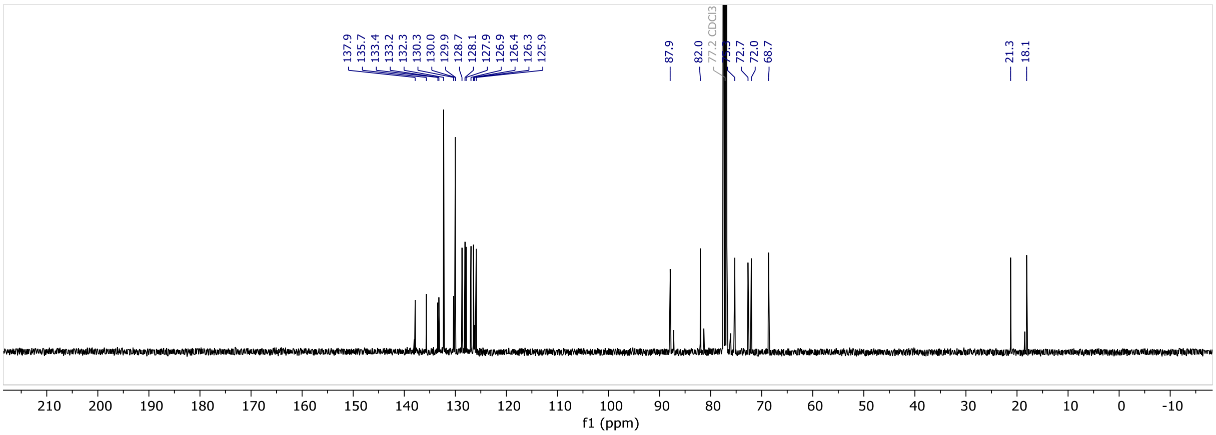

^{13}C,^{1}H HSQC of **5.13**:

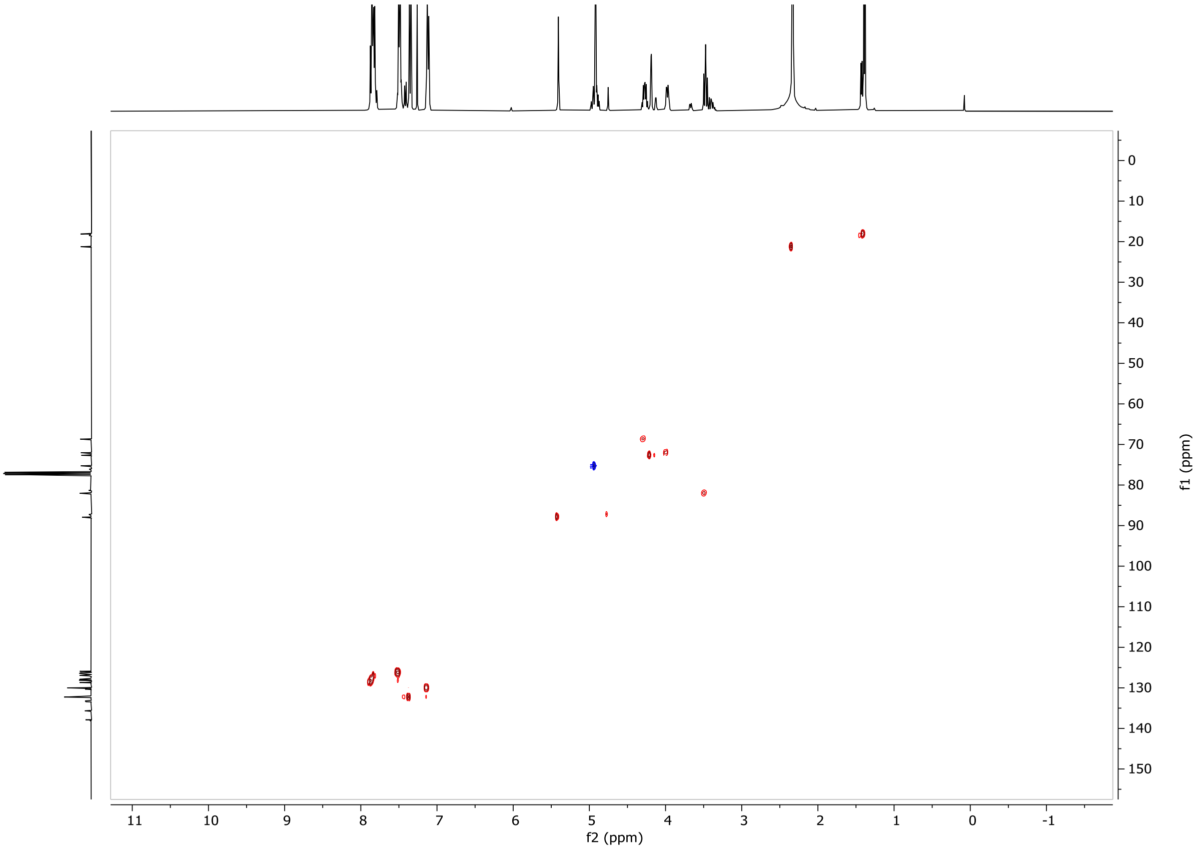

$^{1}H,^{1}H$ COSY of **5.13**:

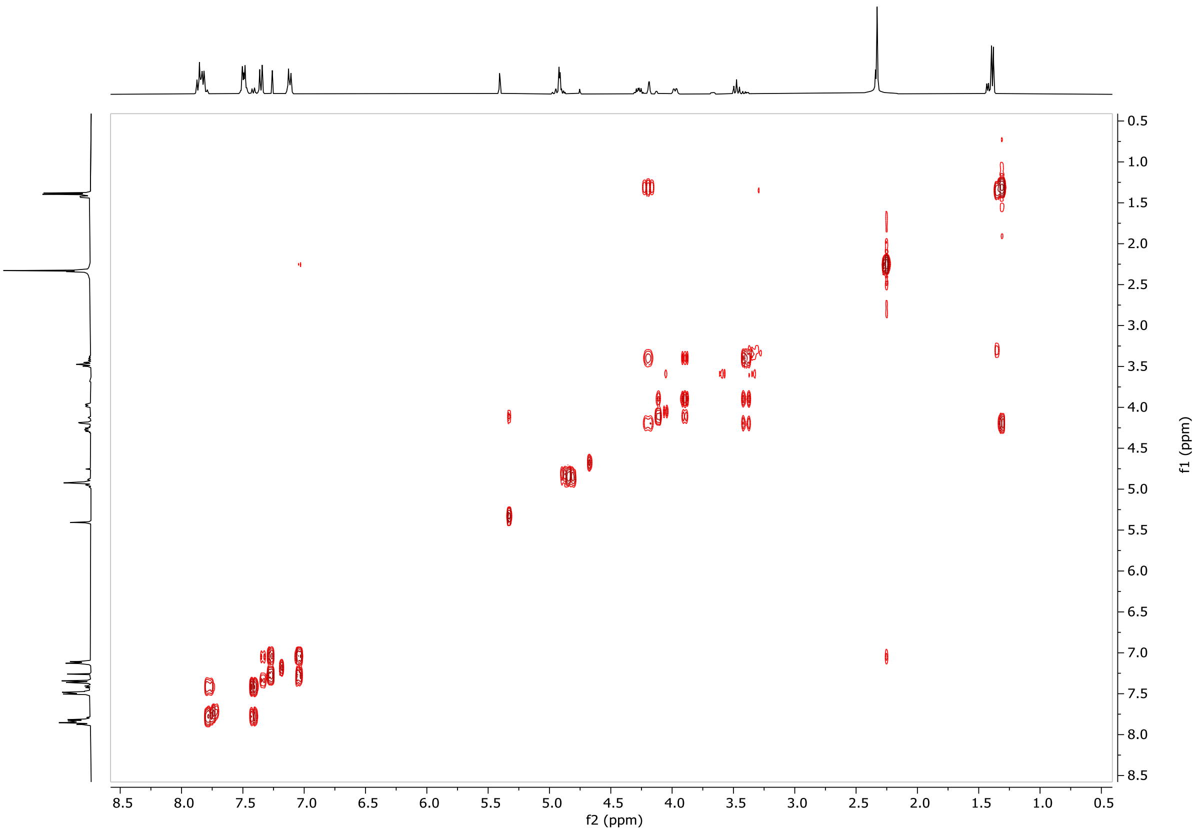

4-Methylphenyl 3-*O*-benzyl-4-*O*-(2-naphthalenylmethyl)-1-thio-α-L-rhamnopyranoside (5.14)

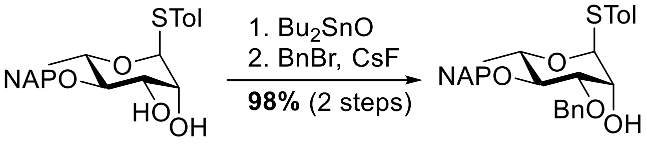

5.13 **5.14**

Diol **5.13** (7.0 g, 17.1 mmol, 1.0 equiv.) was dissolved in MeOH (100 mL) and Bu_2SnO (8.5 g, 34.2 mmol, 2.0 equiv.) was added. The reaction mixture was then heated up to 65 °C and stirred at the same temperature overnight. The reaction mixture was co-evaporated with toluene and dried under high vacuum. The crude was dissolved in DMF (100 mL) and benzyl bromide (2.45 mL, 20.5 mmol, 1.2 equiv.), and CsF (3.4 g, 22.2 mmol, 1.3 equiv.) were added to the stirred reaction mixture at room temperature under argon atmosphere. The reaction was stirred at room temperature for 5 h and then diluted with EtOAc, passed through a short plug of silica gel and dried under vacuum. The title compound **5.14** (8.4 g, 16.8 mmol, 98%) was obtained as a colorless oil after purification by column chromatography (Hex:AcOEt 9:1 to 3:1).

R_f = 0.41 (Hex:AcOEt, 3:1).

^{1}H NMR (400 MHz, $CDCl_3$) δ 7.86 – 7.76 (m, 4H), 7.53 – 7.43 (m, 3H), 7.41 – 7.32 (m, 7H), 7.12 (d, *J* = 8.2 Hz, 2H), 5.47 (d, *J* = 1.8 Hz, 1H), 4.94 (dd, *J* = 91.4, 11.2 Hz, 2H), 4.74 (s, 2H), 4.31 – 4.20 (m, 2H), 3.91 (dd, *J* = 9.0, 3.2 Hz, 1H), 3.59 (t, *J* = 9.3 Hz, 1H), 2.33 (s, 3H), 1.34 (d, *J* = 6.2 Hz, 3H) ppm.

^{13}C NMR (101 MHz, $CDCl_3$) δ 137.8, 137.7, 135.9, 133.4, 133.1, 132.2, 130.3, 130.0, 128.8, 128.3, 128.2, 128.1, 128.1, 127.8, 126.8, 126.2, 126.1, 126.0, 87.5, 80.3, 80.2, 75.6, 72.3, 70.2, 68.8, 21.3, 18.0 ppm.

HRMS (QToF): Calcd for $C_{31}H_{32}O_4SNa$ $[M + Na]^+$ 523.1913; found 523.1924.

^{1}H NMR (400 MHz, $CDCl_3$) of **5.14**:

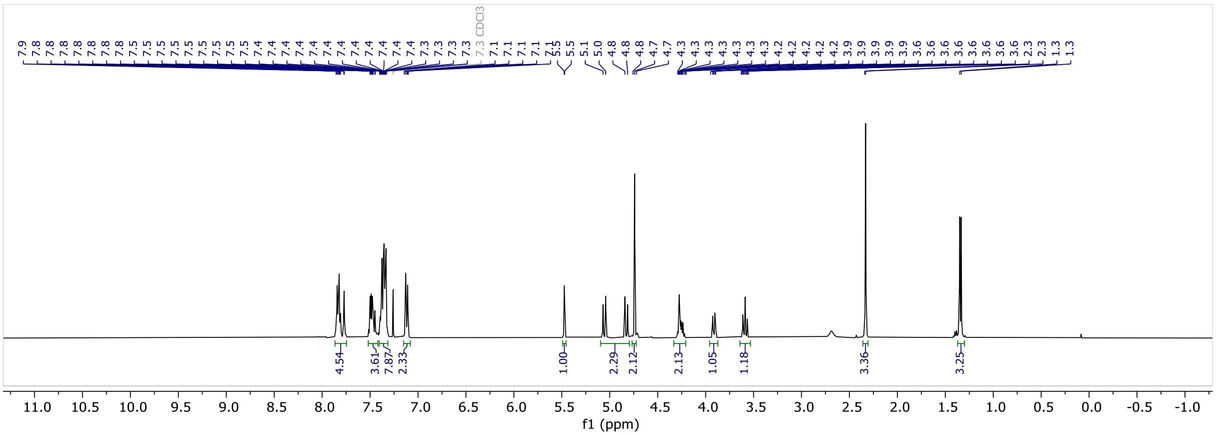

^{13}C NMR (101 MHz, $CDCl_3$) of **5.14**:

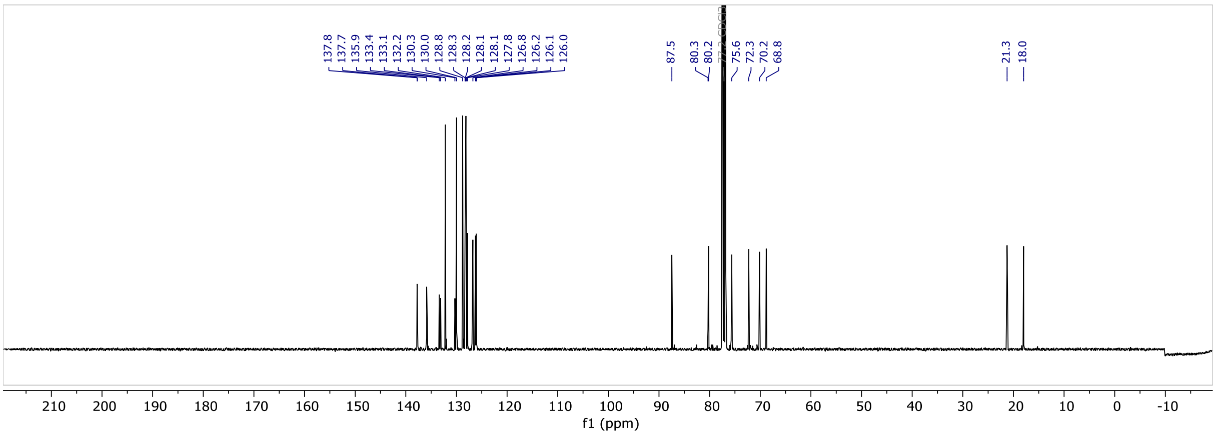

$^{13}C,^{1}H$ HSQC of **5.14**:

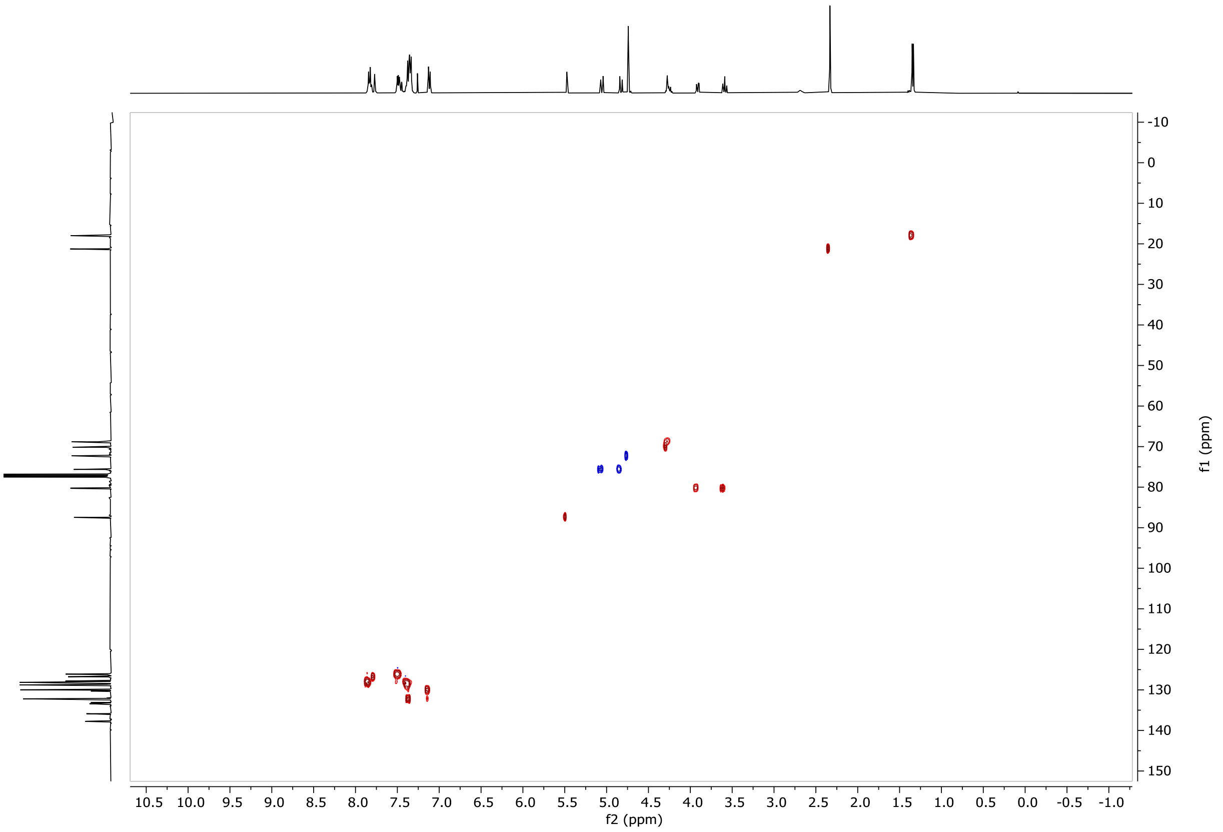

$^{1}H,^{1}H$ COSY of **5.14**:

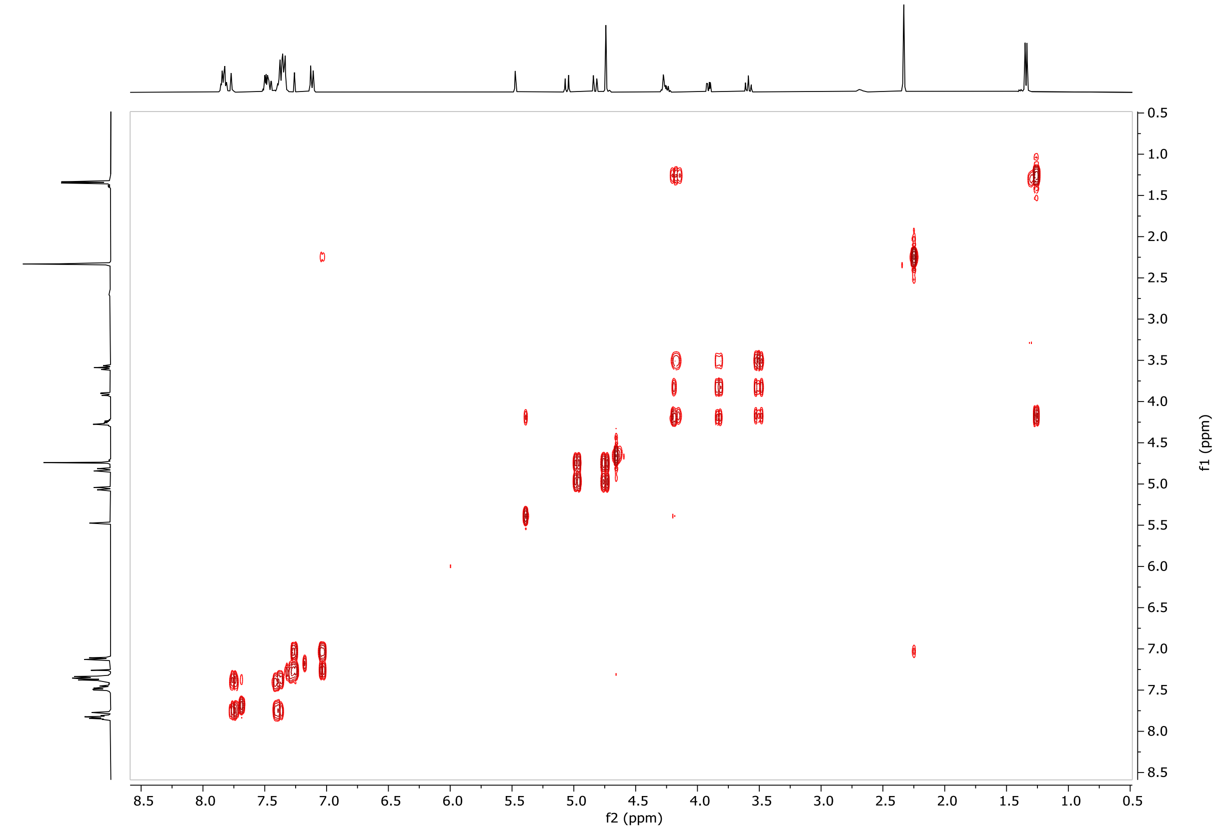

4-Methylphenyl 3-*O*-benzyl-2-*O*-levulinyl-4-*O*-fluorenylmethoxycarbonyl-1-thio-α-L-rhamnopyranoside (5.15a)

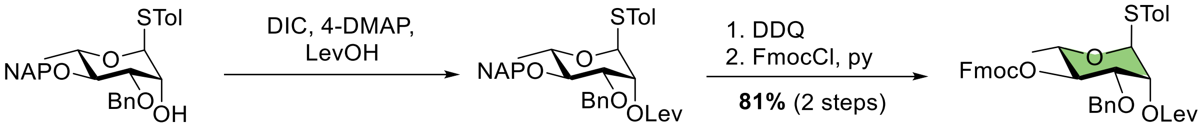

5.14 **5.15a** **4a**

To a solution of **5.14** (800 mg, 1.6 mmol, 1.0 equiv.) and LevOH (0.33 mL, 3.2 mmol, 2.0 equiv.) in anhydrous CH_2Cl_2 (15 mL) was added DIC (0.75 mL, 4.8 mmol, 3.0 equiv.) and 4-DMAP (39 mg, 0.32 mmol, 0.2 equiv.) at 0 °C. The reaction was stirred at room temperature overnight. The reaction mixture was filtered through Celite, the filtrate was washed with aqueous $NaHCO_3$ and the aqueous phase was extracted with CH_2Cl_2. The combined organic phase was dried over Na_2SO_4, filtered and concentrated. Sugar **5.15a** (810 mg, 1.35 mmol, 85%) was obtained as a colorless syrup after purification by column chromatography (Hex:AcOEt 3:1). **R*f*** = 0.35 (Hex: EtOAc 3:1). To a well stirred emulsion of sugar **5.15a** (810 mg, 1.3 mmol, 1.0 equiv.) in CH_2Cl_2/water (7:1, 24 mL), was added DDQ (337 mg, 1.5 mmol, 1.1 equiv.) and the suspension was stirred at room temperature for 1.5 h protected from light. The mixture was diluted with CH_2Cl_2, washed with 10% $Na_2S_2O_3$ and saturated aqueous $NaHCO_3$ solution. The organic layer was dried over Na_2SO_4, filtered, concentrated and the residue was purified by column chromatography (Hex:AcOEt 9:1 to 3:1) to obtain **3-OH-sugar** (620 mg, 1.4 mmol, quant.) as a colorless solid. **R*f*** = 0.35 (Hex:AcOEt 3:1). To a solution of **3-OH-sugar** (620 g, 1.4 mmol, 1.0 equiv.) and anhydrous pyridine (0.55 mL, 6.7 mmol, 5.0 equiv.) in anhydrous CH_2Cl_2 (15 mL) at 0 °C was added FmocCl (524 mg, 2.0 mmol, 1.5 equiv.) followed by 4-DMAP (8 mg, 0.1 mmol, 0.05 equiv.) was added and the reaction mixture was stirred for two hours at 0 °C. Aqueous citric acid solution (10 mL) was added and the mixture was allowed to warm up to room temperature. The aqueous phase was extracted with CH_2Cl_2 and the combined organic phase was dried over Na_2SO_4, filtered and concentrated. The title compound **4a** (840 mg, 1.2 mmol, 91%) was obtained as a white solid after purification by column chromatography (Hex:AcOEt = 19:1 to 3:1).

R*f* = 0.25 (Hex:AcOEt 3:1).

$[\alpha]_D$ -39.32 cm^{-1} (*c* 1, $CHCl_3$).

IR (film): 3067, 2872, 1749, 1723, 1603, 1495, 1451, 1386, 1246, 1152, 1102, 986, 848, 784, 738, 698 cm^{-1}.

HRMS (QToF): Calcd for $C_{40}H_{40}O_8SNa$ $[M + Na]^+$ 703.2336; found 703.2354.

^{1}H NMR (400 MHz, CDCl$_3$): δ 7.79 (d, *J* = 7.5 Hz, 2H), 7.67 – 7.57 (m, 2H), 7.46 – 7.36 (m, 2H), 7.35 – 7.30 (m, 4H), 7.26 – 7.20 (m, 5H), 7.12 (d, *J* = 7.9 Hz, 2H), 5.57 (dd, *J* = 3.3, 1.7 Hz, 1H), 5.35 (d, *J* = 1.6 Hz, 1H), 4.88 (t, *J* = 9.8 Hz, 1H), 4.65 (d, *J* = 11.9 Hz, 1H), 4.52 – 4.40 (m, 3H), 4.35 (dd, *J* = 9.8, 6.2 Hz, 1H), 4.26 (t, *J* = 7.2 Hz, 1H), 3.88 (dd, *J* = 9.7, 3.2 Hz, 1H), 2.68 (ddd, *J* = 10.8, 5.1, 1.3 Hz, 4H), 2.33 (s, 3H), 2.15 (s, 3H), 1.26 (d, *J* = 6.2 Hz, 3H) ppm.

^{13}C NMR (101 MHz, CDCl$_3$): δ 206.44, 171.98, 154.99, 143.51, 143.31, 141.45, 138.30, 137.41, 132.49, 130.09, 129.59, 128.50, 128.07, 127.99, 127.34, 125.23, 120.25, 86.32, 74.97, 71.56, 70.41, 70.13, 67.50, 46.94, 38.10, 29.93, 28.24, 21.29, 17.43 ppm.

^{1}H NMR (400 MHz, CDCl$_3$) of **4a**:

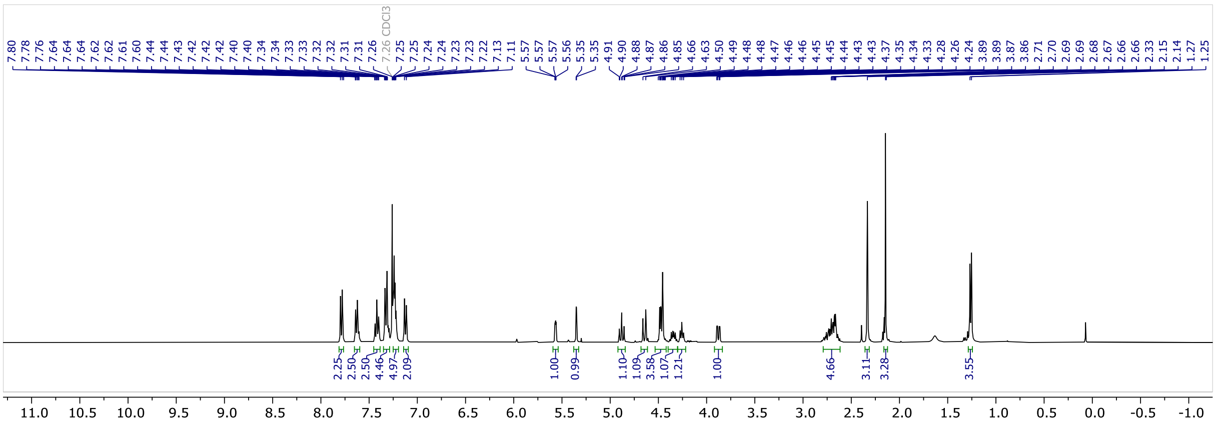

^{13}C NMR (101 MHz, CDCl$_3$) of **4a**:

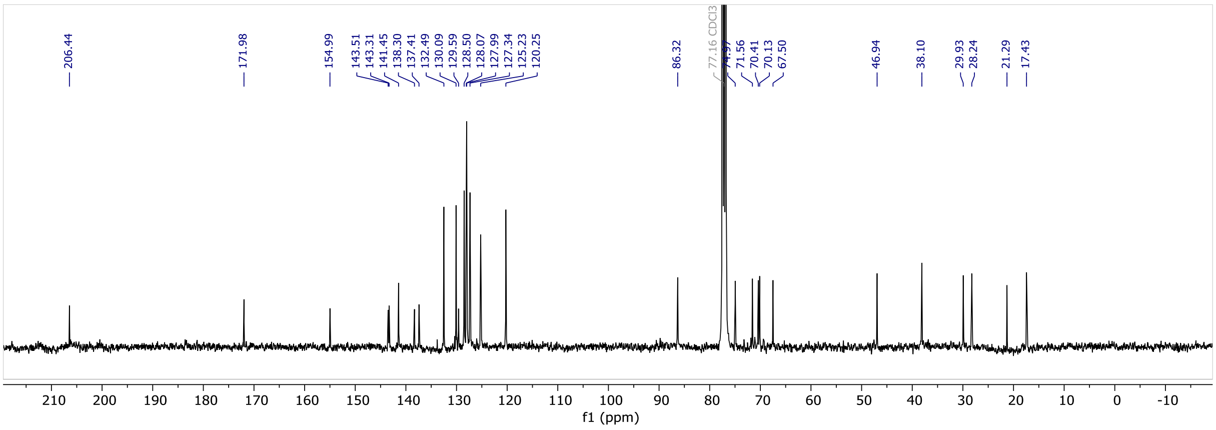

Coupled $^{13}C,^{1}H$ HSQC of **4a**:

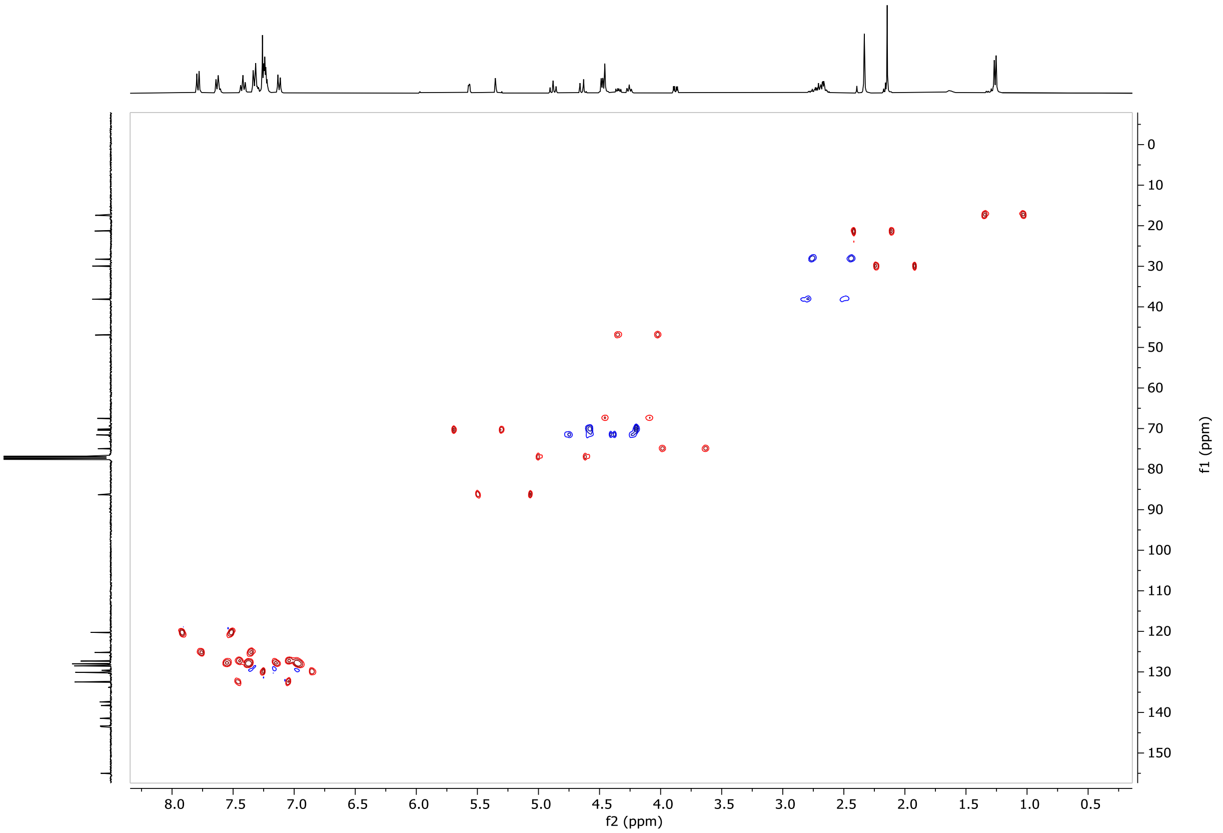

$^{13}C,^{1}H$ HSQC of **4a**:

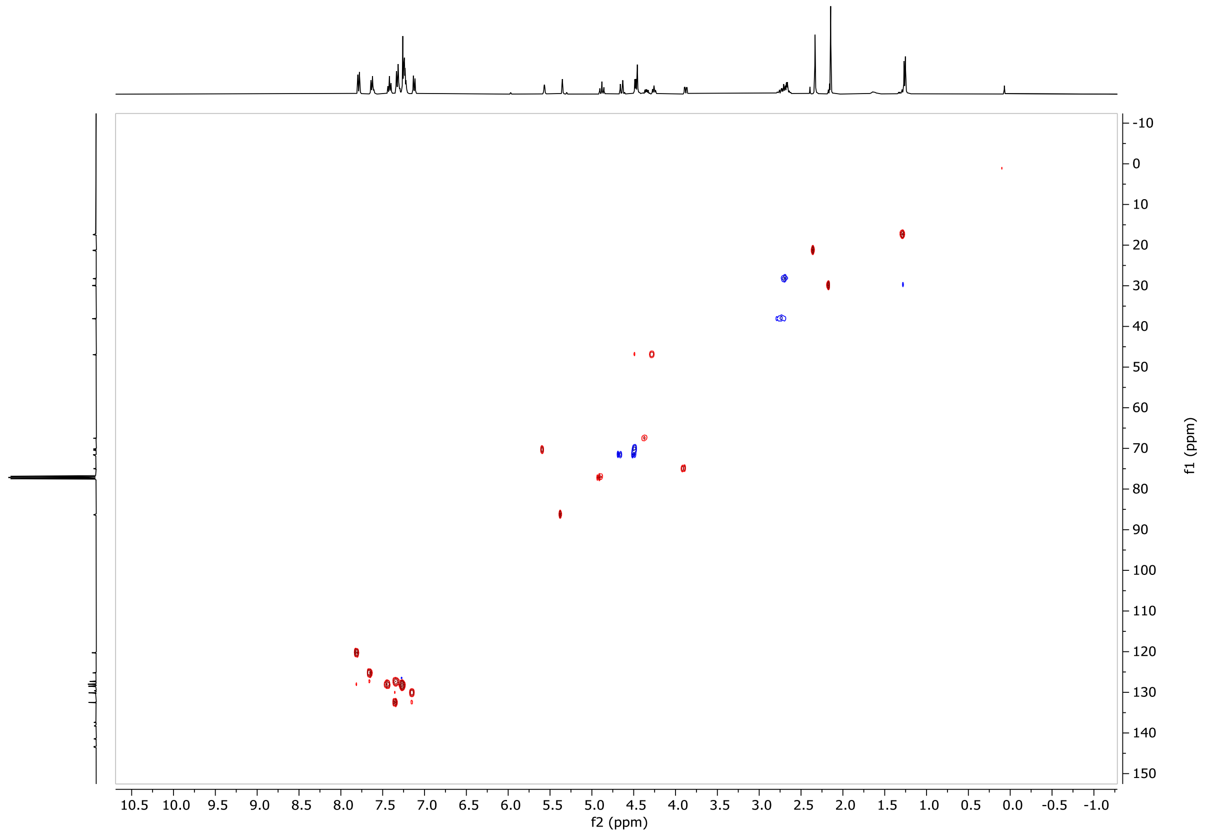

$^1H,^1H$ COSY of **4a**:

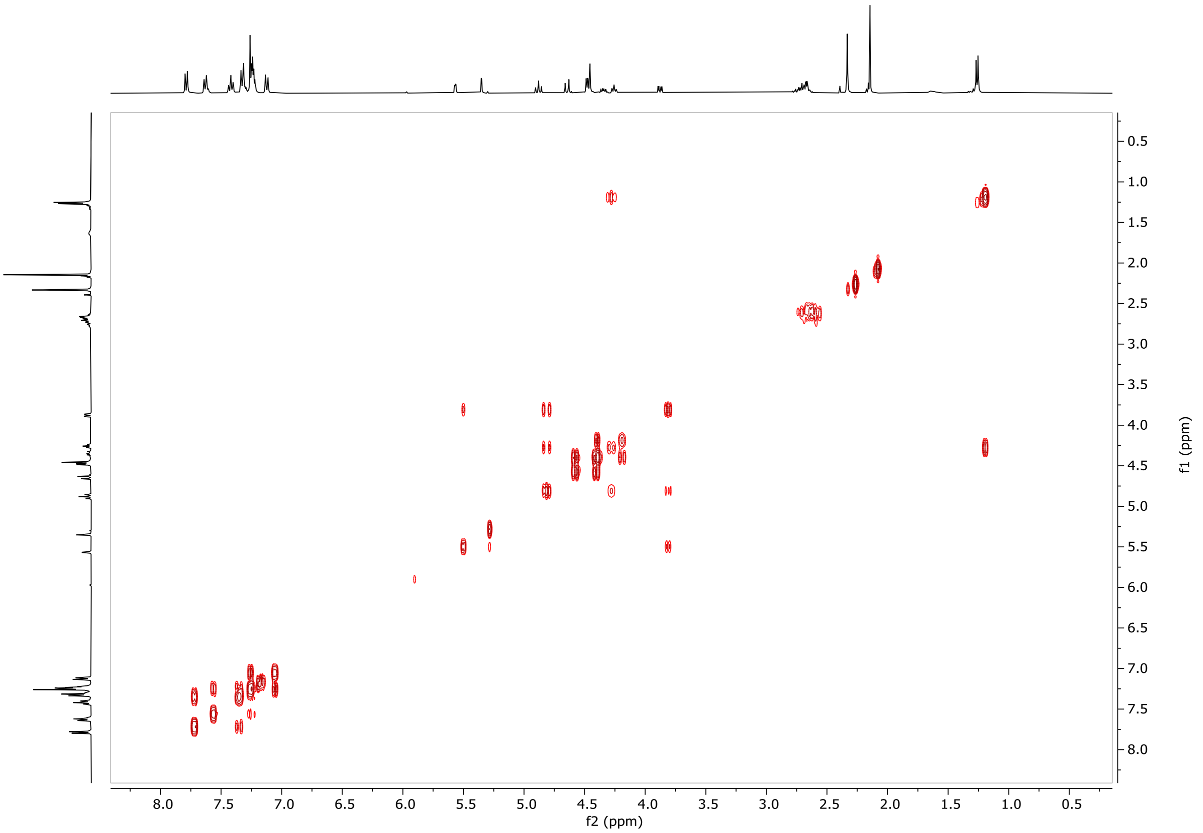

4-Methylphenyl 2-*O*-benzoyl 3-*O*-benzyl-4-*O*-fluorenylmethoxycarbonyl-1-thio-α-L-rhamnopyranoside (4)

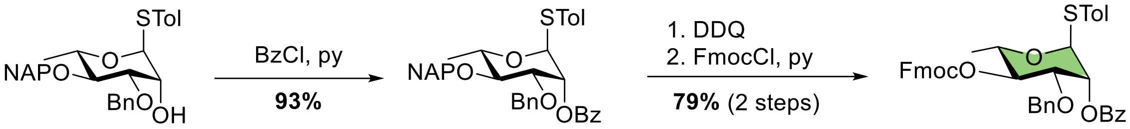

To a solution of **5.14** (5.0 g, 10.0 mmol, 1.0 equiv.) in anhydrous pyridine (10 mL), BzCl (3.48 mL, 30.0 mmol, 3.0 equiv.) was added at 0 °C under an argon atmosphere. The mixture was allowed to warm up to room temperature and stirred for 16 h. The reaction mixture was diluted with water and extracted with ethyl acetate. The combined organic phase was washed with aqueous citric acid solution (10% v/v) and brine, dried over Na_2SO_4, filtered and concentrated. The benzoylated **5.15b** (5.6 g, 9.3 mmol, 93%) was obtained as a colorless syrup after purification by column chromatography (Hex/EA 9:1). R_f = 0.41 (Hex: EtOAc 9:1). To a well stirred emulsion of **5.15b** (5.5 g, 9.1 mmol, 1.0 equiv.) in CH_2Cl_2/water (7:1, 48 mL), was added DDQ (1.3 g, 10.0 mmol, 1.1 equiv.) and the suspension was stirred at room temperature for 1.5 h protected from light. The mixture was diluted with CH_2Cl_2, washed with 10% aqueous $Na_2S_2O_3$ and saturated aqueous $NaHCO_3$ solution. The organic layer was dried

over Na_2SO_4, filtered, concentrated and the residue was purified by column chromatography (Hex/EA 9:1 to 3:1) to obtain the **4-OH-sugar** (3.8 g, 6.5 mmol, 71%) as a colorless solid. R_f = 0.41 (Hex:EA 3:1). To a solution of **4-OH-sugar** (3.8 g, 8.2 mmol, 1.0 equiv.) and anhydrous pyridine (3.31 mL, 40.9 mmol, 5.0 equiv.) in anhydrous CH_2Cl_2 (60 mL) at 0 °C was added FmocCl (3.2 g, 1.5 mmol, 1.5 equiv.) followed by 4-DMAP (50 mg, 0.4 mmol, 0.05 equiv.) was added and the reaction mixture was stirred for two hours at 0 °C. Aqueous citric acid solution (30 mL) was added and the mixture was allowed to warm up to room temperature. The aqueous phase was extracted with CH_2Cl_2 and the combined organic phase was dried over Na_2SO_4, filtered and concentrated. The title compound **4** (4.9 g, 7.1 mmol, 87%) was obtained as a white solid after purification by column chromatography (Hex/EtOAc = 19:1 to 3:1).

R_f = 0.69 (Hex:EA 3:1).

$[\alpha]_D$ -54.58 cm^{-1} (*c* 1, $CHCl_3$). **IR** (film): 3067, 2924, 1752, 1721, 1585, 1494, 1451, 1385, 1244, 1176, 1090, 988, 848, 758, 740, 709 cm^{-1}. **HRMS** (QToF): Calcd for $C_{42}H_{38}O_7SNa$ $[M + Na]^+$ 709.2230; found 709.2250.

^{1}H NMR (600 MHz, $CDCl_3$): δ 8.10 – 8.05 (m, 2H), 7.79 (d, J = 7.5 Hz, 2H), 7.66 – 7.53 (m, 3H), 7.49 – 7.39 (m, 4H), 7.38 – 7.25 (m, 6H), 7.22 (q, J = 2.5 Hz, 3H), 7.13 (d, J = 8.2 Hz, 2H), 5.80 (dd, J = 3.3, 1.7 Hz, 1H), 5.50 (d, J = 1.8 Hz, 1H), 5.06 (t, J = 9.8 Hz, 1H), 4.65 (dd, J = 94.5, 12.1 Hz, 2H), 4.47 (d, J = 7.3 Hz, 2H), 4.42 (dd, J = 9.8, 6.2 Hz, 1H), 4.26 (t, J = 7.2 Hz, 1H), 4.01 (dd, J = 9.7, 3.3 Hz, 1H), 2.33 (s, 3H), 1.32 (d, J = 6.3 Hz, 3H) ppm.

^{13}C NMR (151 MHz, $CDCl_3$): δ 165.8, 155.0, 143.5, 143.3, 141.5, 141.4, 138.3, 137.4, 133.5, 132.5, 130.1, 130.1, 129.7, 129.6, 128.6, 128.5, 128.1, 127.9, 127.3, 125.2, 125.2, 120.2, 86.6, 77.4, 75.0, 71.6, 70.9, 70.1, 67.7, 47.0, 21.3, 17.6 ppm.

^{1}H NMR (400 MHz, $CDCl_3$) of **5.04b**:

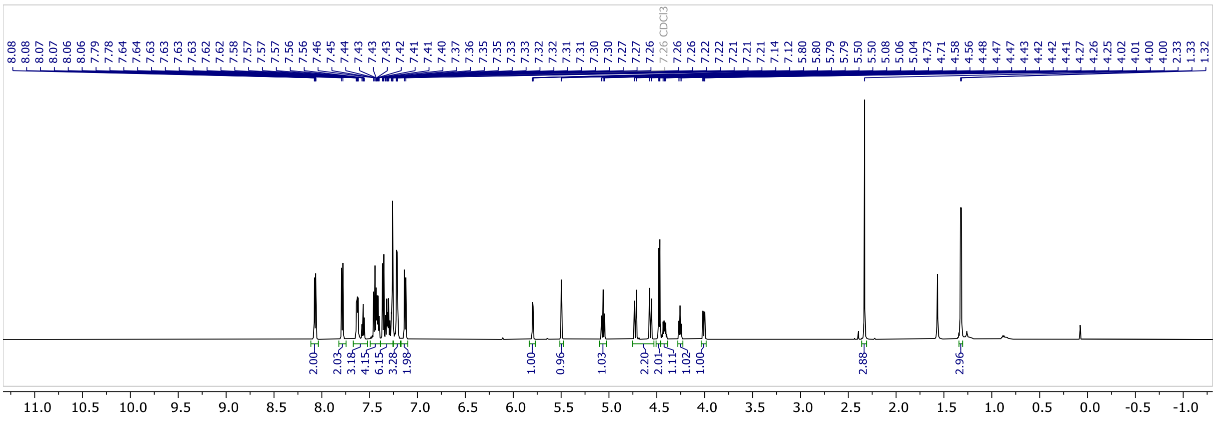

^{13}C NMR (101 MHz, $CDCl_3$) of **4**:

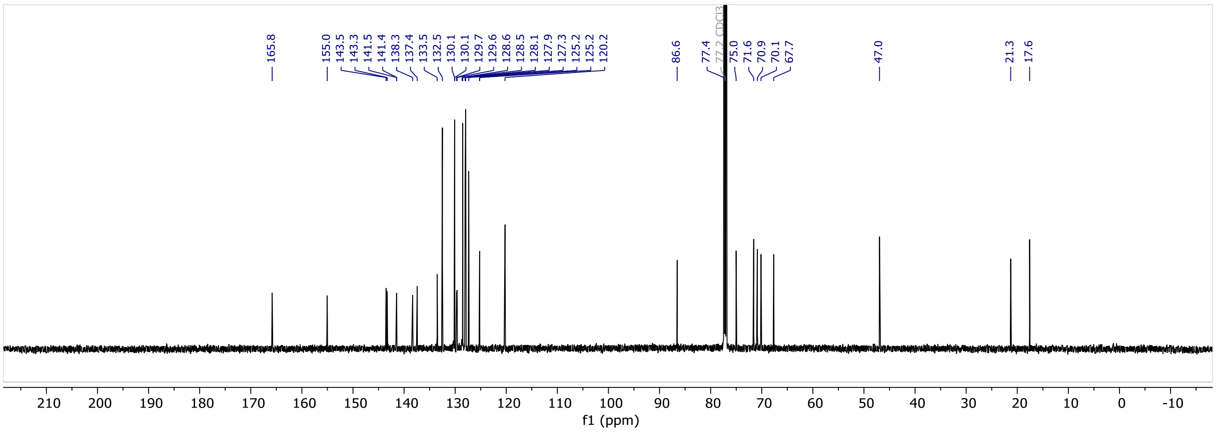

Coupled ^{13}C,^{1}H HSQC of **4**:

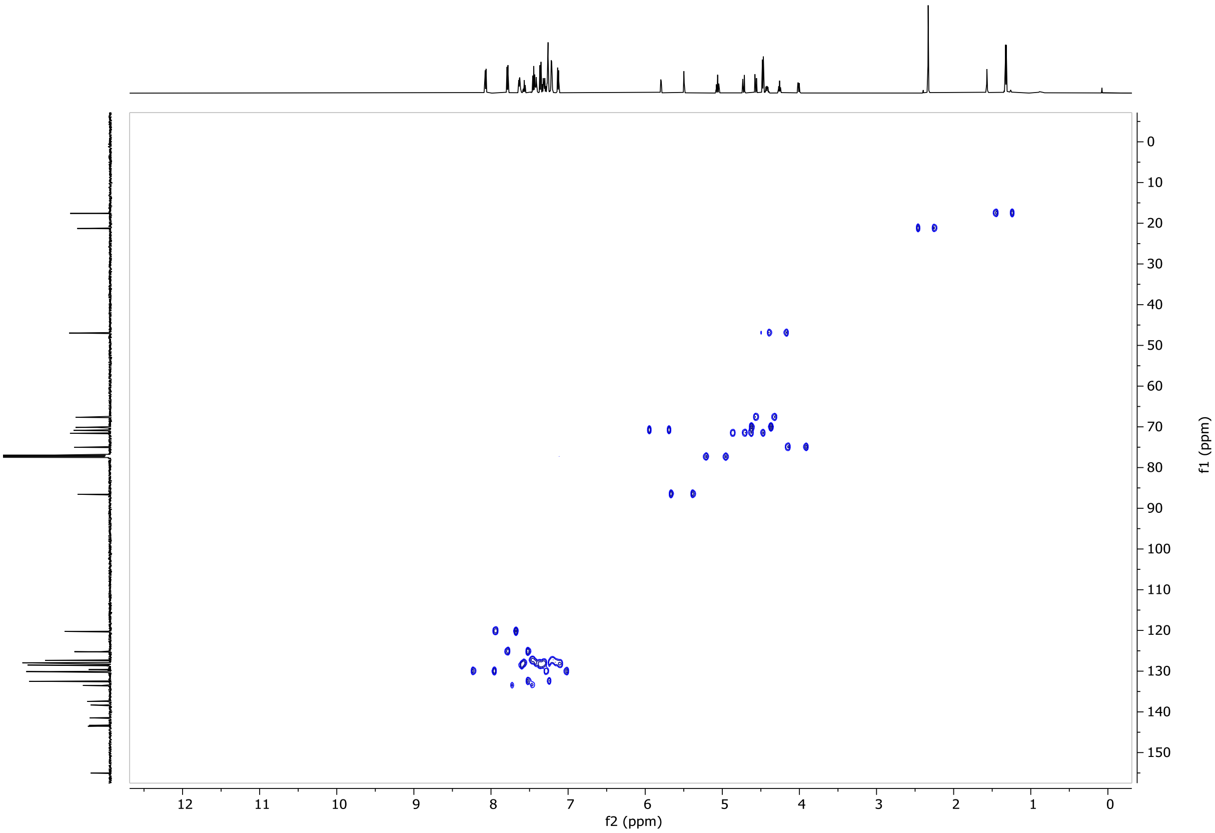

$^{13}C,^{1}H$ HSQC of **4**:

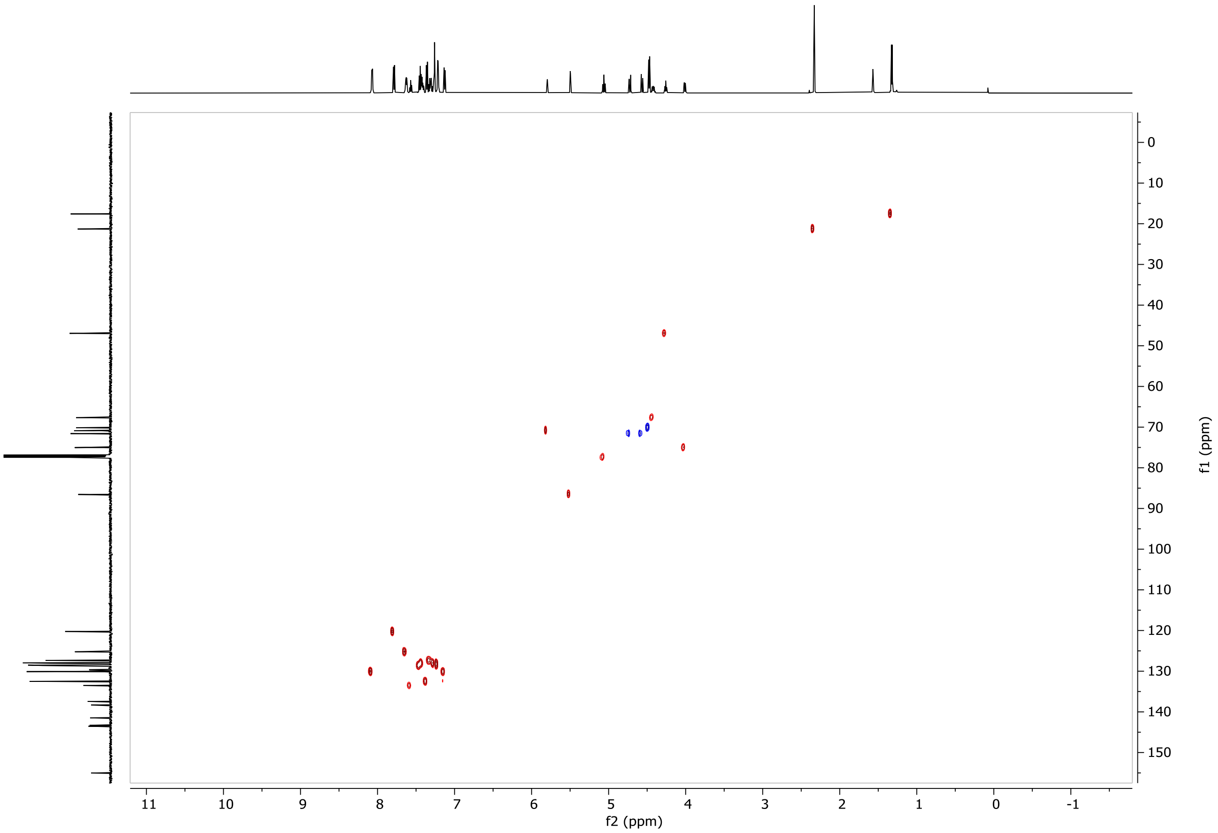

4.2 Galactose

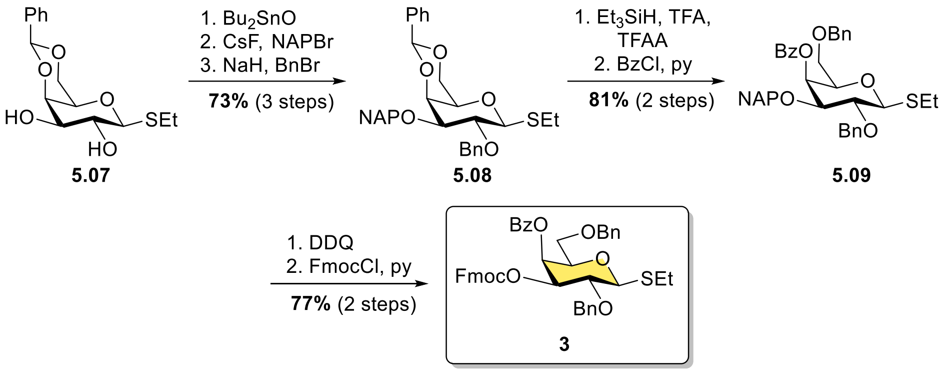

Scheme 2. Synthetic route to α-galactose building block **5.03**.

Ethyl 2-*O*-benzyl-3-*O*-(2-naphthalenylmethyl)-4,6-*O*-[(*S*)-phenylmethylene]-1-thio-β-D-galactopyranoside[4] (5.08)

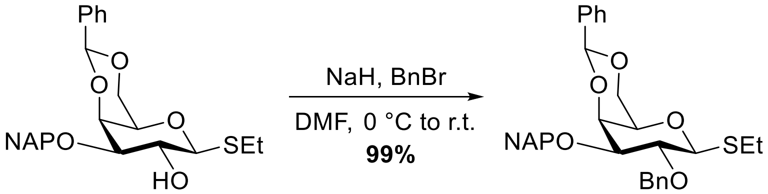

5.08

2-OH-galactose[4] (7.4 g, 16.4 mmol, 1.0 equiv.) was dissolved in anhydrous DMF (75 mL). The stirred solution was cooled to 0 °C and sodium hydride (1.5 g, 24.6 mmol; 60% dispersion in mineral oil, 2.3 equiv.) was added in small portions. After 30 min, benzyl bromide (1.3 mL, 37.7 mmol, 1.5 equiv.) was added dropwise. The reaction mixture was allowed to warm up to room temperature and was stirred overnight. Methanol (10 mL) was added, the reaction mixture was stirred for 10 min and afterwards diluted with ethyl acetate (100 mL). The organic layer was washed with water (2 x 100 mL). The aqueous phase was extracted with ethyl acetate (2 x 100 mL). The combined organic phase was washed with water (100 mL), dried over Na_2SO_4 and concentrated. Product **5.08** (8.8 g, 16.2 mmol, **99%**) was obtained as a colorless solid after purification by column chromatography (SiO_2, Hex/EtOAc = 3:1).

R_f = 0.22 (Hex/EtOAc 3:1).

^{1}H NMR (700 MHz, $CDCl_3$) δ 7.87 – 7.78 (m, 3H), 7.74 – 7.69 (m, 1H), 7.57 – 7.29 (m, 13H), 5.48 (s, 1H), 4.96 – 4.87 (m, 4H), 4.44 (d, *J* = 9.7 Hz, 1H), 4.30 (dd, *J* = 12.3, 1.8 Hz, 1H), 4.17 (d, *J* = 3.7 Hz, 1H), 3.96 – 3.90 (m, 2H), 3.65 (dd, *J* = 9.2, 3.5 Hz, 1H), 3.35 – 3.32 (m, 1H), 2.90 – 2.73 (m, 2H), 1.34 (t, *J* = 7.5 Hz, 3H) ppm.

^{13}C NMR (176 MHz, $CDCl_3$) δ 138.6, 138.1, 135.9, 133.4, 133.2, 129.2, 128.5, 128.5, 128.4, 128.3, 128.0, 127.9, 127.8, 126.7, 126.7, 126.3, 126.1, 126.0, 101.7, 84.6, 81.1, 77.1, 75.9, 74.2, 72.1, 69.9, 69.5, 24.0, 15.2 ppm.

HRMS (QToF): Calcd for $C_{33}H_{34}O_5SNa$ $[M + Na]^+$ 565.2019; found 565.2018.

^1H NMR (400 MHz, $CDCl_3$) of **5.08**:

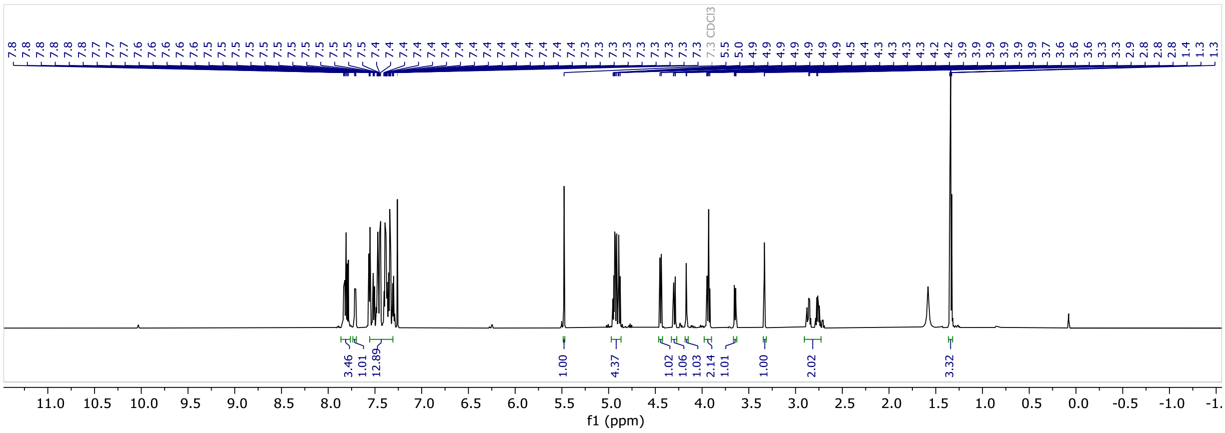

^{13}C NMR (101 MHz, $CDCl_3$) of **5.08**:

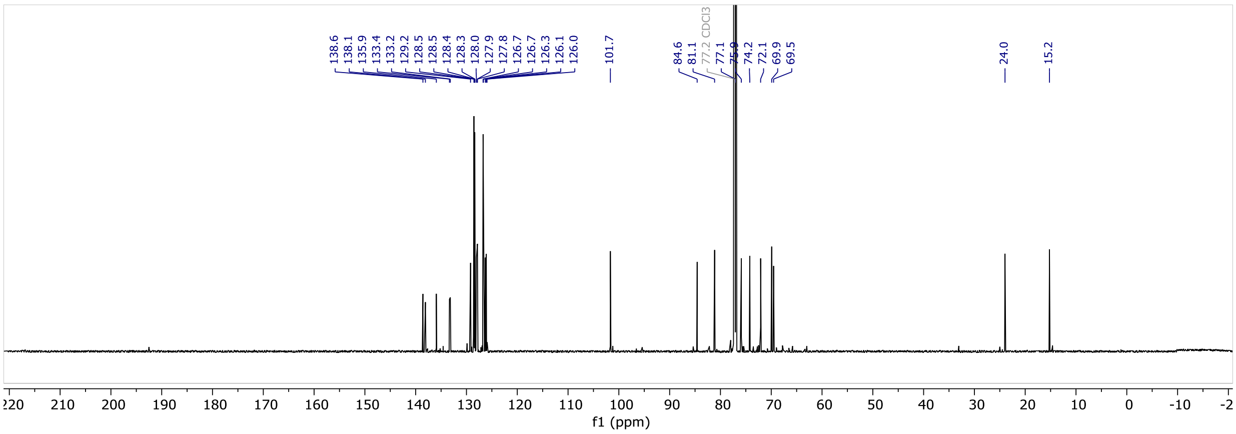

^{13}C,^{1}H HSQC of **5.08**:

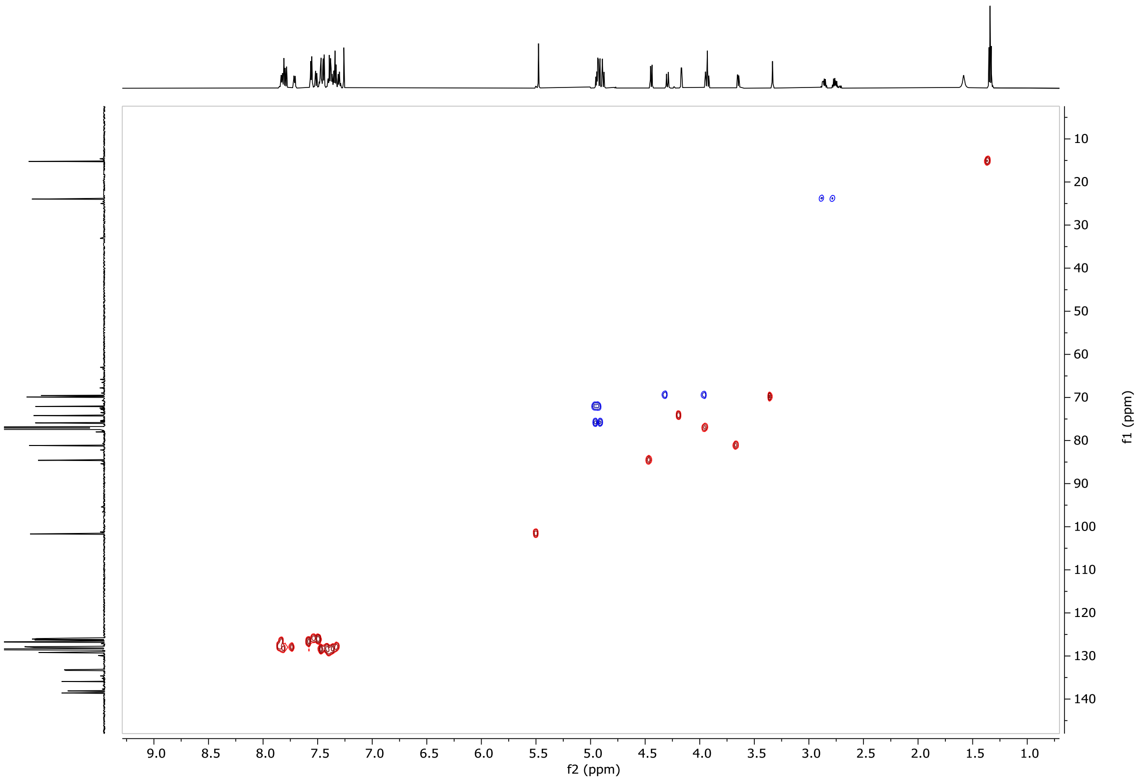

^{1}H,^{1}H COSY of **5.08**:

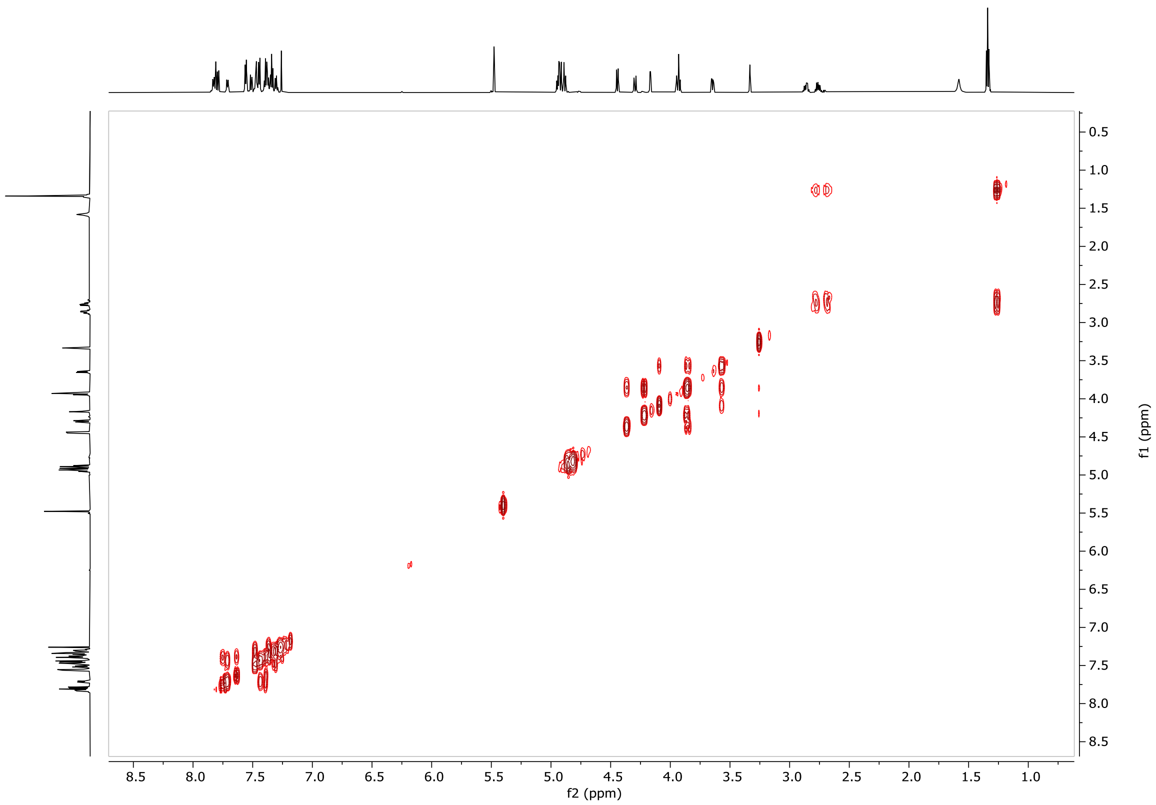

Ethyl 4-*O*-benzoyl-2,6-bis-*O*-benzyl-3-*O*-(2-naphthalenylmethyl)-1-thio-β-D-galactopyranoside (5.09)

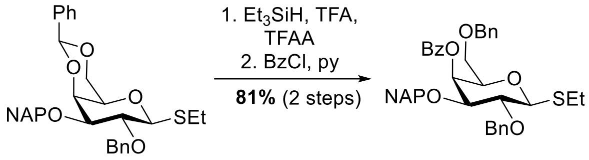

5.08 **5.09**

Compound **5.08** (2.0 g, 3.7 mmol, 1.0 equiv.) was co-evaporated with anhydrous toluene (2 x 3 mL), and dissolved in anhydrous CH_2Cl_2 (20 mL). Triethylsilane (3.5 mL, 22.1 mmol, 6.0 equiv.) and trifluoroacetic anhydride (0.52 mL, 3.7 mmol, 1.0 equiv.) were added and the solution was cooled to 0 °C. Trifluoroacetic acid (1.7 mL, 22.1 mmol, 6.0 equiv.) was added dropwise. The mixture was allowed to warm up to room temperature and was stirred for 5 h. The solution was diluted with CH_2Cl_2 and quenched with saturated aqueous $NaHCO_3$ (40 mL). The aqueous phase was extracted with CH_2Cl_2 (2 x 60 mL) and the combined organic phase was washed with water (60 mL), dried over Na_2SO_4, filtered and concentrated. **4-OH-galactose** (1.8 g, 3.3 mmol, 90%) was obtained as a colorless syrup after purification by column chromatography (SiO_2, Hex/EtOAc = 3:1 to 1:1). **R_f** = 0.39 (Hex/EtOAc 1:1). **4-OH-galactose** (1.4 g, 2.6 mmol, 1.0 equiv.) was dissolved in pyridine (20 mL) and the solution was cooled to 0 °C. Benzoyl chloride (0.89 mL, 7.7 mmol, 3.0 equiv.) was added under an argon atmosphere. The reaction mixture was allowed to warm up to room temperature and was stirred for 16 h. The reaction was quenched by addition of water and the aqueous layer was extracted with ethyl acetate. The combined organic phase was washed with 1.0 M HCl and brine, dried over Na_2SO_4, filtered and concentrated. The title compound **5.09** (1.2 g, 1.8 mmol, 72%) was obtained as a colorless oil after purification by column chromatography (Hex/EtOAc = 9:1 to 3:1).

R_f = 0.31 (Hex:EA 3:1).

HRMS (QToF): Calcd for $C_{40}H_{40}O_6SNa$ $[M + Na]^+$ 671.2438; found 671.2488.

^{1}H NMR (400 MHz, $CDCl_3$): δ 8.16 – 7.98 (m, 3H), 7.81 – 7.68 (m, 3H), 7.65 – 7.56 (m, 2H), 7.52 – 7.13 (m, 14H), 5.94 (dd, *J* = 3.3, 1.0 Hz, 1H), 5.02 (d, *J* = 11.6 Hz, 1H), 4.89 – 4.66 (m, 3H), 4.57 – 4.36 (m, 3H), 3.84 (d, *J* = 1.2 Hz, 1H), 3.77 (d, *J* = 3.3 Hz, 1H), 3.75 – 3.61 (m, 2H), 3.58 (d, *J* = 7.1 Hz, 1H), 2.78 – 2.66 (m, 2H), 1.34 (t, *J* = 7.5 Hz, 3H) ppm.

^{13}C NMR (101 MHz, $CDCl_3$): δ 165.9, 138.2, 137.7, 135.4, 133.8, 133.3, 133.1, 130.3, 130.2, 130.0, 128.6, 128.5, 128.5, 128.2, 128.1, 127.9, 127.0, 126.3, 126.0, 125.9, 99.7, 85.6, 81.1, 77.9, 77.4, 76.2, 76.0, 73.9, 71.9, 68.4, 67.6, 25.1, 15.2 ppm.

^{1}H NMR (400 MHz, $CDCl_3$) of **5.09**:

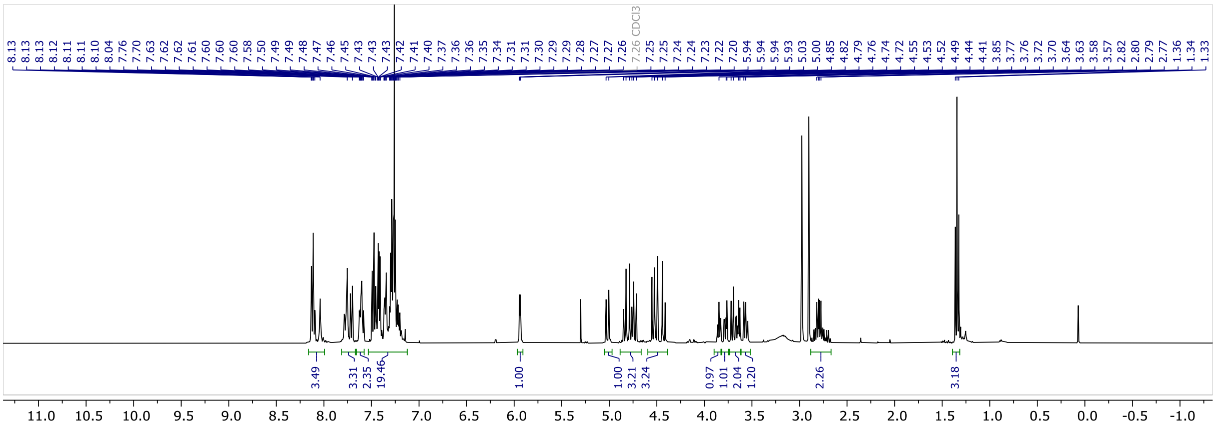

^{13}C NMR (101 MHz, $CDCl_3$) of **5.09**:

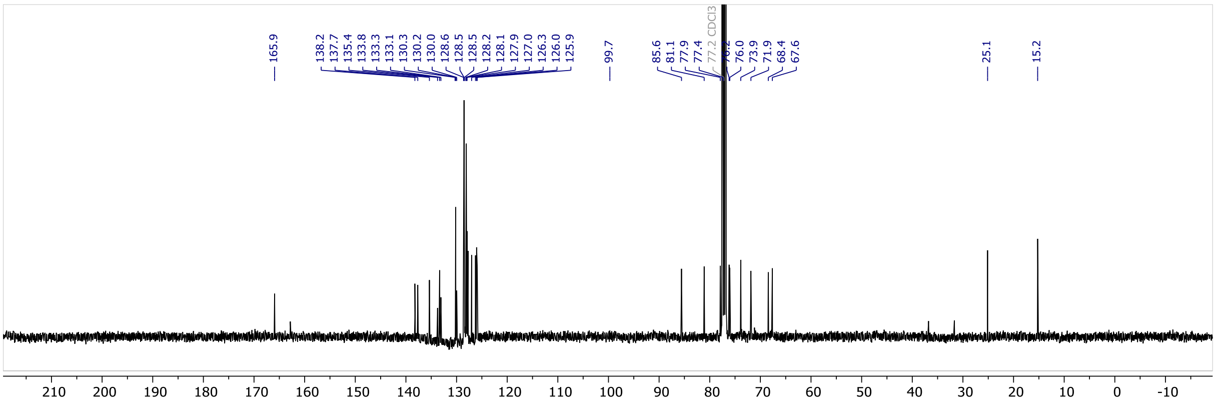

^{13}C,^{1}H HSQC of **5.09**:

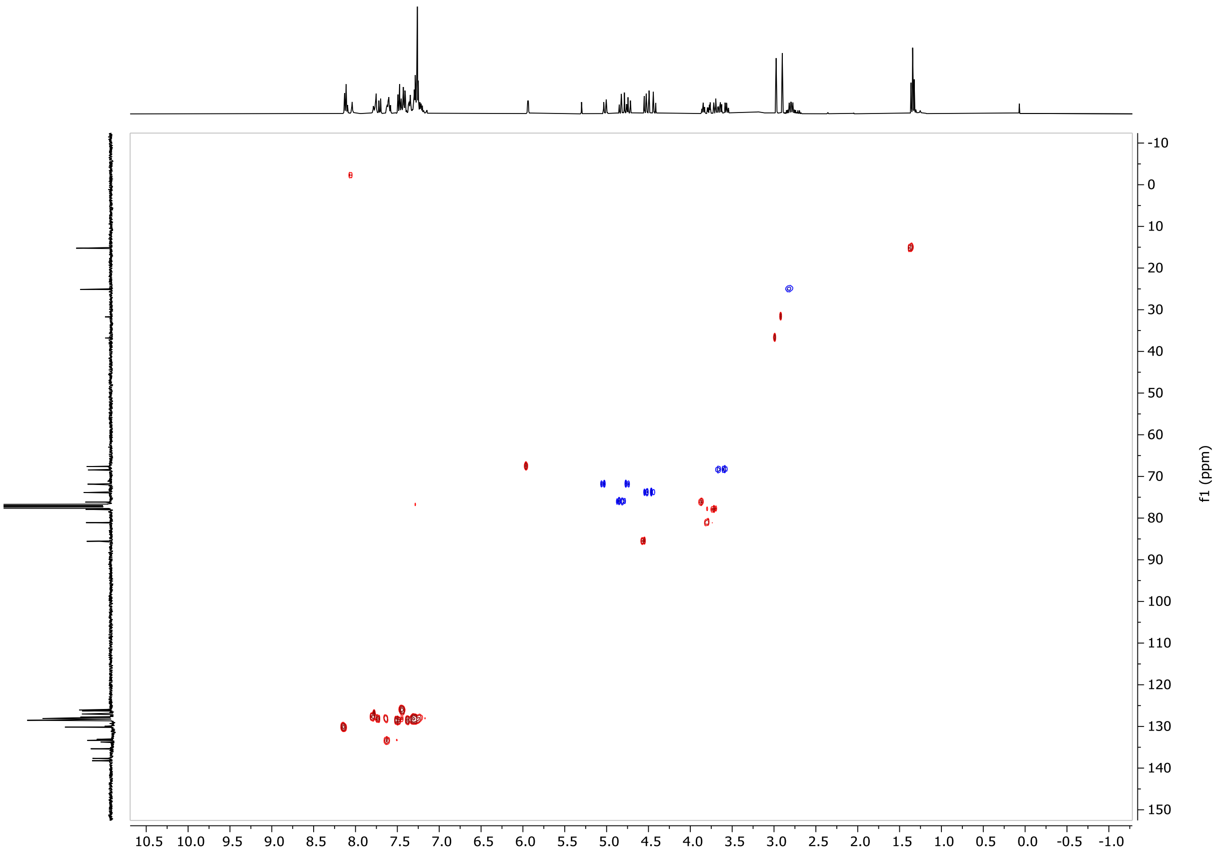

^{1}H,^{1}H COSY of **5.09**:

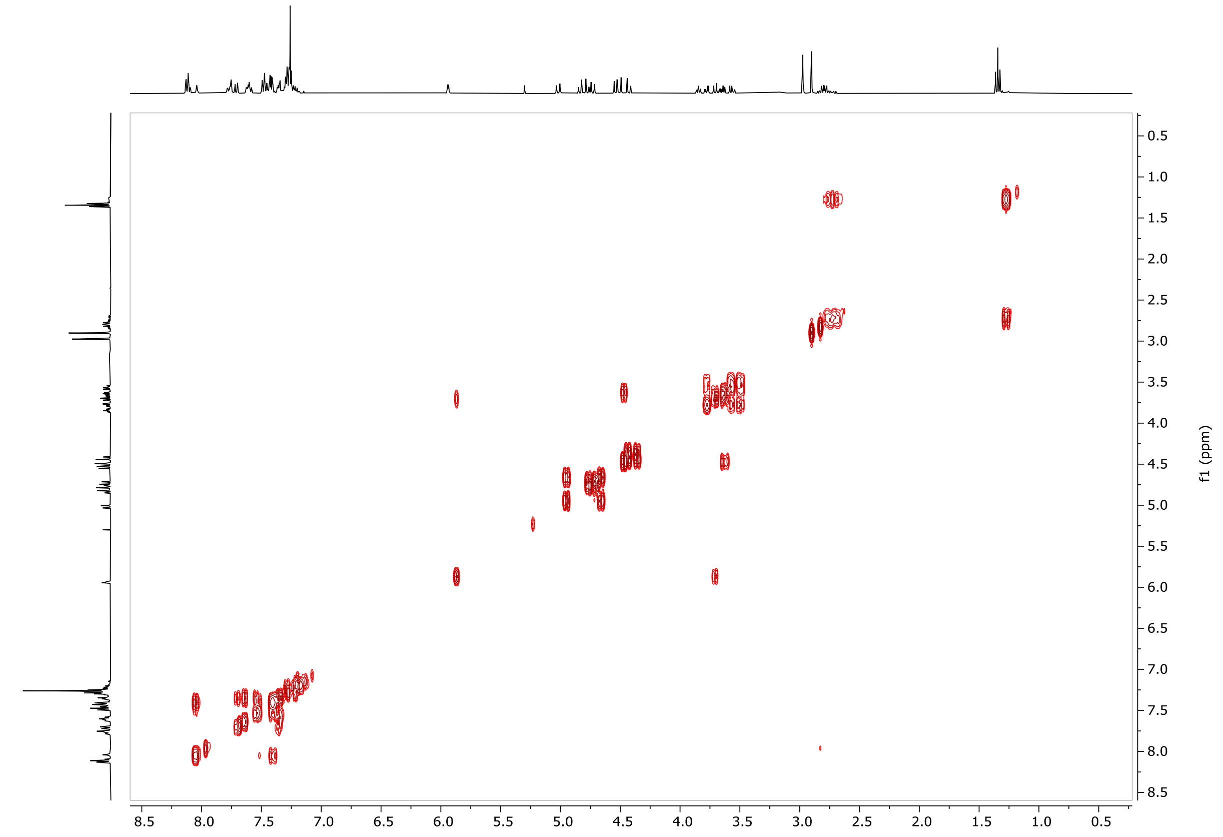

Ethyl 4-*O*-benzoyl-2,6-bis-*O*-benzyl-3-*O*-fluorenylmethoxycarbonyl-1-thio-β-D-galactopyranoside (3)

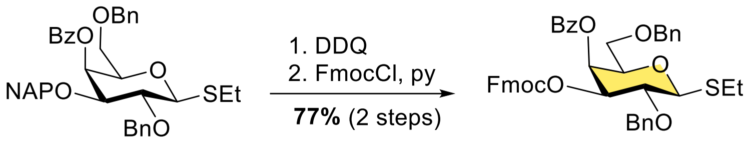

5.09 **3**

To a well stirred emulsion of **5.09** (1.8 g, 2.8 mmol, 1.0 equiv.) in CH_2Cl_2/water (7:1, 24 mL), was added DDQ (692 mg, 3.1 mmol, 1.1 equiv.) and the suspension was stirred at room temperature for 1.5 h protected from light. The mixture was diluted with CH_2Cl_2, washed with aqueous 10% $Na_2S_2O_3$ and saturated aqueous $NaHCO_3$ solution. The organic layer was dried over Na_2SO_4, filtered, concentrated and the residue was purified by column chromatography (Hex/EA 9:1 to 3:1) to obtain the title compound (1.1 g, 2.2 mmol, 78%) as a colorless solid. R_f = 0.35 (Hex:EA 3:1). To a solution of **4-OH-sugar** (1.1 g, 2.2 mmol, 1.0 equiv.) and anhydrous pyridine (0.87 mL, 10.8 mmol, 5.0 equiv.) in anhydrous CH_2Cl_2 (15 mL) at 0 °C was added FmocCl (838 mg, 3.2 mmol, 1.5 equiv.) followed by 4-DMAP (13 mg, 0.11 mmol, 0.05 equiv.) and the reaction mixture was stirred for two hours at 0 °C. Aqueous citric acid solution (10 mL) was added and the mixture was allowed to warm up to room temperature. The aqueous phase was extracted with CH_2Cl_2 and the combined organic phase was dried over Na_2SO_4, filtered and concentrated. The title compound **3** (1.2, 1.6 mmol, 76%) was obtained as a white solid after purification by column chromatography (Hex/EtOAc = 19:1 to 3:1).

R_f = 0.5 (Hex:EA 3:1).

$[\alpha]_D$ 14.29 cm^{-1} (*c* 1, $CHCl_3$). **IR** (film): 3066, 2927, 2869, 1750, 1726, 1603, 1497, 1451, 1388, 1274, 1246, 1096, 1070, 1026, 984, 907, 758, 737, 697 cm^{-1}. **HRMS** (QToF): Calcd for $C_{44}H_{42}O_8SNa$ $[M + Na]^+$ 753.2493; found 753.2518.

^{1}H NMR (400 MHz, $CDCl_3$): δ 8.10 – 8.03 (m, 2H), 7.76 (ddt, *J* = 7.7, 1.9, 0.9 Hz, 2H), 7.63 (d, *J* = 7.4 Hz, 1H), 7.60 – 7.52 (m, 2H), 7.49 (t, *J* = 7.8 Hz, 2H), 7.44 – 7.31 (m, 4H), 7.31 – 7.12 (m, 10H), 5.90 (dd, *J* = 3.4, 1.1 Hz, 1H), 5.00 (dd, *J* = 9.6, 3.4 Hz, 1H), 4.88 (d, *J* = 10.5 Hz, 1H), 4.71 (d, *J* = 10.5 Hz, 1H), 4.66 – 4.56 (m, 2H), 4.39 (d, *J* = 12.0 Hz, 1H), 4.31 (t, *J* = 7.4 Hz, 1H), 4.19 (dd, *J* = 10.3, 8.3 Hz, 1H), 3.98 (td, *J* = 6.4, 1.1 Hz, 1H), 3.81 (t, *J* = 9.7 Hz, 1H), 3.59 (ddd, *J* = 33.9, 9.6, 6.4 Hz, 2H), 2.93 – 2.74 (m, 2H), 1.37 (t, *J* = 7.4 Hz, 3H) ppm.

^{13}C NMR (101 MHz, $CDCl_3$): δ 165.77, 154.36, 143.97, 143.20, 141.42, 141.29, 137.75, 137.57, 133.52, 130.26, 129.56, 128.62, 128.47, 128.28, 127.99, 127.97, 127.94, 127.87,

127.85, 127.31, 127.23, 125.67, 125.34, 120.10, 85.56, 78.91, 76.29, 75.97, 75.90, 73.69, 70.48, 68.54, 68.03, 46.78, 25.37, 15.21 ppm.

^{1}H NMR (600 MHz, $CDCl_3$) of **3**:

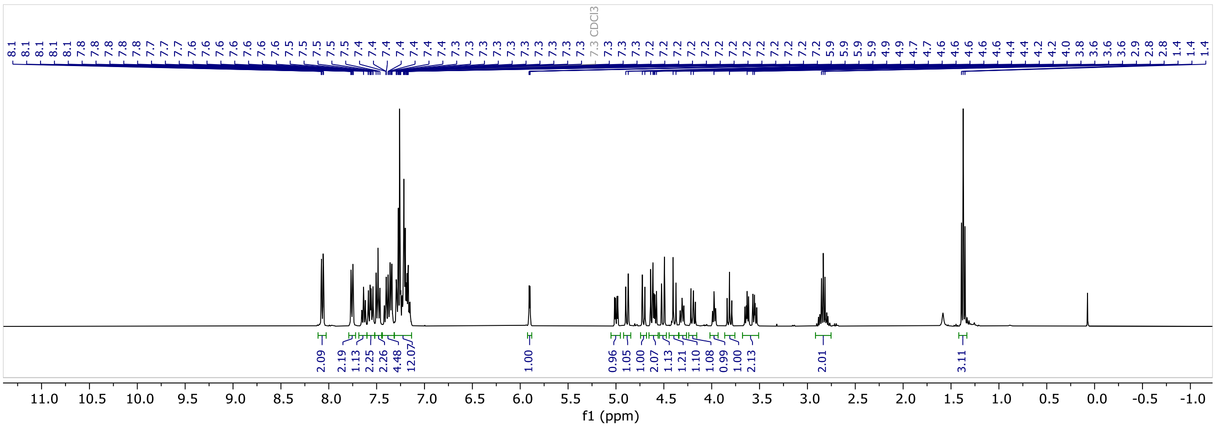

^{13}C NMR (151 MHz, $CDCl_3$) of **3**:

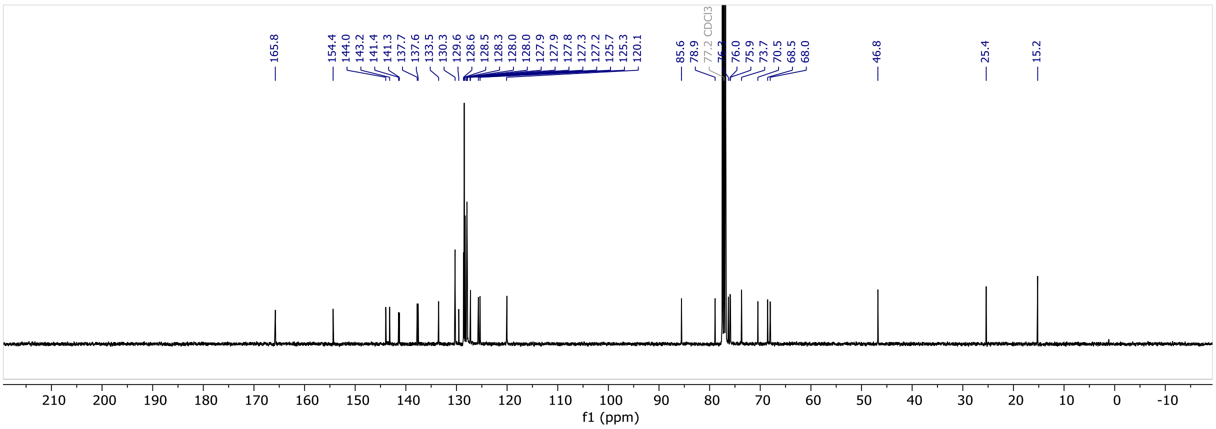

$^{13}C,^{1}H$ HSQC of **5.03**:

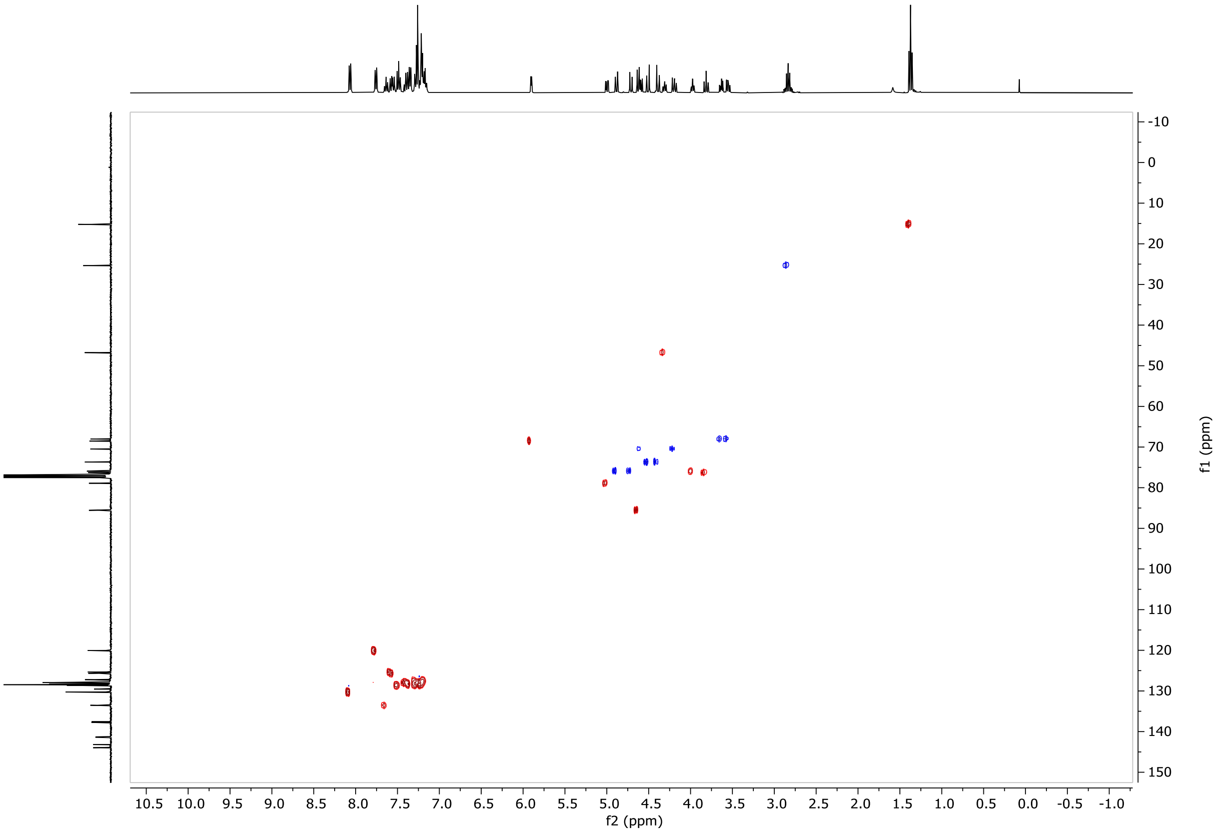

$^{1}H,^{1}H$ COSY of **5.03**:

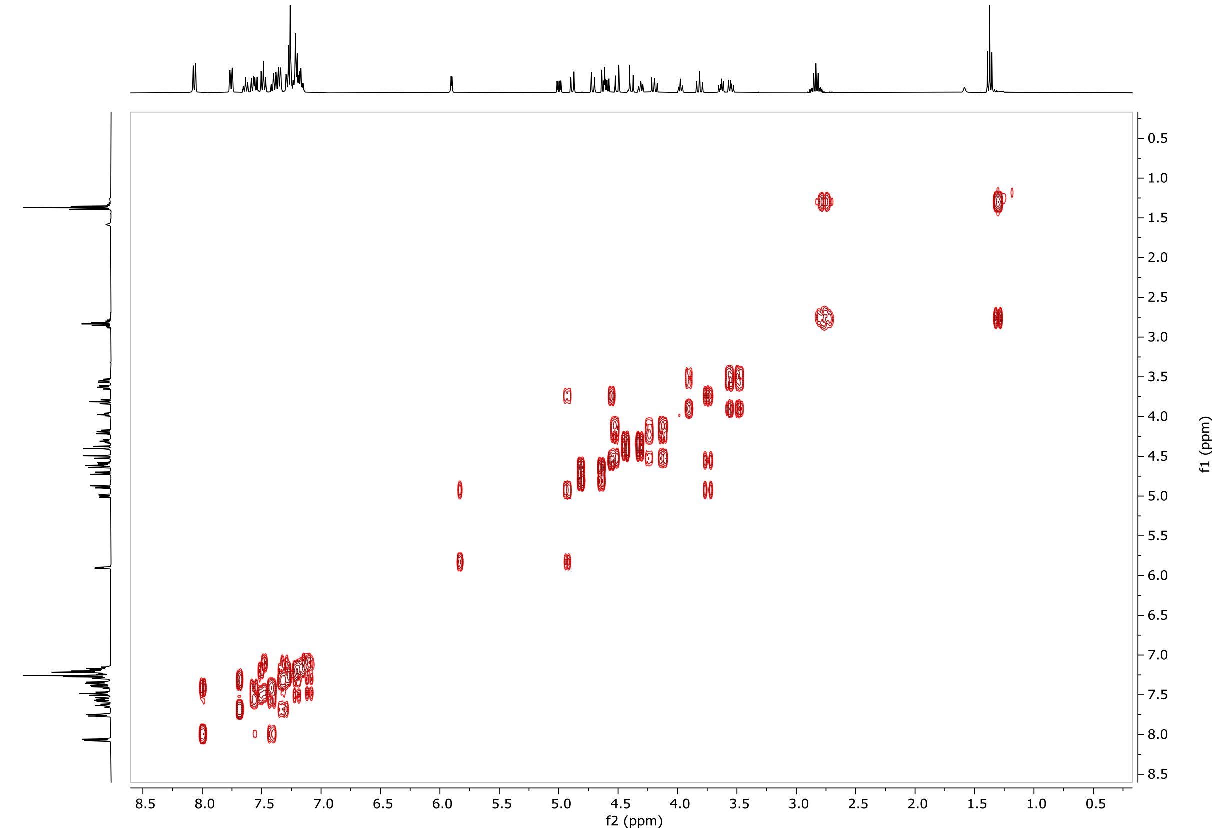

4.3 Glucose

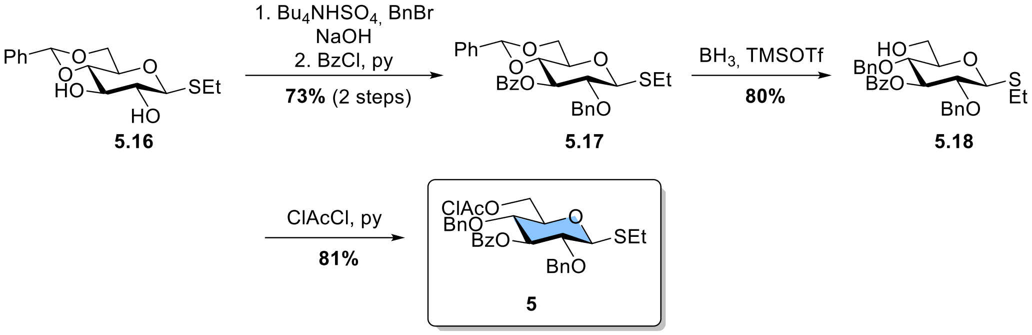

Scheme 3. Synthetic route to α-glucose building block **5.05**.

Ethyl 2-*O*-benzyl-4,6-*O*-[(*S*)-phenylmethylene]-1-thio-β-D-glucopyranoside[5] (5.16a)

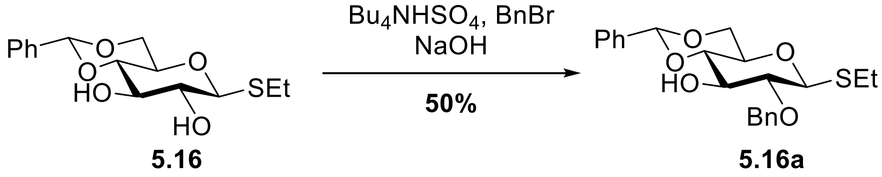

Tetrabutylammonium hydrogen sulfate (2.2 g, 6.4 mmol, 0.5 equiv.), benzyl bromide (1.68 mL, 14.1 mmol, 1.1 equiv., filtered using a short aluminium oxide column before use) and 5% aqueous NaOH (33 mL) was added to a stirred solution of ethyl 4,6-*O*-benzylidene-1-thio-β- D-glucopyranoside (4.0 g, 12.8 mmol, 1.0 equiv.) in CH_2Cl_2 (300 mL). The mixture was heated to 45 °C and was stirred for 8 h. The mixture was allowed to cool down to room temperature and the organic phase was separated from the aqueous phase. The aqueous phase was extracted with CH_2Cl_2 (2 x 100 mL) and the combined organic phase was washed with brine (100 mL), dried over Na_2SO_4, filtered and concentrated. 3-OH-galactose **5.16a** (2.6 g, 6.5 mmol, 50%) was obtained as a colorless solid after purification by column chromatography (SiO_2, Toluene/EtOAc = 95:5 to 9:1). All spectra are in accordance with literature spectra.

R_f = 0.42 (Toluene/EtOAc 9:1).

$[\alpha]_D$ 24.1 cm^{-1} (*c* 1, $CHCl_3$). **IR** (film): 3066, 2962, 2884, 1761, 1722, 1602, 1453, 1406, 1335, 1266, 1248, 1199, 1135, 1061, 1027, 998, 916, 805, 753, 700 cm^{-1}. **HRMS** (QToF): Calcd for $C_{31}H_{33}ClO_7SNa$ $[M + Na]^+$ 607.1528; found 607.1539.

^1H NMR (400 MHz, $CDCl_3$): δ 7.54 – 7.28 (m, 10H), 5.52 (s, 1H), 4.87 (dd, *J* = 68.7, 10.8 Hz, 2H), 4.57 (d, *J* = 9.8 Hz, 1H), 4.35 (dd, *J* = 10.4, 4.9 Hz, 1H), 3.89 (t, *J* = 8.8 Hz, 1H), 3.76 (t, *J* = 10.2 Hz, 1H), 3.56 (d, *J* = 9.3 Hz, 1H), 3.45 (d, *J* = 5.0 Hz, 1H), 3.38 (dd, *J* = 9.8, 8.3 Hz, 1H), 2.87 – 2.68 (m, 2H), 1.34 (t, *J* = 7.5 Hz, 3H) ppm.

^{1}H NMR (400 MHz, $CDCl_3$) of **5.16a**:

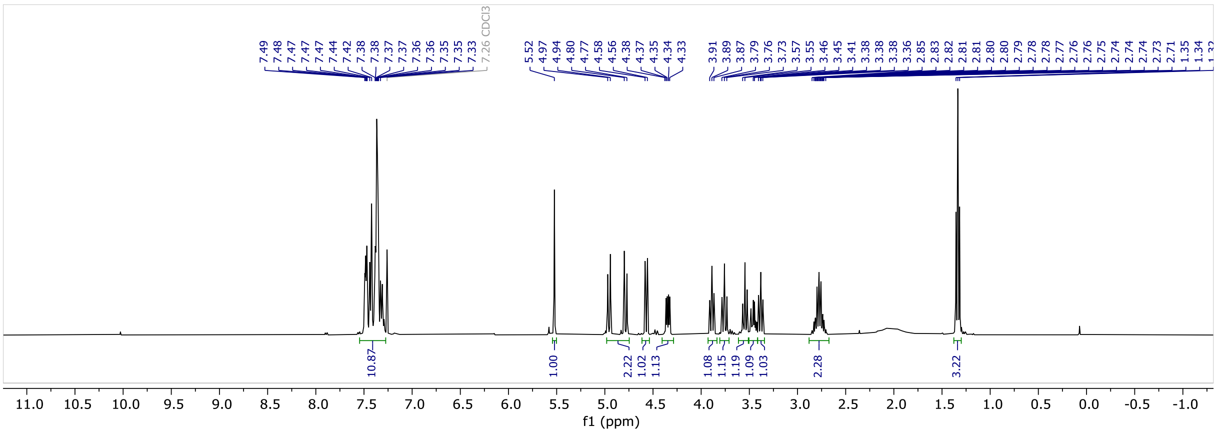

Ethyl 3-*O*-benzoyl-2,4-bis-*O*-benzyl-1-thio-β-D-glucopyranoside (5.18)

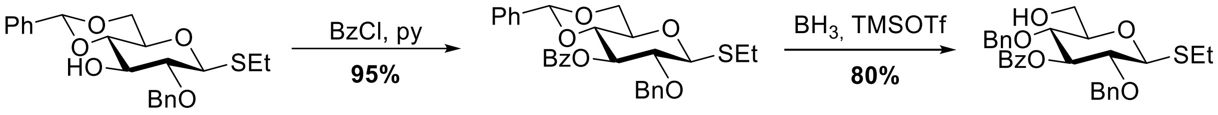

5.16 **5.17** **5.18**

3-OH-galactose **5.16a** (2.6 g, 6.5 mmol, 1.0 equiv.) was dissolved in pyridine (30 mL) and the solution was cooled to 0 °C. Benzoyl chloride (2.26 mL, 19.4 mmol, 3.0 equiv.) was added under an argon atmosphere. The reaction mixture was allowed to warm up to room temperature and was stirred for 16 h. The reaction was quenched by addition of water and the aqueous layer was extracted with ethyl acetate (3 x 100 mL). The combined organic phase was washed with 1.0 M HCl and brine, dried over Na_2SO_4, filtered and concentrated. Compound **5.17** (3.1 g, 6.1 mmol, 95%) was obtained as a colorless oil after purification by column chromatography (Hex/EtOAc = 9:1 to 3:1). **R*f*** = 0.29 (Hex/EtOAc 9:1). Compound **5.17** (1.0 g, 2.0 mmol, 1.0 equiv.) was co-evaporated by anhydrous toluene and dried for 2 h at high vacuum. Then, it was dissolved in anhydrous CH_2Cl_2 (25 mL) and the mixture was stirred over activated molecular sieves (3 Å-AW) for 30 minutes at room temperature. The mixture was cooled to 0 °C and BH_3 (1 M solution in THF, 9.9 mL, 9.9 mmol, 5.0 equiv.) and TMSOTf (53 µL, 0.29 mmol, 0.15 equiv.) were added dropwise. The mixture was allowed to warm up to room temperature and stirred under argon atmosphere for 4 h. Et_3N (1 mL) was added followed by MeOH until the evolution of H_2 ceased. The mixture was concentrated and co-evaporated with MeOH (3 x 30 mL). 6-OH-sugar **5.18** (805 mg, 1.6 mmol, 80%) was obtained as a white solid after purification by column chromatography (Hex:AcOEt 3:1).

R*f* = 0.18 (Hex: AcOEt 3:1).

HRMS (QToF): Calcd for $C_{29}H_{32}O_6SNa$ $[M + Na]^+$ 531.1812; found 531.1830.

^{1}H NMR (600 MHz, CDCl$_3$): δ 8.02 – 7.97 (m, 2H), 7.61 – 7.55 (m, 1H), 7.48 – 7.41 (m, 2H), 7.20 – 7.07 (m, 10H), 5.58 (t, *J* = 9.2 Hz, 1H), 4.79 (d, *J* = 10.7 Hz, 1H), 4.62 (d, *J* = 9.7 Hz, 1H), 4.55 (s, 2H), 4.54 (d, *J* = 10.7 Hz, 1H), 3.92 (dd, *J* = 12.2, 2.6 Hz, 1H), 3.80 – 3.72 (m, 2H), 3.54 – 3.47 (m, 2H), 2.84 – 2.73 (m, 2H), 1.33 (t, *J* = 7.5 Hz, 3H) ppm.

^{13}C NMR (151 MHz, CDCl$_3$): δ 165.6, 137.4, 133.3, 130.1, 129.9, 128.6, 128.5, 128.4, 128.1, 127.9, 85.4, 79.5, 79.2, 78.0, 75.7, 75.1, 74.8, 62.0, 25.6, 15.3 ppm.

^{1}H NMR (600 MHz, CDCl$_3$) of **5.18**:

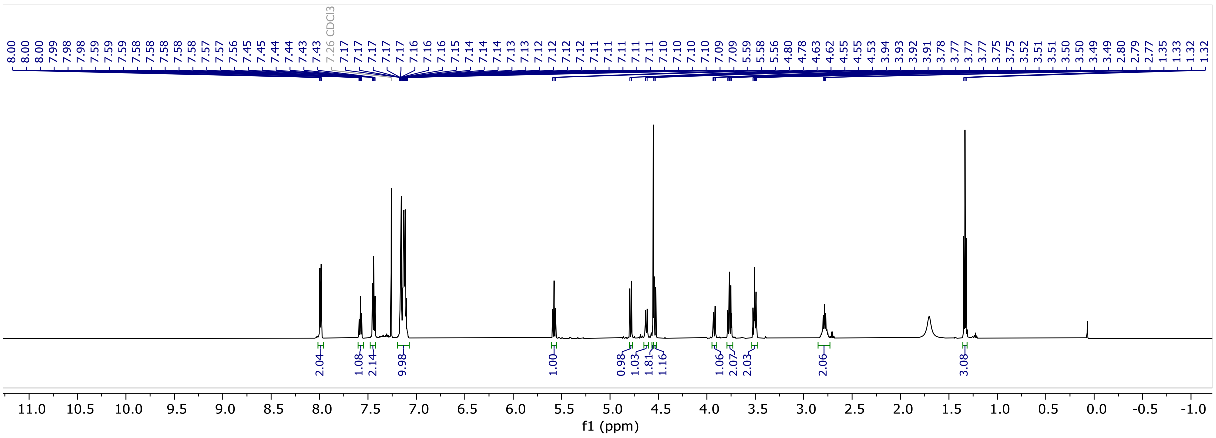

^{13}C NMR (151 MHz, CDCl$_3$) of **5.18**:

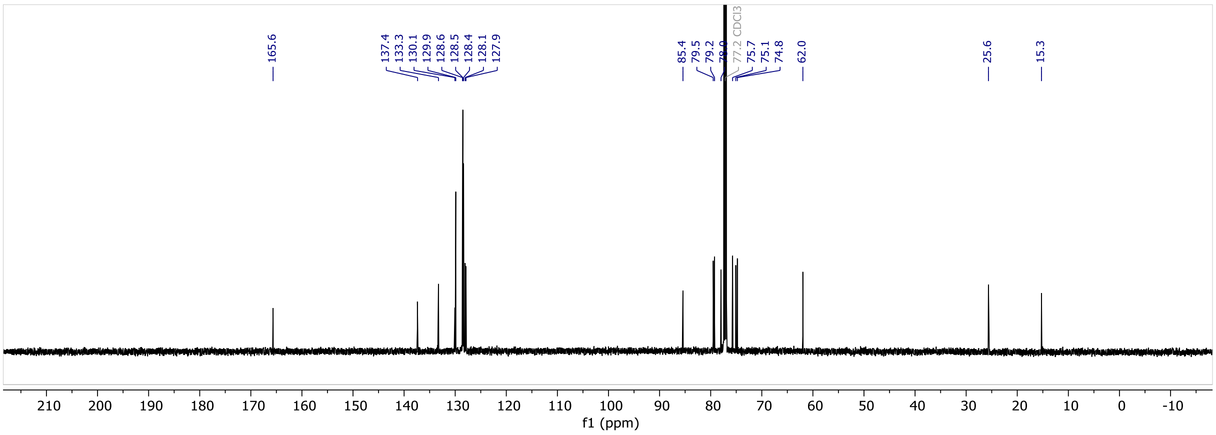

$^{13}C,^{1}H$ HSQC of **5.18**:

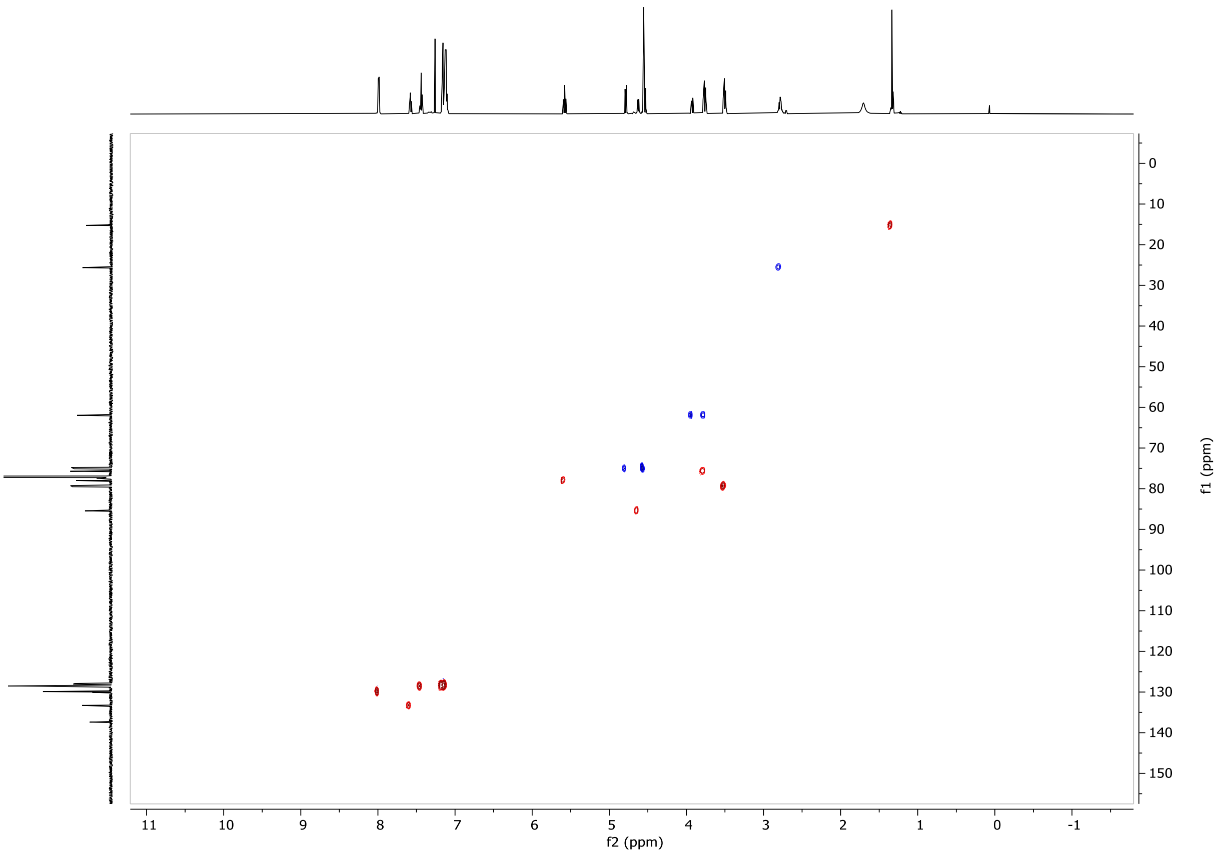

$^{1}H,^{1}H$ COSY of **5.18**:

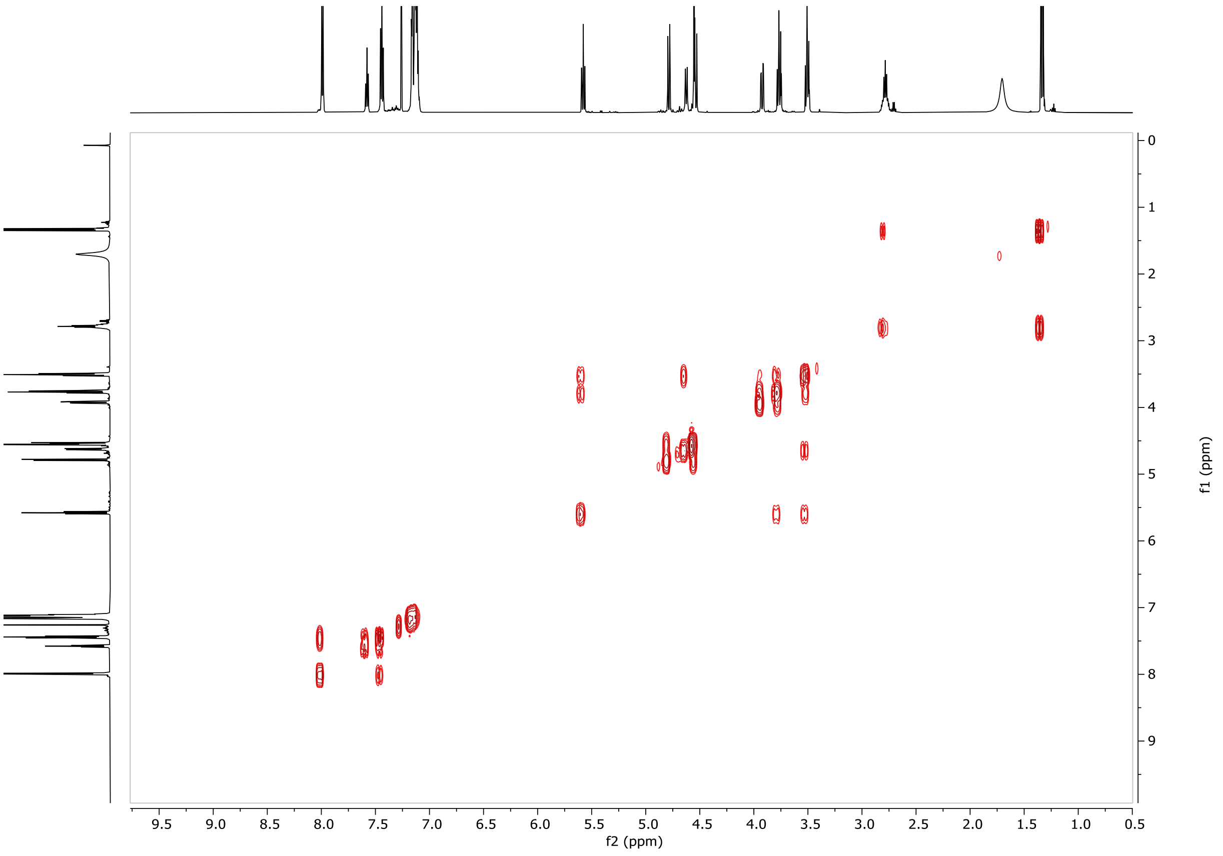

Ethyl 3-*O*-benzoyl-2,4-bis-*O*-benzyl-6-*O*-(2-chloroacetyl)-1-thio-β-D-glucopyranoside (5)

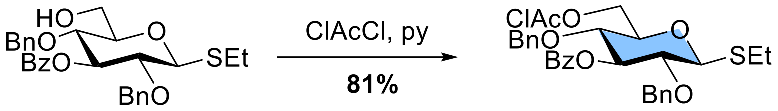

5.18 **5**

To a solution of 6-OH-sugar **5.18** (1.5 g, 2.9 mmol, 1.0 equiv.) and anhydrous pyridine (1.19 mL, 14.8 mmol, 5.0 equiv.) in anhydrous CH_2Cl_2 (20 mL) at -20 °C was added chloroacetyl chloride (0.47 mL, 5.9 mmol, 2.0 equiv.). The reaction was stirred for 10 min, aqueous citric acid solution (2 mL) was added and the mixture was allowed to warm up to room temperature. Water was added and the aqueous phase was extracted with CH_2Cl_2 (3 x 50 mL). The combined organic phase was dried over Na_2SO_4, filtered and concentrated. The title compound **5** (1.4 g, 2.4 mmol, 81%) was obtained as a light-yellow solid after purification by column chromatography (Hex:AcOEt 9:1 to 3:1).

R_f = 0.44 (Hex: AcOEt 3:1).

$[\alpha]_D$ 24.1 cm^{-1} (*c* 1, $CHCl_3$).

IR (film): 3066, 2962, 2884, 1761, 1722, 1602, 1453, 1406, 1335, 1266, 1248, 1199, 1135, 1061, 1027, 998, 916, 805, 753, 700 cm^{-1}.

HRMS (QToF): Calcd for $C_{31}H_{33}ClO_7SNa$ $[M + Na]^+$ 607.1528; found 607.1539.

^{1}H NMR (600 MHz, $CDCl_3$): δ 8.05 – 8.00 (m, 2H), 7.62 – 7.56 (m, 1H), 7.46 (t, *J* = 7.8 Hz, 2H), 7.20 (dd, *J* = 4.9, 1.9 Hz, 3H), 7.12 (dq, *J* = 5.5, 3.0 Hz, 7H), 5.59 (t, *J* = 8.8 Hz, 1H), 4.79 (d, *J* = 10.7 Hz, 1H), 4.64 – 4.51 (m, 3H), 4.48 – 4.43 (m, 2H), 4.29 (dd, *J* = 11.9, 4.6 Hz, 1H), 4.03 (q, *J* = 14.8 Hz, 2H), 3.68 (t, *J* = 6.4 Hz, 2H), 3.53 (t, *J* = 9.4 Hz, 1H), 2.77 (dtt, *J* = 20.1, 12.6, 7.4 Hz, 2H), 1.33 (t, *J* = 7.4 Hz, 3H) ppm.

^{13}C NMR (151 MHz, $CDCl_3$): δ 167.1, 165.6, 137.3, 137.0, 133.4, 129.9, 128.7, 128.6, 128.5, 128.5, 128.4, 128.3, 127.9, 85.4, 79.3, 78.1, 76.5, 75.7, 75.1, 74.7, 64.8, 40.9, 25.6, 15.2 ppm.

^{1}H NMR (600 MHz, $CDCl_3$) of **5**:

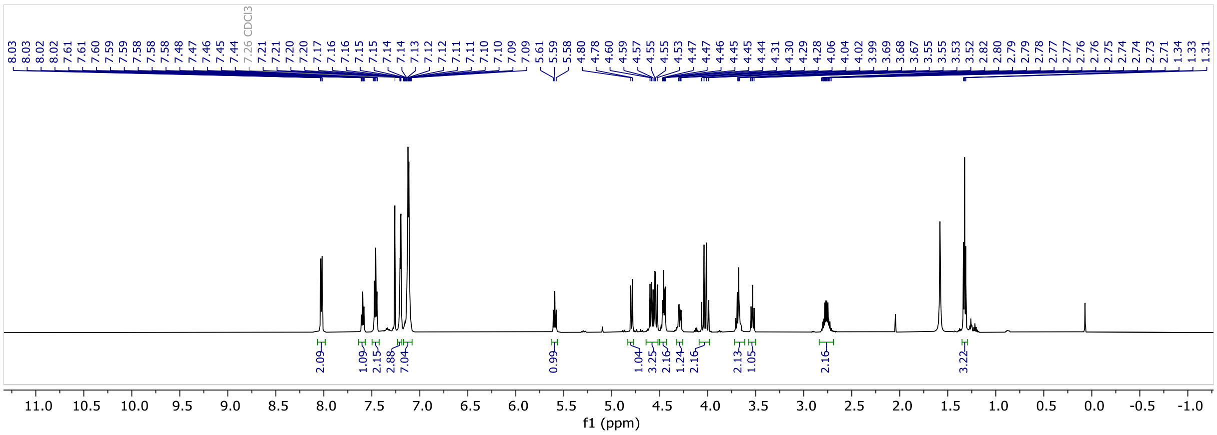

^{13}C NMR (151 MHz, $CDCl_3$) of **5**:

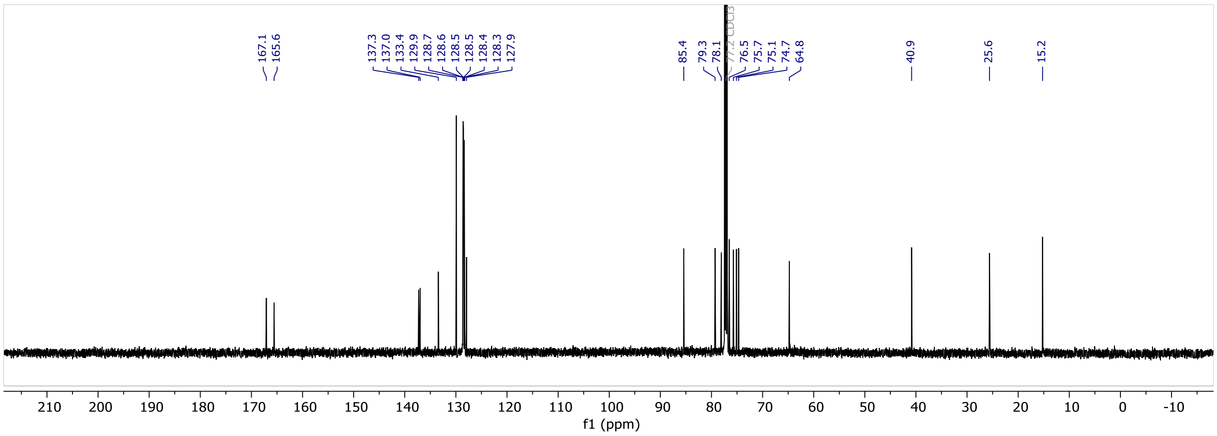

$^{13}C,^{1}H$ HSQC of **5**:

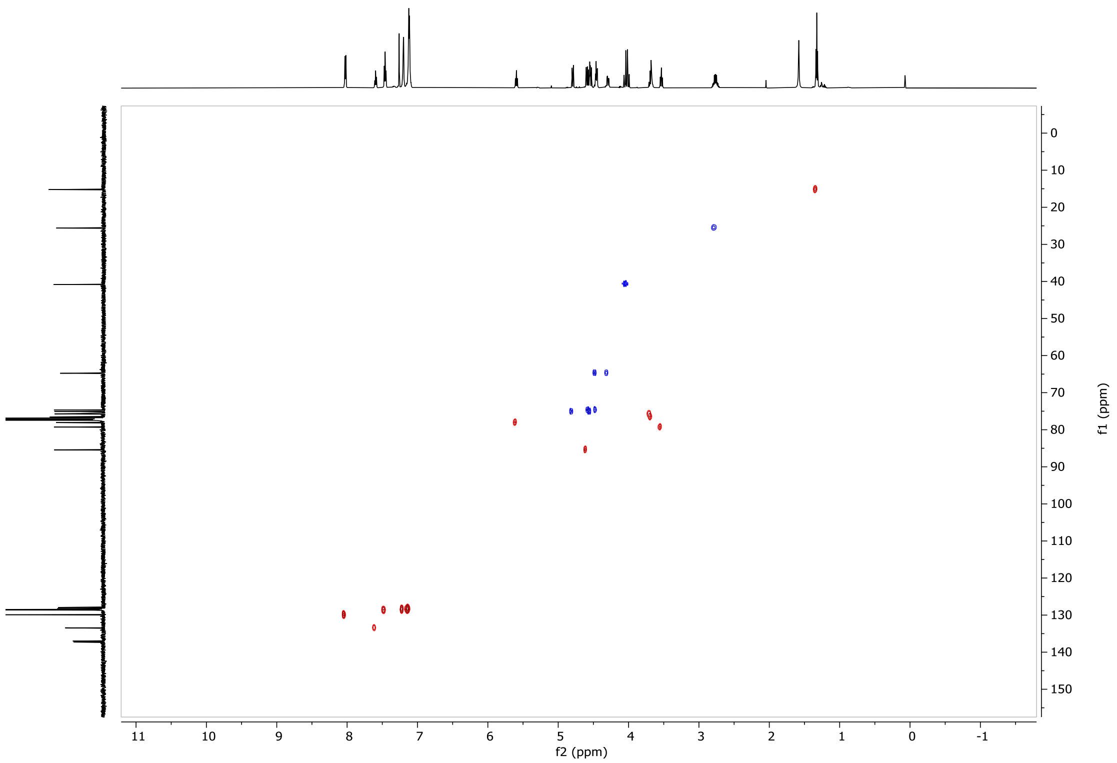

4.4 Galactosamine

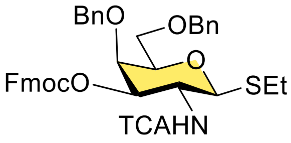

2

Galactosamine building block **2** was purchased by GlycoUniverse GmbH & CO KGaA.

5 Human Sera and Saliva Sample Collection and Characterization

5.1 General Information

Sera, saliva, and subgingival plaque from periodontitis patients (inflamed (I), 16 subjects), treated periodontitis patients (treated (T), 6 subjects) and periodontally healthy patients (healthy (H), 20 subjects) were collected by Dr. Kim N. Stolte and provided by Prof. Dr. Henrik Dommisch. The study groups were defined as follows:

- Healthy (H): Participants without clinical signs of disease, according to the criteria proposed by Chapple et al. (2018)[6].
- Inflamed (I): Participants with periodontal disease in Stage III/IV and Grade B/C, based on the classification of Papapanou et al. (2018)[7].
- Treated (T): Participants who are periodontally stable, as defined by Chapple et al. (2018)[6].

For the collection of saliva, subgingival plaque, and venous blood samples, a positive ethical approval was obtained from the Ethics Committee of Charité Universitätsmedizin Berlin under the application number EA 4/035/22, and the participants gave their signed informed consent.

Sera and saliva were used for the immunological evaluation of the synthesized glycans in glycan microarray experiments. PCR was used to analyze *P. gingivalis* expression in plaque samples.

5.2 Sera Collection

For the collection of sera, 5 mL of venous blood was withdrawn per puncture using serum tubes (Vacuette, 5 mL CAT serum Sep Clot Activator, Greiner). The filled tubes rested vertically

for 30 min at room temperature to ensure coagulation, followed by centrifugation (15 minutes, 1300 x g). The serum was then transferred to Eppendorf tubes and stored at -20 °C until use.

5.3 Saliva Collection

Non-stimulated saliva samples were collected passively by having participants expectorate into sterile containers for 5 minutes. The collected samples were then transferred into Eppendorf tubes and immediately frozen at -20°C until further use.

5.4 PCR analysis of *Porphyromonas gingivalis* in dental plaque biofilm

Subgingival biofilm samples were collected using sterile curettes, which were gently inserted into the sulcus of the participants' teeth. The biofilm was carefully scraped and transferred onto a sterile paper point, which was then placed into a sterile Eppendorf tube. The samples were immediately frozen at -20°C for subsequent analysis.

DNA was extracted using the phenol-chloroform extraction method. qPCR was performed in 10 µl reactions using a Bio-Rad CFX Real-Time Thermocycler (USA). Each reaction contained the following components: 5 µl SYBR Power Up Master Mix, 4 µl primers (0.1 µM forward primer and 0.1 µM reverse primer), and 1 µl template. The thermal cycling protocol included an initial enzyme activation step at 50°C for 2 minutes, followed by 39 cycles consisting of 2 minutes at 95°C for denaturation, 15 seconds at 95°C for denaturation, and 1 minute at 60°C for annealing and extension. A final step included 5 seconds at 95°C followed by 5 seconds at 65°C. Each sample was measured in duplicates, with H_2O used as a negative control. The primers used for amplification of *Porphyromonas gingivalis* were: forward primer (FW) ACCTTACCCGGGATTGAAATG and reverse primer (RW) CAACCATGCAGCACCTACATAGAA.

Table S1. CT values (PCR) of the three patient groups (dental healthy (H), inflamed (I) periodontitis and treated (T). Patients with outlaying high antibody-binding in microarray studies are marked in *orange*.

Dental status: H		Dental status: I		Dental status: T	
Mean(cT) = 24.71		Mean(cT) = 18.52		Mean(cT) = 21.70	
**Sample**	**cT**	**Sample**	**cT**	**Sample**	**cT**
P-H-00	21.82	P-D-01	28.14	P-T-01	*21.09*
P-H-01	*21.90*	P-D-02	23.42	P-T-02	21.95
P-H-02	24.30	P-D-03	16.15	P-T-03	22.13
P-H-03	25.32	P-D-04	19.90	P-T-04	23.65
P-H-04	25.72	P-D-05	18.55	P-T-05	16.15
P-H-05	25.08	P-D-06	22.93	P-T-06	25.22
P-H-06	24.94	P-D-07	*17.62*		
P-H-07	30.85	P-D-08	18.66		
P-H-08	24.52	P-D-09	*17.35*		
P-H-09	22.15	P-D-10	23.54		
P-H-10	25.74	P-D-11	14.53		
P-H-11	23.33	P-D-12	19.23		
P-H-12	24.17	P-D-13	16.71		
P-H-13	25.25	P-D-14	17.94		
P-H-14	24.72	P-D-15	16.72		
P-H-15	25.01	P-D-16	*14.51*		
P-H-16	25.04				
P-H-17	24.76				
P-H-18	25.96				
P-H-19	23.59				

6 Glycan Microarrays

6.1 Printing of Microarray-Slides

Amine-terminated oligosaccharides were immobilized on commercial N-hydroxysuccinimide (NHS) ester-activated microarray slides (CodeLink Activated Slides; SurModics) using a piezoelectric microarray spotting device (S3; Scienion) such that 64 identical subarrays can be contained on each slide. Before the spotting, glycan solutions were prepared (0.1 mM, in 50 mM sodium phosphate buffer, pH 8.5). Slides were incubated in a humid chamber for 24 h at room temperature to complete coupling reactions. The next day, slides were quenched in 100 mM ethanolamine in 50 mM sodium phosphate buffer, pH 9, for one hour at room temperature. Afterwards, the slides were washed with deionized water (3 x), dried by centrifugation (300 x g, 5 min) and stored at 4 °C in a black box until use.

6.2 Glycan Microarray Experiments

The microarray slides with immobilized glycans were blocked with 1% BSA-PBS for one hour at room temperature. Subsequently, the slides were washed with PBS (3 x) and dried by centrifugation. A FlexWell 64 grid was applied and slides were incubated with human serum (dilution 1:10 in 1% BSA-PBS) and saliva (undiluted) in triplicate in a humid chamber for one hour at room temperature. Wells were washed with PBS-Tween (3 x) followed by incubation with fluorescence-labeled secondary antibody (Goat anti-Human IgG Fc-AF 647, SouthernBiotech) in a light-protected humidity chamber for 60 minutes at room temperature.

The slides were washed three times with PBS-Tween (3 x). Then, the grids were removed and washed in a Petri dish with PBS-Tween for 2 minutes and rinsing (total 3 x) followed by washing with deionized water for 2 minutes and rinsing (total 3 x). The slides were dried by centrifugation (300 x g, 5 min). Afterwards, the slides were scanned with a GenePix 4300A microarray scanner (Molecular Devices, Sunnyvale, CA, USA). The Image analysis was carried out using GenePix Pro 7 software (Molecular Devices).

6.3 Statistical Analysis

Graphing and statistical analysis (two-sided unpaired *t* tests) were performed using OriginPro® 2021b.

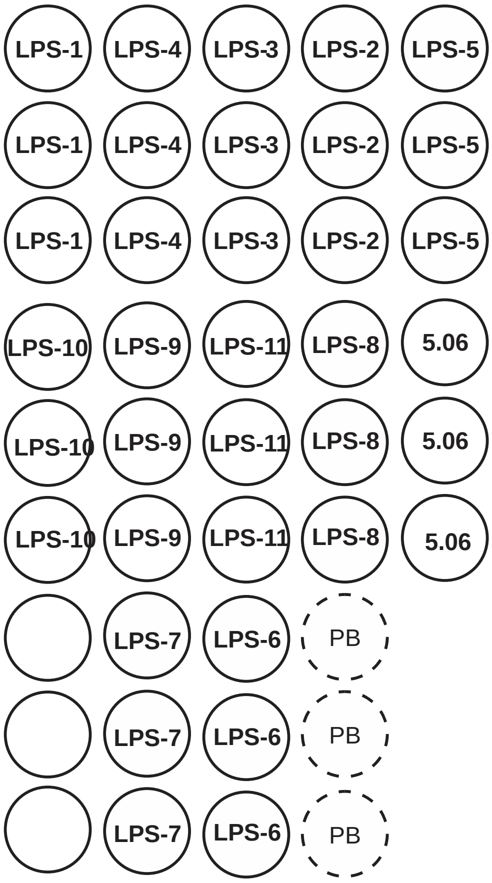

Scheme S1: Microarray printing pattern of *P. gingivalis* glycans **LPS1-11** and **5.06** were printed at a concentration of 0.1 mM. PB – Printing buffer.

6.4 Further Microarray Data

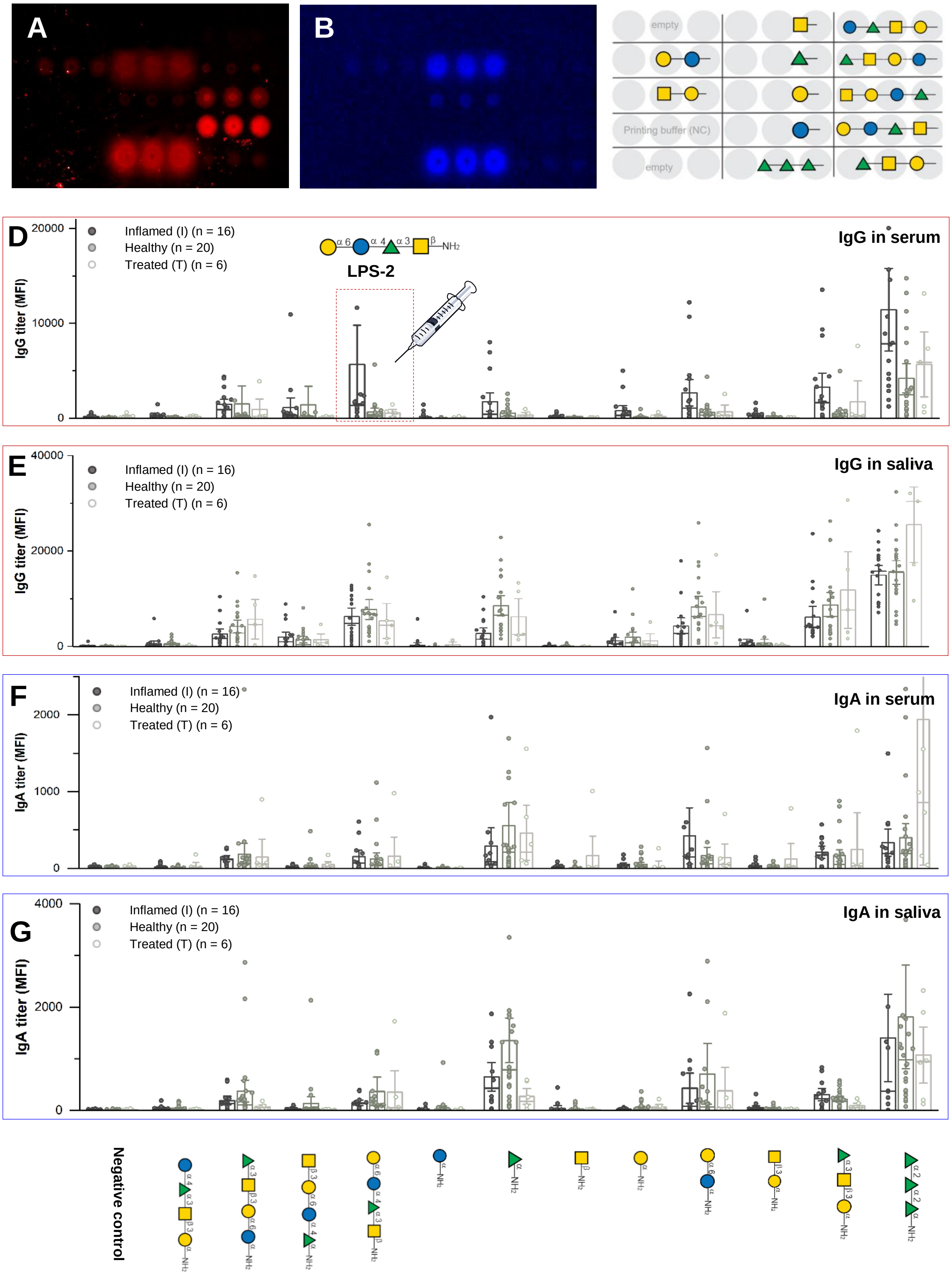

Figure S1. Determination of human IgG and IgA antibodies binding to synthesized LPS-fragments **LPS1-11** and positive control **5.06**. (**A**) Fluorescence signals indicating IgG antibody binding in saliva of one exemplary inflamed periodontitis (I) patient, (**B**) fluorescence signals indicating IgA antibody binding in saliva of one exemplary healthy,

and (**C**) associated printing pattern. (**D-G**) Mean fluorescence intensity (MFI) indicating IgG or IgA titer of bound human IgG or IgA antibodies in saliva or serum to glycan library.

6.5 *In vivo* and *in vitro* testing of vaccine candidates LPS-2-CRM_{197} and LPS-5-CRM_{197}

LPS conjugation to carrier protein

The selected lead vaccine saccharides (LPS-2: 0.7 mg; LPS-5: 0.8 mg) were dissolved in 200 µL DMSO, 25 µL Pyridine and 10 µL Triethylamine were added. 6.5 equivalents of a (4-nitrophenyl)-adipate linker in DMSO were added to the saccharide solution and stirred for 3 h. The reaction mixture was frozen in liquid nitrogen, followed by lyophilization which resulted in a crude white solid. Washing with chloroform (5x 100 µL) and DCM (3x 1 µL) removed excess of bis (4-nitrophenyl) adipate. It was checked by TLC that all excess linker was washed away and no glycan dissolved. A 10k Amicon filter (Merck Millipore) was wet by adding 350 µL of water and centrifuged at 10000 rpm for 8 min. Then CRM197 was transferred into the Amicon 10k filter (180 µL, 1 mg) tube, washed with 300 µL of water. The CRM197 vial was centrifuged at 10000 rpm for 8 min, washed with 350 µL water and transferred to the filter. After another round of centrifugation the CRM197 vial was washed with 350 µL of 0.1 M phosphate buffer pH 8. After centrifugation, the filter was removed, turned upside down into a clean vial and centrifuged for 2 min at 1,000 rpm, giving a colorless filtrate containing CRM197. The filtrate (~70 µL) was transferred into the vial containing the sugar PNP ester and the tube was washed with phosphate buffer pH 8 (2 x 30 µL) to remove remaining filtrate and added to the reaction solution. After slow stirring for 24 h, the solution was transferred onto an Amicon 10K filter and the reaction vial was washed with 300 µL of the reaction buffer. Afterwards, the solution was washed three times with 400 µL water and once with 400 µL PBS. Finally, the filter was putted upside down into a clean vial and centrifuged at 1,000 rpm for 2 min. The filtrate was diluted by adding 350 µL PBS and stored at -20°C (**Figure S2 (A)**).

Resulting glycoconjugates were characterized by polyacrylamide gel electrophoresis (**Figure S2 (B)**). An alkaline separating gel (375 mM Tris/HCl pH 8.8, 12% (w/v) of a 29:1 acrylamide/N,N'-methylenebisacrylamide mixture) and an acidic stacking gel (100 mM Tris/HCl pH 6.8, 4.5% (w/v) of a 29:1 acrylamide/N,N'- methylenebisacrylamide mixture), polymerized by the addition of TEMED and 10% (w/v) ammonium peroxodisulfate, were prepared. glycoconjugate and CRM197 samples were dissolved in SDS-PAGE loading buffer (250 mM Tris (pH 6.8), 5 % (v/v) beta-mercaptoethanol, 10 % (w/v) SDS, 0.4% bromophenol blue, 50 % (v/v) glycerol) and 1 µg of glycoconjugate were loaded. As a size reference, 4µL of PageRuler Plus Prestained Protein Ladder 10 to 250 kDa (Thermo Scientific) was used. The samples

were run at 80 V and 25 mA for 15 min and then at 120 V and 25 mA for 90 min and stained with 0.5 % (w/v) Coomassie Brilliant Blue R-250 for 30 min.

Samples were further characterized by MALDI-TOF-MS (**Figure S2 (D, E)**). Mass spectra were acquired with an Autoflex Speed MALDI-TOF system (Bruker Daltonics; Bremen, Germany). Samples were spotted on MTP 384 ground steel target plates (Bruker Daltonics) using the dried droplet technique with 2,5-dihydroxyacetophenone (DHAP) as matrix. The mass spectrometer was operated in linear positive mode. Mass spectra were acquired over a m/z range of 30,000 to 210,000. The data was analysed using the FlexAnalysis software supplied with the instrument.

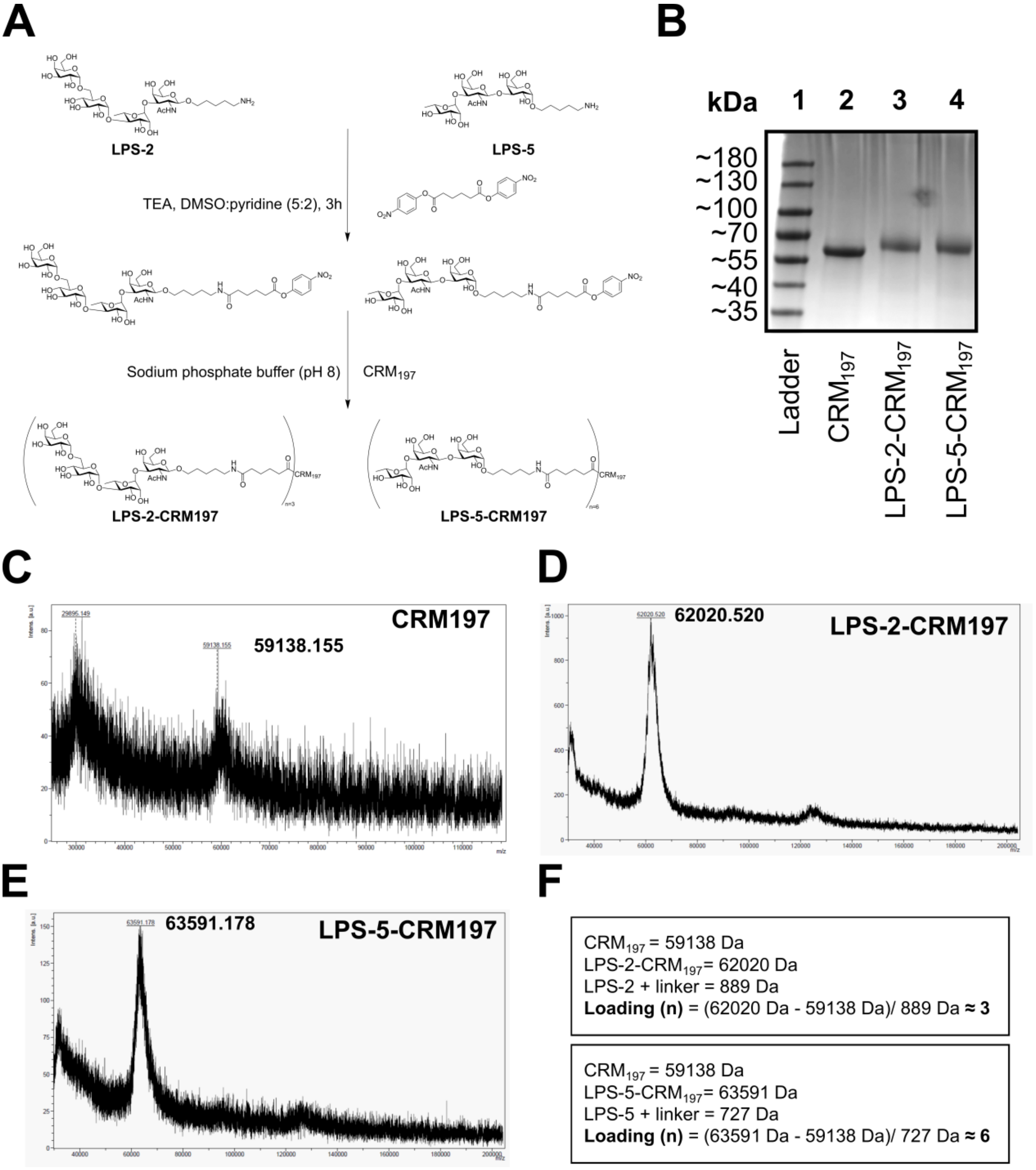

Figure S2. Characterization of the LPS-2-CRM_{197} and LPS-5-CRM_{197} glycoconjugates. (A) Coupling of LPS-2 and LPS-5 to the carrier protein CRM_{197} resulted in the glycoconjugate LPS-2-CRM_{197} and LPS-5-CRM_{197} respectively. (B) SDS-PAGE of carrier protein (lane 2) und glycoconjugates (lane 3 and 4) and PageRuler™ Plus Prestained Protein Ladder (lane 1). MALDI-TOF MS spectra of CRM_{197} (C) and glycoconjugate LPS-2-CRM_{197} (D) and LPS-5-CRM_{197}. (E) Calculation to estimate the loading of each carrier protein CRM197 with LPS-2 or LPS-5.

Immunization

The immunizations were approved by Landesamt für Gesundheit und Soziales Berlin, Germany (Approval ID: G 0329/18). Eight-week-old C57BL/6NRj mice (Janvier, Le Genest-Saint-Isle, France) were housed in groups of five in individually ventilated cages (Tecniplast, Hohenpeißenberg, Germany) at the Federal Institute for Risk Assessment, Berlin, Germany, under specified pathogen-free conditions. Housing and experiments were in accordance with

institutional guidelines and with the regulations of the Federation of European Laboratory Animal Science Associations (FELASA).

Mice were immunized by subcutaneous (s.c.) injection with 1 µg of glycoconjugate per injection. The glycoconjugates were diluted with alum adjuvant in sterile PBS to a final volume of 100 µL per dose. Animals were immunized on day 0 (primary immunization), and boosted on days 14 and 28, with a final boost on day 196. Blood (max. 80 µL) and saliva samples were collected every two weeks. Mice were restrained by hand and a swab was used to collect saliva, which was then centrifuged at 400 g for 5 min. After coagulation at room temperature, the serum was separated from the blood clot by centrifugation at 2,000 g for 10 min. Sera and saliva were frozen at -20°C until further use.

Glycan Microarray Experiments with mice sera and saliva.

Microarray-Slides were printed as in described in section 6.1 with a slightly differing printing pattern (**Scheme S2**). Synthetic polysaccharides containing an amine linker as well as commercial LPS-PG (InvivoGen), CRM and Human and Murine IgG and IgM isotype controls (SouthernBiotech) were immobilized on glass microarray slides.

Slides were then quenched, blocked with 1% BSA-PBS, and stored at 4°C until use. A FlexWell 64 grid was attached and the slides were incubated with mouse sera diluted 1:100 or pooled saliva samples diluted 1:5 in 1% BSA-PBS (w/v) for 1 hour at 37°C in a humidification chamber. After three washes with PBST, the slides were incubated with fluorescently labeled secondary antibodies (Alexa Fluor® 488 AffiniPure goat anti-mouse IgG, Fcγ fragment specific, Jackson ImmunoResearch; goat anti-mouse IgM (heavy chain) secondary antibody, Alexa Fluor™ 647, Invitrogen; goat anti-mouse IgA alpha chain (DyLight® 594), Abcam), each diluted 1:400 in blocking buffer. Slides were incubated for 1 hour at 37°C in a humidification chamber, washed three times with PBST, rinsed with deionized water, and dried by centrifugation. Fluorescence signals were measured using a Genepix 4300A instrument (Molecular Devices, Sunnyvale, CA, USA). The photomultiplier tube (PMT) voltage was adjusted to avoid oversaturation of the signals. Data were analyzed using GenePix Pro 7 software (Molecular Devices, Sunnyvale, CA, USA).

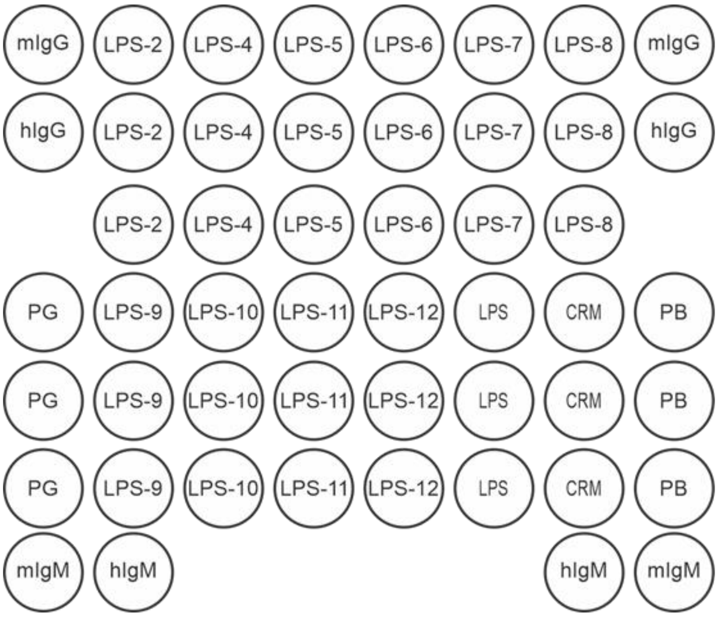

Scheme S2. Microarray printing pattern of *P. gingivalis* glycans to test mice sera and saliva samples, glycans 0.1 mM printing concentration, proteins 100 µg/mL. PB – Printing buffer. PG: peptidoglycan, mIgG: murine IgG isotype control, hIgG: human IgG isotype control, mIgM: murine IgM, hIgM: human IgM, LPS: commercial LPS , CRM: CRM197

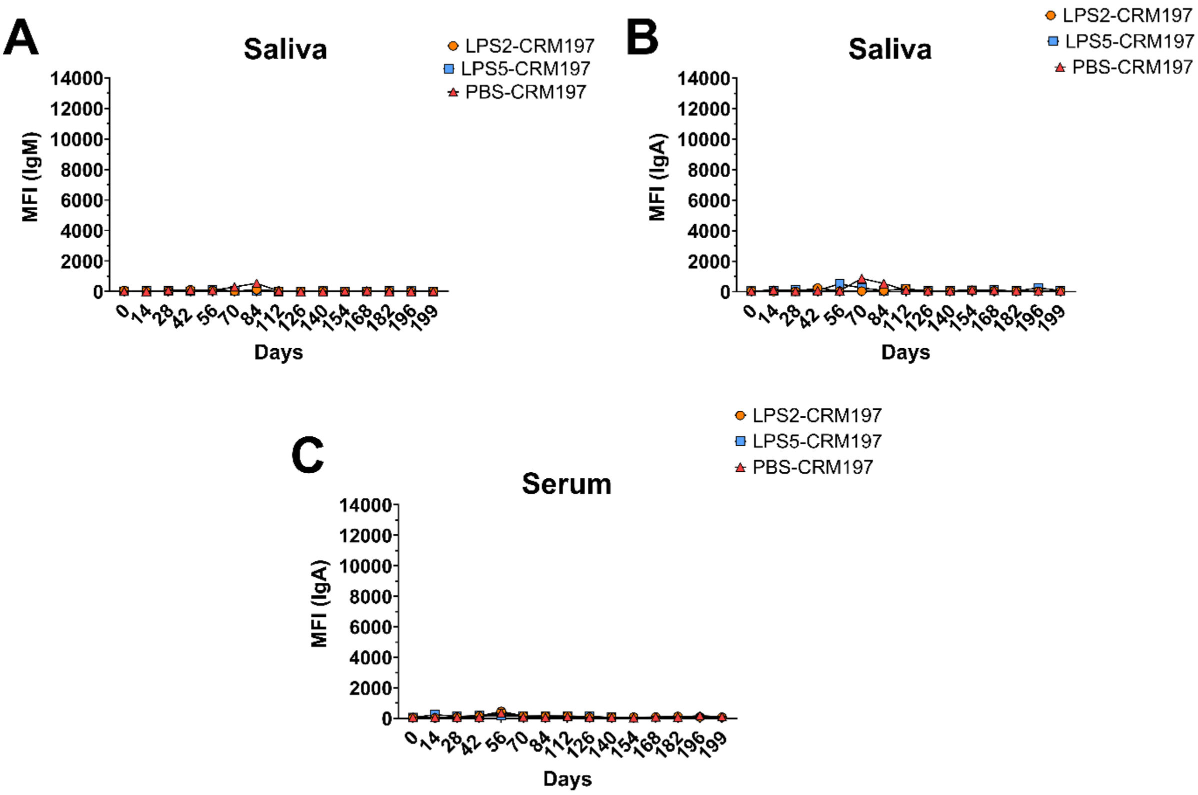

Figure S3. Glycan microarray analysis of the saliva (A, B) and serum (C) of the immunized mice. Mean fluorescence intensity (MFI) of IgM antibodies (A) and IgA antibodies (B, C) directed towards the respective glycan.

***P. gingivalis* W50 culture**

P. gingivalis W50 (ATCC-53978) bacteria were cultured on Columbia Blood agar plates (VWR, Belgium) under anaerobic conditions for 5 to 7 days. For liquid cultures, bacteria were grown overnight in Supplemented Tryptic Soy Broth (ATCC medium 2722). Anaerobic conditions were established in an anaerobic culture vessel supplemented with a 2.5 L Thermo Scientific™ Oxoid™ AnaeroGen™ package (Thermo Fisher Scientific).

The 100 mL ATCC Medium 2722 of consists of 30.0 g tryptic soy broth, 5.0 g yeast extract, 0.5 g L-cysteine hydrochloride, 1.0 mL heme stock (5 µg/mL), 0.5 g heme, 1.74 g K2HPO4, 0.2 mL vitamin K3 stock (5 mg/mL; final concentration 1.0 µg/mL), and 100 mL distilled water. After preparation, the medium was autoclaved at 121°C.

Bacterial ELISA with *P. gingivalis* W50 Bacteria were cultured overnight in supplemented tryptic soy broth to obtain different ODs. Bacteria were grown to an OD of 0.7 (~9.4 x 10^{10} cfu) and centrifuged at 3000g for 5 minutes. The supernatant was discarded. Bacteria were washed three times with PBS and then diluted in 10 mL of carbonate-bicarbonate coating buffer. High binding flat bottom ELISA plates (Corning) were coated with 100 µl of diluted bacteria and incubated overnight at 4°C. The ELISA plates were washed 3 times with PBST. Sera from day 56 after immunization were diluted 1:100 in PBS/BSA. 50 µl of the serum dilution in triplicates were pipetted into the appropriate wells and incubated at RT for 2 hours. The plates were washed 3 times with PBST.

50 µl of goat anti-mouse IgG HRP antibody (Dianova) at 1:10000 dilution was added to each well and incubated for 3 hours. The plates were washed 3 times with PBST and 100 µl of substrate TMB was added to each well. After 10 minutes, the reaction was stopped by adding H2SO4. Absorbance was read at 450 nm in a CLARIOstar Plus plate reader (BMG Labtech).

Statistical Analysis

Graphing and statistical analysis (one sample t-test) were performed using GraphPad Prism 9.3.1.

References

(1) Le Mai Hoang, K.; Pardo-Vargas, A.; Zhu, Y.; Yu, Y.; Loria, M.; Delbianco, M.; Seeberger, P. H. Traceless Photolabile Linker Expedites the Chemical Synthesis of Complex Oligosaccharides by Automated Glycan Assembly. Journal of the American Chemical Society **2019**, 141 (22), 9079-9086. DOI: 10.1021/jacs.9b03769.

(2) Gude, M.; Ryf, J.; White, P. D. An accurate method for the quantitation of Fmoc-derivatized solid phase supports. Letters in Peptide Science **2002**, 9 (4-5), 203-206.

(3) Elsaidi, H. R.; Lowary, T. L. Effect of phenolic glycolipids from Mycobacterium kansasii on proinflammatory cytokine release. A structure–activity relationship study. Chemical science **2015**, 6 (5), 3161-3172.

(4) Tian, G.; Hu, J.; Qin, C.; Li, L.; Zou, X.; Cai, J.; Seeberger, P. H.; Yin, J. Chemical Synthesis and Immunological Evaluation of Helicobacter pylori Serotype O6 Tridecasaccharide O-Antigen Containing a dd-Heptoglycan. Angewandte Chemie International Edition **2020**, 59 (32), 13362-13370, https://doi.org/10.1002/anie.202004267. DOI: https://doi.org/10.1002/anie.202004267 (acccessed 2021/07/07).

(5) Mydock, L. K.; Kamat, M. N.; Demchenko, A. V. Direct synthesis of diastereomerically pure glycosyl sulfonium salts. Organic letters **2011**, 13 (11), 2928-2931.

(6) Chapple, I. L. C.; Mealey, B. L.; Van Dyke, T. E.; Bartold, P. M.; Dommisch, H.; Eickholz, P.; Geisinger, M. L.; Genco, R. J.; Glogauer, M.; Goldstein, M.; et al. Periodontal health and gingival diseases and conditions on an intact and a reduced periodontium: Consensus report of workgroup 1 of the 2017 World Workshop on the Classification of Periodontal and Peri-Implant Diseases and Conditions. J Periodontol **2018**, 89 Suppl 1, S74-S84. DOI: 10.1002/JPER.17-0719

(7) Papapanou, P. N.; Sanz, M.; Buduneli, N.; Dietrich, T.; Feres, M.; Fine, D. H.; Flemmig, T. F.; Garcia, R.; Giannobile, W. V.; Graziani, F.; et al. Periodontitis: Consensus report of workgroup 2 of the 2017 World Workshop on the Classification of Periodontal and Peri-Implant Diseases and Conditions. J Periodontol **2018**, 89 Suppl 1, S173-S182. DOI: 10.1002/JPER.17-0721
